# Supplementary material for: A moisture-tolerant route to unprotected α/β-amino acid N-carboxyanhydrides and facile synthesis of hyperbranched polypeptides
Source: Nat Commun. 2021 Oct 4;12:5810. doi: 10.1038/s41467-021-25689-y (PMC8490447; doi:10.1038/s41467-021-25689-y)
Supplement: Supplementary file 1 — Supplementary Information [file 41467_2021_25689_MOESM1_ESM.pdf]

Supplementary Information

A Moisture-Tolerant Route to Unprotected α/β-Amino Acid *N*-Carboxyanhydrides and Facile Synthesis of Hyperbranched Polypeptides

Zi-You Tian,[1] Zhengchu Zhang,[1] Shuo Wang,[1] and Hua Lu[1,*]

[1]Beijing National Laboratory for Molecular Sciences, Center for Soft Matter Science and Engineering, Key Laboratory of Polymer Chemistry and Physics of Ministry of Education, College of Chemistry and Molecular Engineering, Peking University, Beijing 100871, People's Republic of China.

Email: chemhualu@pku.edu.cn

Contents

Supplementary Methods **1**
Supplementary Figures and Tables **20**
Supplementary References **89**

Supplementary Methods

Materials

All of the reagents were purchased from commercial sources and used as received. **Without specific illustration, all of the solvents were analytically pure**. **The silica gel was dried under 96 ºC for 3 – 4 h in an oven before use.**

Reagent	Manufactory
THF (AR)	*Concord Technology* (Tianjin, China)
DMF (AR)	*Concord Technology* (Tianjin, China)
acetonitrile (AR)	*Concord Technology* (Tianjin, China)
triphosgene	*J&K* (Beijing, China)
propylene oxide	*HUSHI* (Shanghai, China)
epichlorohydrin	*HUSHI* (Shanghai, China)
γ-benzyl L-glutamate	*Konoscience* (Beijing, China)
L-Cbz-Lysine	*Konoscience* (Beijing, China)
ε-trifluoroacetyl L-lysine	*J&K* (Beijing, China)
L-alanine	*HUSHI* (Shanghai, China)
L-phenylalanine	*Heowns* (Tianjin, China)
L-valine	*J&K* (Beijing, China)
L-leucine	*J&K* (Beijing, China)
L-tryptophan	*Konoscience* (Beijing, China)
L-tyrosine	*GL Biochem* (Shanghai, China)
glycine	*HUSHI* (Shanghai, China)
L-methionine	*3A Chem* (Shanghai, China)
O-tert-butyl L-serine	*Konoscience* (Beijing, China)
ε-*N*-Boc L-lysine	*Konoscience* (Beijing, China)
L-serine	*J&K* (Beijing, China)
L-threonine	*J&K* (Beijing, China)
L-cysteine	*Aladdin* (Shanghai, China)
D-penicillamine	*Yuanye Bio-Technology* (Shanghai, China)
L-glutamic acid	*HUSHI* (Shanghai, China)
Boc-sarcosine	*J&K* (Beijing, China)
Boc-L-proline	*GL Biochem* (Shanghai, China)
Boc-L-hydroxyproline	*Bidepharm (*Shanghai, China)
L-phenyllactic acid	*Konoscience* (Beijing, China)
mandelic acid	*J&K* (Beijing, China)
β-alanine	*9dingchem* (Shanghai, China)
β-aminobutyric acid	*MREDA* (Beijing, China)
L-aspartic acid β-methyl ester	*Konoscience* (Beijing, China)
L-aspartic acid β-methyl ester	*Konoscience* (Beijing, China)
3-amino-3-(*p*-tolyl)propanoic acid	*Konoscience* (Beijing, China)

Reagent	Manufactory
3-amino-3-(4-chlorophenyl)propanoic acid	*OKA* (Beijing, China)

Content of water of solvents

Entry	Content of water in ppm
THF-d_8 used in Figure 1A (CIL Inc)	< 200 ppm
THF used for NCA preparation and recrystallization (analytical grade, *Concord Technology Inc.* (Tianjin, China))	< 2040 ppm
DMF used for PLG preparation (anhydrous, 99.8%, *Sigma-Alderich, Merck, (Germany*)	< 200 ppm
ACN used for NCA preparation and recrystallization (analytical grade, *Concord Technology Inc.* (Tianjin, China))	< 1100 ppm
PE used for recrystallization and column chromatography (analytical grade, *Tong Guang Chem. Inc.*, Beijing, China)	< 700 ppm
Ethyl acetate used for column chromatography (analytical grade, *Tong Guang Chem. Inc.*, Beijing, China)	< 2300 ppm

Characterizations

Nuclear magnetic resonance (NMR) spectra were measured on Bruker AVANCE III 400 (^{1}H at 400 MHz; ^{13}C at 101 MHz) or Bruker AVANCE III HD 400 (^{1}H at 400 MHz; ^{13}C at 101 MHz) NMR spectrometers. ^{1}H NMR spectra data are reported as follows: chemical shift δ (ppm) referenced to either $CDCl_3$ (7.26 ppm), THF-d_8 (1.72 ppm), DMSO-d_6 (2.50 ppm) or D_2O (4.79 ppm), multiplicity (s = singlet, d = doublet, t = triplet, q = quartet, m = multiplet, dd = doublet of doublets, dt = doublet of triplets, ddd = doublet of doublet of doublets), coupling constant J (Hz), and integration. ^{13}C NMR spectra data are reported as follows: chemical shift δ (ppm) referenced to either $CDCl_3$ (77.16 ppm), DMSO-d_6 (39.52 ppm), THF-d_8 (25.31 ppm) or tetramethylsilane (TMS, 0.00 ppm). High-resolution mass spectrometry (HRMS) data were recorded on Bruker Apex IV or Bruker Solarix XR fourier transform ion cyclotron resonance (FTICR) mass spectrometers (electrospray ionization, ESI). To obtain the absolute molecular weight (M_n) and dispersity ($Đ$) of PLG, the aqueous SEC was performed on an ÄKTA equipped with a differential refractive index detector Optilab T-rEX (658.0 nm) and a light scattering detector miniDAWN TREOS (Wyatt, USA). The dn/dc value of PLG in PBS was measured as 0.1184 ± 0.002859 mL/g.

Glassware

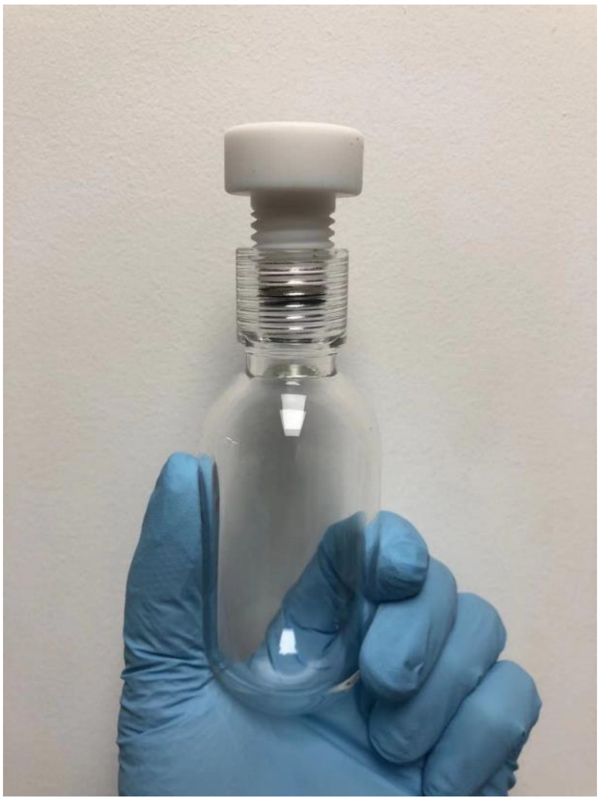

!!!NOTE: Pressure vessel with heavy wall was recommended to prevent potential phosgene and PO leakage/escape.

3b: L-Cbz-Lysine NCA

Air humidity: 56%

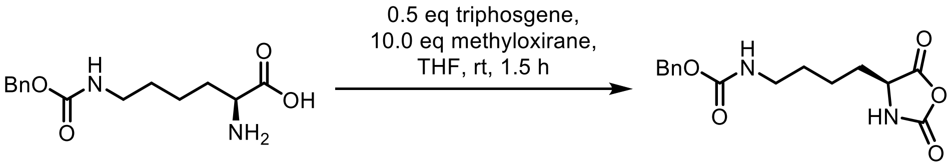

1b
1.0 g

3b
929 mg, yield = 85%

To a pressure vessel with heavy wall, Cbz - L-lysine **1b** (1.0 g, 3.6 mmol, 1.0 eq), THF (15 mL), methyloxirane (2.5 mL, 36 mmol, 10.0 eq) were added sequentially under magnetic stirring. Triphosgene (534 mg, 1.8 mmol, 0.5 eq) was finally added in one portion and the vessel was sealed immediately. The reaction was stirred at room temperature for ~1.5 h in total and cooled down to ~ 4 °C in an ice bathe. **For safety reason, the excessive triphosgene** was quenched by adding 10 mL **cold water** at ~ 4 °C with 1-3 min stirring. The mixture was extracted with ethyl acetate (EA, 20 mL × 2) at room temperature. The combined organic phase was washed with brine and dried with anhydrous Na_2SO_4. After the removal of solvent by rotatory evaporation *in vacuum under 45 °C*, the crude product was purified by crystallization in hexane/THF **below 10 °C (preferably in a cold room)** without N_2 protection. The pure product **3b** was obtained as a white crystal (929 mg, yield =

85%). The NCA was stored at -10 °C for 2 months.

^{1}H NMR (400 MHz, DMSO-*d*$_6$) δ 9.08 (s, 1H), 7.40 – 7.28 (m, 5H), 7.26 (t, *J* = 6.1 Hz, 1H), 5.00 (s, 2H), 4.43 (dd, *J* = 7.4, 5.1 Hz, 1H), 2.99 (dt, *J* = 6.1, 6.1 Hz, 2H), 1.80 – 1.58 (m, 1H), 1.48 – 1.22 (m, 4H).
^{13}C NMR (101 MHz, DMSO-*d*$_6$) δ 171.7, 156.1, 152.0, 137.3, 128.4, 127.8, 127.8, 124.2, 65.2, 57.0, 30.7, 28.8, 21.6.
HRMS (ESI−FTICR, *m/z*): [M + H]$^+$ calculated for $C_{15}H_{19}N_2O_5^+$: 307.1288; found: 307.1287.
FT-IR (cm^{-1}) 3340, 1857, 1775.

3c: ε-trifluoroacetyl L-lysine NCA

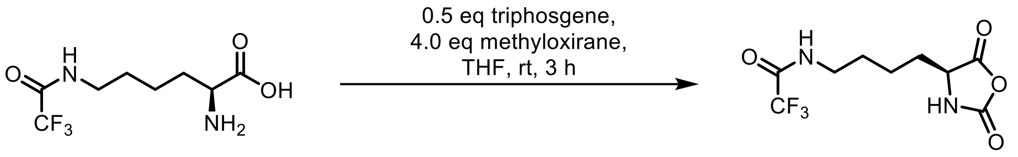

1c
1.0 g

3c
991 mg, yield = 88%

To a pressure vessel with heavy wall, ε-trifluoroacetyl L-lysine **1c** (1.0 g, 4.2 mmol, 1.0 eq), THF (20 mL), methyloxirane (1.2 mL, 17.2 mmol, 4.0 eq) were added sequentially under magnetic stirring. Triphosgene (624 mg, 2.1 mmol, 0.5 eq) was finally added in one portion and the vessel was sealed immediately. The reaction was stirred at room temperature for ~3 h in total. After the removal of solvent by rotatory evaporation *in vacuum under 45 °C*, the crude product was purified by crystallization in hexane/THF **below 10 °C (preferably in a cold room)** without N_2 protection. The pure product **3c** was obtained as a white crystal (991 mg, yield = 88%). The NCA was stored at -10 °C for 2 months.

^{1}H NMR (400 MHz, DMSO-*d*$_6$) δ 9.42 (t, *J* = 6.5 Hz, 1H), 9.10 (s, 1H), 4.43 (t, *J* = 6.5 Hz, 1H), 3.17 (q, *J* = 6.5 Hz, 2H), 1.75 (ddt, *J* = 15.5, 10.6, 5.2 Hz, 1H), 1.69 – 1.59 (m, 1H), 1.49 (p, *J* = 7.4 Hz, 2H), 1.43 – 1.34 (m, 1H), 1.34 – 1.21 (m, 1H).
^{13}C NMR (101 MHz, DMSO-*d*$_6$) δ 171.7, 156.8, 156.4, 156.0, 155.7, 152.0, 120.3, 117.4, 114.6, 111.7, 57.0, 30.6, 27.7, 21.6.
HRMS (ESI−FTICR, *m/z*): [M + H]$^+$ calculated for $C_9H_{12}F_3N_2O_4^+$: 269.0744; found: 269.0737.
FT-IR (cm^{-1}) 1857, 1792, 1725.

3d: L-Alanine NCA
Air humidity: 68%

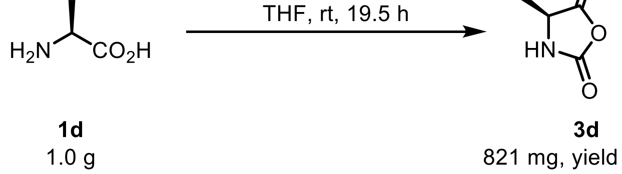

1d
1.0 g

3d
821 mg, yield = 64%

To a pressure vessel with heavy wall, L-alanine **1d** (1.0 g, 11.2 mmol, 1.0 eq), THF (30 mL), methyloxirane (8.0 mL, 112.2 mmol, 10.0 eq) were added sequentially under magnetic stirring.

Triphosgene (1.7 g, 5.6 mmol, 0.5 eq) was finally added in one portion and the vessel was sealed immediately. The reaction was stirred at room temperature for ~19.5 h in total and cooled down to ~ 4 °C in an ice bathe. **For safety reason, the excessive triphosgene** was quenched by adding 10 mL **cold water** at ~ 4 °C with 1-3 min stirring. The mixture was extracted with ethyl acetate (EA, 20 mL × 2) at room temperature. The combined organic phase was washed with brine and dried with anhydrous Na_2SO_4. After the removal of solvent by rotatory evaporation *in vacuum*, the crude product was purified by crystallization in hexane/THF **below 10 °C (preferably in a cold room)** without N_2 protection. The pure product **3d** was obtained as white needle crystal (821 mg, yield = 64%). The NCA was stored at -10 °C for 2 months.

^{1}H NMR (400 MHz, $CDCl_3$) δ 6.76 (br, 1H), 4.42 (qd, *J* = 7.0, 1.0 Hz, 1H), 1.56 (d, *J* = 7.0 Hz, 3H).
^{13}C NMR (101 MHz, $CDCl_3$) δ 170.3, 152.6, 53.5, 17.8.
HRMS (ESI−FTICR, *m/z*): $[M + NH_4]^+$ calculated for $C_4H_9N_2O_3^+$: 133.0608; found: 133.0607.
FT-IR (cm^{-1}) 3184, 1858, 1790.

3e: L-Phenylalanine NCA

Air humidity: 63%

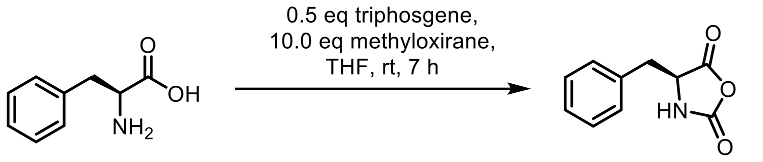

1e
1.0 g

3e
961 mg, yield = 83%

To a pressure vessel with heavy wall, L-phenylalanine **1e** (1.0 g, 6.1 mmol, 1.0 eq), THF (15 mL), methyloxirane (4.3 mL, 60 mmol, 10.0 eq) were added sequentially under magnetic stirring. Triphosgene (897 mg, 4.0 mmol, 0.5 eq) was finally added in one portion and the vessel was sealed immediately. The reaction was stirred at room temperature for 7 h in total and cooled down to ~ 4 °C in an ice bathe. **For safety reason, excessive triphosgene** was quenched by adding 10 mL **cold water** at ~ 4 °C with 1-3 min stirring. The mixture was extracted with ethyl acetate (EA, 20 mL × 2) at room temperature. The combined organic phase was washed with brine and dried with anhydrous Na_2SO_4. After the removal of solvent by rotatory evaporation *in vacuum under 45 °C*, the crude product was purified by crystallization in hexane/THF **below 10 °C (preferably in a cold room)** without N_2 protection. The pure product **3e** was obtained as a white needle crystal (961 mg, yield = 83%). The NCA was stored at -10 °C for 2 months.

^{1}H NMR (400 MHz, $CDCl_3$) δ 7.38 – 7.27 (m, 3H), 7.17 (dd, *J* = 7.7, 1.8 Hz, 2H), 6.48 (br, 1H), 4.53 (ddd, *J* = 7.8, 4.3, 1.0 Hz, 1H), 3.24 (dd, *J* = 14.2, 4.3 Hz, 1H), 3.01 (dd, *J* = 14.2, 7.8 Hz, 1H).
^{13}C NMR (101 MHz, $CDCl_3$) δ 168.9, 152.1, 134.0, 129.3, 128.1, 59.0, 37.9.
HRMS (ESI−FTICR, *m/z*): $[M + NH_4]^+$ calculated for $C_{10}H_{13}N_2O_3^+$: 209.0921; found: 209.0919.
FT-IR (cm^{-1}) 1848, 1775.

3f: L-valine NCA

Air humidity: 45%

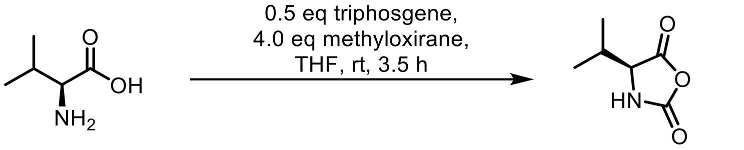

1f
1.0 g

3f
890 mg, yield = 72%

To a pressure vessel with heavy wall, L-valine **1f** (1.0 g, 8.6 mmol, 1.0 eq), THF (20 mL), methyloxirane (2.4 mL, 34.3 mmol, 4.0 eq) were added sequentially under magnetic stirring. Triphosgene (1.3 g, 4.4 mmol, 0.5 eq) was finally added in one portion and the vessel was sealed immediately. The reaction was stirred at room temperature for 3.5 h in total and cooled down to ~ 4 °C in an ice bathe. **For safety reason, the excessive triphosgene** was quenched by adding 10 mL **cold water** at ~ 4 °C with 1-3 min stirring. The mixture was extracted with ethyl acetate (EA, 20 mL × 2) at room temperature. The combined organic phase was washed with brine and dried with anhydrous Na_2SO_4. After the removal of solvent by rotatory evaporation *in vacuum under 45 °C*, the crude product was purified by crystallization in hexane/THF **below 10 °C (preferably in a cold room)** without N_2 protection. The pure product **3f** was obtained as a white needle crystal (890 mg, yield = 72%). The NCA was stored at -10 °C for 2 months.

^{1}H NMR (400 MHz, $CDCl_3$) δ 6.85 (br, 1H), 4.22 (dd, *J* = 4.2, 1.0 Hz, 1H), 2.25 (heptd, *J* = 6.9, 4.2 Hz, 1H), 1.08 (d, *J* = 6.9 Hz, 3H), 1.03 (d, *J* = 6.9 Hz, 3H).
^{13}C NMR (101 MHz, $CDCl_3$) δ 169.1, 153.7, 63.2, 30.9, 18.3, 16.7.
HRMS (ESI−FTICR, *m/z*): $[M + H]^+$ calculated for $C_6H_{10}NO_3^+$: 144.0655; found: 144.0656.
FT-IR (cm^{-1}) 3296, 1839, 1752.

3g: L- leucine NCA
Air humidity: 45%

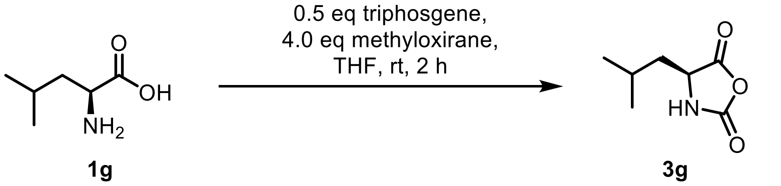

1g
1.0 g

3g
718 mg, yield = 60%

To a pressure vessel with heavy wall, L-leucine **1g** (1.0 g, 7.7 mmol, 1.0 eq), THF (20 mL), methyloxirane (30 mL, 39.0 mmol, 4.0 eq) were added sequentially under magnetic stirring. Triphosgene (1.2 g, 3.9 mmol, 0.5 eq) was finally added in one portion and the vessel was sealed immediately. The reaction was stirred at room temperature for 2 h in total and cooled down to ~ 4 °C in an ice bathe. **For safety reason, the excessive triphosgene** was quenched by adding 10 mL **cold water** at ~ 4 °C with 1-3 min stirring. The mixture was extracted with ethyl acetate (EA, 20 mL × 2) at room temperature. The combined organic phase was washed with brine and dried with anhydrous Na_2SO_4. After the removal of solvent by rotatory evaporation *in vacuum under 45 °C*, the crude product was purified by crystallization in hexane/THF **below 10 °C (preferably in a cold room)** without N_2 protection. The pure product **3g** was obtained as a white needle crystal (718 mg, yield = 60%). The NCA was stored at -10 °C for 2 months.

^{1}H NMR (400 MHz, DMSO-d_6) δ 9.13 (br, 1H), 4.45 (dd, J = 8.7, 5.5 Hz, 1H), 1.81 – 1.65 (m, 1H), 1.65 – 1.49 (m, 2H), 0.92 – 0.85 (m, 6H).
^{13}C NMR (101 MHz, DMSO-d_6) δ 172.1, 152.0, 55.6, 40.1, 24.2, 22.8, 21.2.
HRMS (ESI−FTICR, *m/z*): $[M + Na]^+$ calculated for $C_7H_{11}NNaO_3^+$: 180.0631; found: 180.0632.
FT-IR (cm^{-1}) 1828, 1787.

3h: L-Tryptophan NCA
Air humidity: 43%

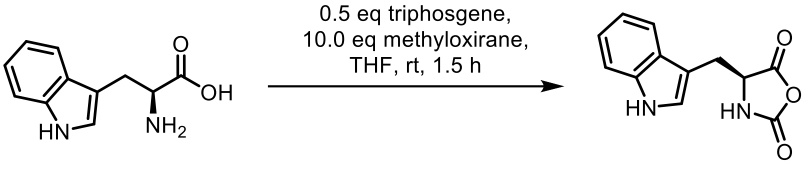

1h 1.0 g — **3h** 1.0 g, yield = 90%

To a pressure vessel with heavy wall, L-tryptophan **1h** (1.0 g, 4.9 mmol, 1.0 eq), THF (15 mL), methyloxirane (3.4 mL, 49 mmol, 10.0 eq) were added sequentially under magnetic stirring. The triphosgene (749 mg, 2.5 mmol, 0.5 eq) was finally added in one portion and the vessel was sealed immediately. The reaction was stirred at room temperature for 1.5 h in total and cooled down to ~ 4 °C in an ice bathe. **For safety reason, excessive triphosgene** was quenched by adding 15 mL **cold water** at ~ 4 °C with 1-3 min stirring. The mixture was extracted with ethyl acetate (EA, 20 mL × 2) at room temperature. The combined organic phase was washed with brine and dried with anhydrous Na_2SO_4. After the removal of solvent by rotatory evaporation *in vacuum* ***under 35 °C***, the crude product was purified by crystallization in hexane/THF **below 10 °C (preferably in a cold room)** without N_2 protection. The pure product **3h** was obtained as a crystal (1.0 g, yield = 90%). The NCA was stored at -10 °C for 2 months.

^{1}H NMR (400 MHz, DMSO-d_6) δ 11.00 (d, J = 2.5 Hz, 1H), 9.09 (s, 1H), 7.55 (d, J = 7.9 Hz, 1H), 7.36 (d, J = 8.1 Hz, 1H), 7.15 (d, J = 2.5 Hz, 1H), 7.13 – 7.05 (m, 1H), 7.05 – 6.96 (m, 1H), 4.78 (t, J = 5.0 Hz, 1H), 3.22 (dd, J = 15.0, 5.0 Hz, 1H), 3.14 (dd, J = 15.0, 5.0 Hz, 1H).
^{13}C NMR (101 MHz, DMSO-d_6) δ 171.3, 151.9, 136.0, 127.2, 124.5, 121.1, 118.6, 118.5, 111.5, 107.1, 58.3, 26.5.
HRMS (ESI−FTICR, *m/z*): $[M + H]^+$ calculated for $C_{12}H_{11}N_2O_3^+$: 231.0764; found: 231.0763.
FT-IR (cm^{-1}) 3416, 1854, 1775.

3i: L-Tyrosine NCA
Air humidity: 63%

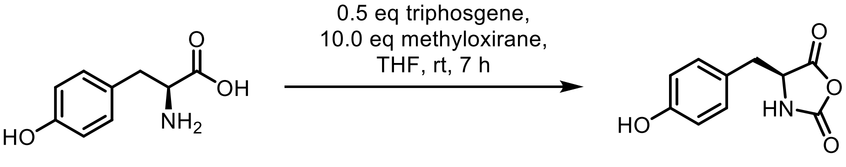

1i 1.0 g — **3i** 961 mg, yield = 83%

To a pressure vessel with heavy wall, L-tyrosine **1i** (1.0 g, 6.1 mmol, 1.0 eq), THF (15 mL), methyloxirane (4.3 mL, 61 mmol, 10.0 eq) were added sequentially under magnetic stirring.

Triphosgene (897 mg, 3.0 mmol, 0.5 eq) was finally added in one portion and the vessel was sealed immediately. The reaction was stirred at room temperature for 7 h in total and cooled down to ~ 4 °C in an ice bathe. **For safety reason, the excessive triphosgene** was quenched by adding 10 mL **cold water** at ~ 4 °C with 1-3 min stirring. The mixture was extracted with ethyl acetate (EA, 20 mL × 2) at room temperature. The combined organic phase was washed with brine and dried with anhydrous Na_2SO_4. After the removal of solvent by rotatory evaporation *in vacuum under 45 °C*, the crude product was purified by crystallization in hexane/THF **below 10 °C (preferably in a cold room)** without N_2 protection. The pure product **3i** was obtained as a white needle crystal (961 mg, yield = 83%). The NCA was stored at -10 °C for 2 months.

^{1}H NMR (400 MHz, DMSO-*d*$_6$) δ 9.34 (br, 1H), 9.03 (br, 1H), 6.97 (d, *J* = 8.4 Hz, 2H), 6.69 (d, *J* = 8.4 Hz, 2H), 4.70 (td, *J* = 5.0, 1.0 Hz, 1H), 2.93 (dd, *J* = 14.5, 5.0 Hz, 1H), 2.89 (dd, *J* = 14.5, 5.0 Hz, 1H).
^{13}C NMR (101 MHz, DMSO-*d*$_6$) δ 170.9, 156.5, 151.7, 130.7, 124.6, 115.2, 58.5, 35.5.
HRMS (ESI−FTICR, *m/z*): $[M + NH_4]^+$ calculated for $C_{10}H_{13}N_2O_4^+$: 225.0870; found: 225.0869.
FT-IR (cm^{-1}) 3305, 1857, 1763.

3j: Glycine NCA
Air humidity: 78%

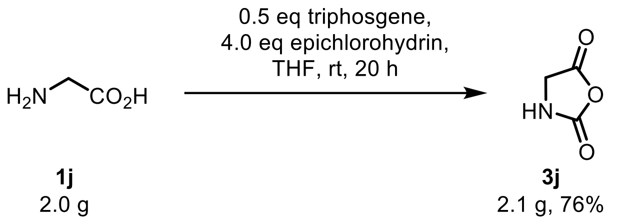

To a pressure vessel with heavy wall, glycine **1j** (2.0 g, 26.8 mmol, 1.0 eq), THF (80 mL), epichlorohydrin (8.4 mL, 107.1 mmol, 4.0 eq) were added sequentially under magnetic stirring. Triphosgene (4.0 g, 13.5 mmol, 0.5 eq) was finally added in one portion and the vessel was sealed immediately. The reaction was stirred at room temperature for 20 h in total. After the removal most of solvent by rotatory evaporation *in vacuum under 45 °C*, the crude product was purified by crystallization in PE/THF **below 10 °C (preferably in a cold room)** without N_2 protection. The pure product **3j** was obtained as a white powder (2.1 g, yield = 76%). The NCA was stored at -10 °C for 2 months.

^{1}H NMR (400 MHz, DMSO-*d*$_6$) δ 8.83 (s, 1H), 4.18 (s, 2H).
^{13}C NMR (101 MHz, DMSO-*d*$_6$) δ 169.4, 153.0, 46.3.
FT-IR (cm^{-1}) 1863, 1790.

3k: EG$_3$-GluNCA
Air humidity: 66%

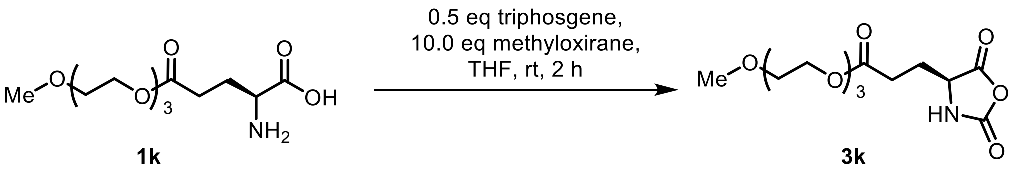

1k 2.8 g — **3k** 2.2 g, yield = 72%

To a pressure vessel with heavy wall, γ-(2-(2-(2-methoxyethoxy)ethoxy)ethyl L-glutamate **1k** (2.8 g, 9.6 mmol, 1.0 eq), THF (50 mL), methyloxirane (6.7 mL, 96.1 mmol, 10.0 eq) were added sequentially under magnetic stirring. Triphosgene (1.5 g, 4.8 mmol, 0.5 eq) was finally added in one portion and the vessel was sealed immediately. The reaction was stirred at room temperature for 2 h. **(This NCA cannot be washed with water!!!)** After the removal of solvent by rotatory evaporation *in vacuum under 50 °C*, the crude product was purified by flash column chromatography (PE/EA = 5:1 ~ EA). The pure product **3k** was obtained as a colorless oil (2.2 g, yield = 72%). The NCA was stored at -30 °C for 1 month.

^{1}H NMR (400 MHz, DMSO-d_6) δ 9.09 (br, 1H), 4.46 (ddd, *J* = 7.9, 5.4, 1.2 Hz, 1H), 4.20 – 4.07 (m, 2H), 3.60 (dd, *J* = 5.3, 4.2 Hz, 2H), 3.57 – 3.47 (m, 5H), 3.46 – 3.39 (m, 2H), 3.24 (s, 3H), 2.47 (t, *J* = 7.6 Hz, 2H), 2.10 – 1.96 (m, 1H), 1.99 – 1.83 (m, 1H).
^{13}C NMR (101 MHz, DMSO-d_6) δ 171.8, 171.4, 151.9, 71.3, 69.8, 69.7, 69.6, 68.2, 63.5, 58.1, 56.2, 29.1, 26.5.
HRMS (ESI−FTICR, *m/z*): $[M + H]^+$ calculated for $C_{13}H_{22}NO_8^+$: 320.1340; found: 320.1339.
FT-IR (cm^{-1}) 1855, 1782.

3l: L-Methionine NCA

Air humidity: 60%

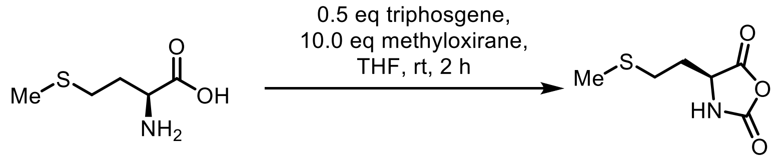

1l 1.0 g — **3l** 700 mg, yield = 60%

To a pressure vessel with heavy wall, L-methionine **1l** (1.0 g, 6.7 mmol, 1.0 eq), THF (20 mL), methyloxirane (4.7 mL, 67 mmol, 10.0 eq) were added sequentially under magnetic stirring. Triphosgene (1.0 g, 3.4 mmol, 0.5 eq) was finally added in one portion and the vessel was sealed immediately. The reaction was stirred at room temperature for 2 h in total and cooled down to ~ 4 °C in an ice bathe. **For safety reason, the excessive triphosgene** was quenched by adding 15 mL **cold water** at ~ 4 °C with 1-3 min stirring. The mixture was extracted with ethyl acetate (EA, 20 mL × 2) at room temperature. The combined organic phase was washed with brine and dried with anhydrous Na_2SO_4. After the removal of solvent by rotatory evaporation *in vacuum under 45 °C*, the crude product was purified by flash column chromatography (PE/EA = 5:1 ~ EA). The pure product **3l** was obtained as a colorless oil (700 mg, yield = 60%). The NCA was stored at -20 °C for 1 month.

^{1}H NMR (400 MHz, $CDCl_3$) δ 7.06 (br, 1H), 4.51 (ddd, *J* = 7.4, 5.0, 1.1 Hz, 1H), 2.67 (t, *J* = 6.6 Hz, 2H), 2.25 (dtd, *J* = 14.6, 6.6, 5.0 Hz, 1H), 2.17 – 2.01 (m, 1H), 2.09 (s, 3H).

^{13}C NMR (101 MHz, $CDCl_3$) δ 169.9, 152.9, 56.7, 30.2, 29.8, 15.2.
HRMS (ESI−FTICR, *m/z*): $[M + H]^+$ calculated for $C_6H_{10}NO_3S^+$: 176.0376; found: 176.0375.
FT-IR (cm^{-1}) 1854, 1787.

3m: pOEt-TyrNCA
Air humidity: 32%

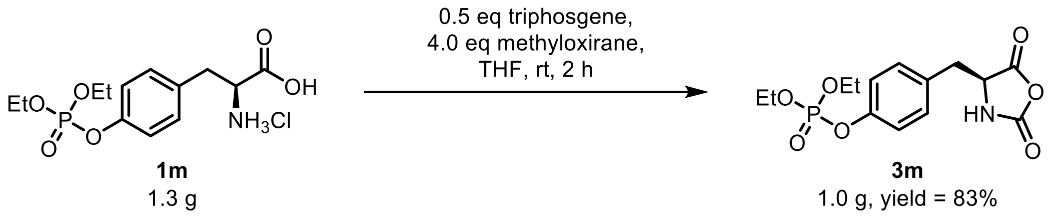

To a pressure vessel with heavy wall, *O*-diethylphospho L-tyrosine[1] **1m** (1.3 g, 3.8 mmol, 1.0 eq), THF (20 mL), methyloxirane (2.7 mL, 38.6 mmol, 10.0 eq) were added sequentially under magnetic stirring. Triphosgene (908 mg, 3.1 mmol, 0.8 eq) was finally added in one portion and the vessel was sealed immediately. The reaction was stirred at room temperature for 2 h. **(This NCA cannot be washed with water!!!)** After the removal of solvent by rotatory evaporation *in vacuum under 45 °C*, the crude product was purified by flash column chromatography (PE/EA = 5:1 ~ 1:1 ~ EA). The pure product **3m** was obtained as a yellow oil (1.0 g, yield = 83%). The NCA was stored at -20 °C.

^{1}H NMR (400 MHz, DMSO-d_6) δ 9.11 (s, 1H), 7.22 (d, J = 8.2 Hz, 2H), 7.16 (d, J = 8.2 Hz, 2H), 4.77 (t, J = 5.3 Hz, 1H), 4.19 – 4.08 (m, 4H), 3.03 (d, J = 5.3 Hz, 2H), 1.24 (t, J = 7.1 Hz, 6H).
^{13}C NMR (101 MHz, DMSO-d_6) δ 170.8, 151.7, 149.6, 149.5, 131.7, 131.2, 119.9, 119.9, 64.4, 64.3, 58.2, 35.6, 15.9, 15.9.
HRMS (ESI−FTICR, *m/z*): $[M + H]^+$ calculated for $C_{14}H_{19}NO_7P^+$: 344.0894; found: 344.0891.
FT-IR (cm^{-1}) 1857, 1790, 1740.

3n: *t*Bu-SerNCA
Air humidity: 80%

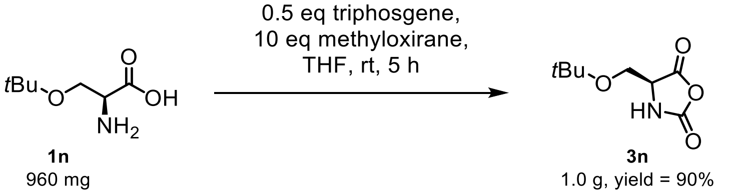

To a pressure vessel with heavy wall, *O-tert*-butyl L-serine **1n** (960 mg, 6.0 mmol, 1.0 eq), THF (50 mL), methyloxirane (4.2 mL, 59.6 mmol, 10.0 eq) were added sequentially under magnetic stirring. Triphosgene (930 mg, 3.1 mmol, 0.5 eq) was finally added in one portion and the vessel was sealed immediately. The reaction was stirred at room temperature for 5 h. **(This NCA cannot be washed with water!!!).** After the removal of solvent by rotatory evaporation *in vacuum* ***under 35 °C***, the crude product was purified by flash column chromatography (PE/EA = 5:1 ~ EA). The pure product **3n** was obtained as a colorless oil (1.0 g, yield = 90%). The NCA was stored at -20 °C for 1 week.

^{1}H NMR (400 MHz, DMSO-d_6) δ 8.95 (br, 1H), 4.57 (dt, J = 2.8, 1.5 Hz, 1H), 3.62 (dd, J = 10.3,

2.8 Hz, 1H), 3.50 (dd, J = 10.3, 2.8 Hz, 1H), 1.10 (s, 9H).
^{13}C NMR (101 MHz, DMSO-d_6) δ 170.2, 152.3, 73.2, 59.9, 58.6, 27.1.
HRMS (ESI−FTICR, *m/z*): [M + NH_4]$^+$ calculated for $C_8H_{17}N_2O_4^+$: 205.1183; found: 205.1179.
FT-IR (cm^{-1}) 1778, 1749.

3o: ε-*N*-Boc L-lysine NCA
Air humidity: 48%

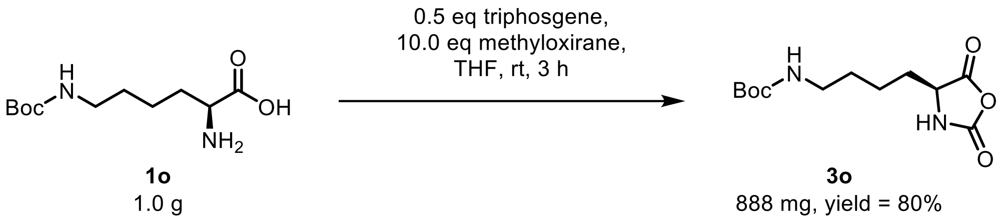

To a pressure vessel with heavy wall, ε-*N*-Boc L-lysine **1o** (1.0 g, 4.1 mmol, 1.0 eq), THF (20 mL), methyloxirane (2.8 mL, 40.6 mmol, 10.0 eq) were added sequentially under magnetic stirring. Triphosgene (620 mg, 2.1 mmol, 0.5 eq) was finally added in one portion and the vessel was sealed immediately. The reaction was stirred at room temperature for 3 h in total. **For safety reason, the excessive triphosgene** was quenched by adding 10 mL **cold water** at ~ 4 °C with 1-3 min stirring. The mixture was extracted with ethyl acetate (EA, 20 mL × 2) at room temperature. The combined organic phase was washed with brine and dried with anhydrous Na_2SO_4. After the removal of solvent by rotatory evaporation *in vacuum under 45 °C*, the crude product was purified by crystallization in hexane/THF **below 10 °C (preferably in a cold room)** without N_2 protection. The pure product **3o** was obtained as a white crystal (888 mg, yield = 80%). The NCA was stored at -10 °C for 1 month.

^{1}H NMR (400 MHz, DMSO-d_6) δ 9.07 (br, 1H), 6.79 (t, J = 6.0 Hz, 1H), 4.42 (dd, J = 7.3, 5.2 Hz, 1H), 2.89 (q, J = 6.0 Hz, 2H), 1.78 – 1.57 (m, 2H), 1.37 (s, 9H), 1.43 – 1.19 (m, 4H).
^{13}C NMR (101 MHz, DMSO-d_6) δ 171.7, 155.6, 152.0, 77.4, 57.0, 30.6, 28.9, 28.3, 21.6.
HRMS (EI, *m/z*): [M]$^+$ calculated for $C_{12}H_{21}N_2O_5^+$: 273.1445; found: 273.1447.
FT-IR (cm^{-1}) 1855, 1782, 1689.

3p: L-Serine NCA
Air humidity: 40%

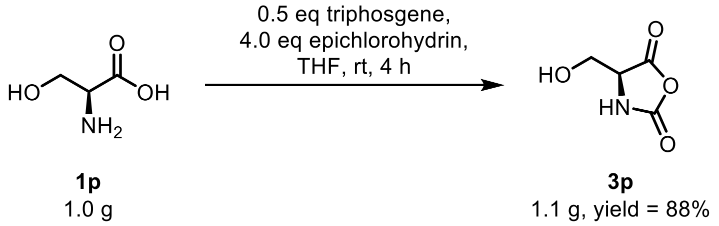

To a pressure vessel with heavy wall, L-serine **1p** (1.0 g, 9.5 mmol, 1.0 eq), THF (20 mL), epichlorohydrin (3.0 mL, 38.1 mmol, 4.0 eq) were added sequentially under magnetic stirring. Triphosgene (1.4 g, 4.8 mmol, 0.5 eq) was finally added in one portion and the vessel was sealed immediately. The reaction was stirred at room temperature for 4 h. **(This NCA cannot be washed with water!!!)** After filtration and the removal of solvent by rotatory evaporation *in vacuum under 45 °C*, the crude product was purified by crystallization in hexane/THF **below 10 °C (preferably in**

a cold room) without N_2 protection. The pure product **3p** was obtained as a colorless crystal (1.1 g, yield = 88%). The NCA was stored at -20 °C for 1 month.

^{1}H NMR (400 MHz, THF-d_8) δ 7.88 (br, 1H), 4.65 (dd, J = 6.4, 4.8 Hz, 1H), 4.32 (t, J = 3.3 Hz, 1H), 3.81 (ddd, J = 11.5, 6.4, 3.3 Hz, 1H), 3.70 (ddd, J = 11.5, 4.8, 3.3 Hz, 1H)..
^{13}C NMR (101 MHz, THF) δ 170.6, 153.6, 61.6, 61.5.
FT-IR (cm^{-1}) 3480, 1851, 1772.

3q: L-Threonine NCA

Air humidity: 58%

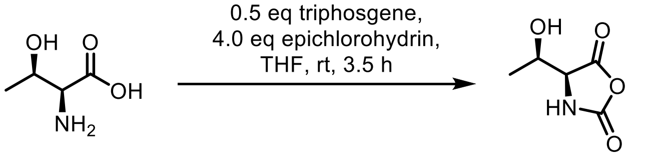

1q 1.0 g — **3q** 558 mg, yield = 46%

To a pressure vessel with heavy wall, L-threonine **1q** (1.0 g, 8.4 mmol, 1.0 eq), THF (20 mL), epichlorohydrin (2.6 mL, 33.6 mmol, 4.0 eq) were added sequentially under magnetic stirring. Triphosgene (1.3 g, 4.2 mmol, 0.5 eq) was finally added in one portion and the vessel was sealed immediately. The reaction was stirred at room temperature for 5 h in total. After filtration and the removal of solvent by rotatory evaporation *in vacuum under 45 °C*, the crude product was purified by crystallization in hexane/THF **below 10 °C (preferably in a cold room)** without N_2 protection. The pure product **3q** was obtained as a colorless crystal (558 mg, yield = 46%). The NCA was stored at -20 °C for 1 month.

^{1}H NMR (400 MHz, DMSO-d_6) δ 9.05 (br, 1H), 5.17 (br, 1H), 4.32 (dd, J = 2.3, 1.1 Hz, 1H), 3.98 (qd, J = 6.6, 2.3 Hz, 1H), 1.14 (d, J = 6.6 Hz, 3H).
^{13}C NMR (101 MHz, DMSO-d_6) δ 170.5, 152.7, 65.4, 63.7, 19.9.
HRMS (ESI−FTICR, *m/z*): $[M + H]^+$ calculated for $C_5H_8NO_4^+$: 146.0448; found: 146.0446.
FT-IR (cm^{-1}) 3337, 1846, 1762.

3s: D-Penicillamine NCA

Air humidity: 68%

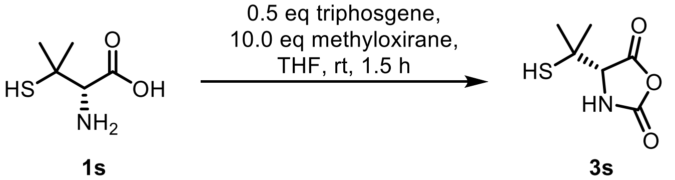

1s 1.0 g — **3s** 771 mg, yield = 53%

To a pressure vessel with heavy wall, D-penicillamine **1s** (1.0 g, 6.7 mmol, 1.0 eq), THF (20 mL), methyloxirane (5.8 mL, 82.5 mmol, 10.0 eq) were added sequentially under magnetic stirring. Triphosgene (1.2 g, 4.1 mmol, 0.5 eq) was finally added in one portion and the vessel was sealed immediately. The reaction was stirred at room temperature for 1.5 h. After the removal of solvent

by rotatory evaporation *in vacuum under 45 °C*, the crude product was purified by crystallization in PE/THF **below 10 °C (preferably in a cold room)** without N_2 protection. The pure product **3s** was obtained as a white crystal (771 mg, yield = 53%). The NCA was stored at -10 °C for 2 months.

^{1}H NMR (400 MHz, CD_2Cl_2) δ 6.49 (br, 1H), 4.28 (d, J = 1.1 Hz, 1H), 2.10 (s, 1H), 1.55 (s, 3H), 1.49 (s, 3H).
^{13}C NMR (101 MHz, CD_2Cl_2) δ 166.8, 152.2, 68.4, 46.1, 29.9, 26.3.
HRMS (ESI−FTICR, *m/z*): $[M + H]^+$ calculated for $C_6H_{10}NO_3S^+$: 176.0376; found: 176.0378
FT-IR (cm^{-1}) 3188, 1857, 1787.

Preparation of poly-L-glutamic acid:

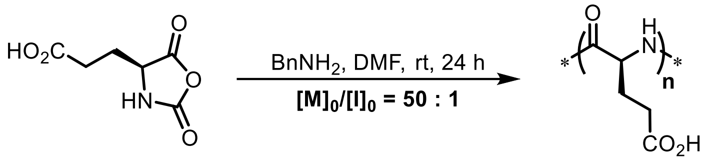

To the solution of L-glutamic acid NCA (250 mg, 1.4 mmol, 50 eq) in anhydrous DMF (500 μL), the initiator benzyl amine (29 μL × 1.0 M, 1.0 eq) was added. The reaction was stirred at room temperature for 16 h. After extensive dialysis and lyophilization, the polymer was obtained as a white powder. The experiments were independently repeated twice. The obtained M_n was measured as 8.2 ± 0.2 kg/mol, and $Đ$ was in the range of 1.04-1.06 by aqueous SEC.

4a: Sarcosine NCA
Air humidity: 44%

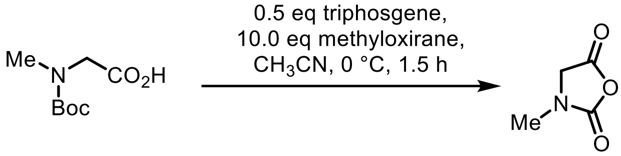

2a
1.0 g

4a
482 mg, yield = 79%

To a 100 mL flask, Boc-sarcosine **2a** (1.0 g, 5.3 mmol, 1.0 eq), CH_3CN (20 mL), methyloxirane (3.7 mL, 53 mmol, 10.0 eq) were added sequentially under magnetic stirring in an ice bathe. Triphosgene (785 mg, 2.7 mmol, 0.5 eq) was finally added in one portion and the flask was kept half-open to facilitate the in situ deprotection of Boc. The reaction was stirred at ~ 4 °C in an ice bathe for 1.5 h. **For safety reason, the excessive triphosgene** was quenched by adding 10 mL **cold water** at ~ 4 °C with 1-3 min stirring. The mixture was extracted with ethyl acetate (EA, 20 mL × 2) at room temperature. The combined organic phase was washed with brine and dried with anhydrous Na_2SO_4. After the removal of solvent by rotatory evaporation *in vacuum under 45 °C*, the crude product was purified by crystallization in hexane/THF **below 10 °C (preferably in a cold room)** without N_2 protection. The pure product **4a** was obtained as a white granular crystal (482 mg, yield = 79%). The NCA was stored at -20 °C for 1 month.

^{1}H NMR (400 MHz, $CDCl_3$) δ 4.13 (s, 2H), 3.03 (s, 3H).

^{13}**C NMR** (101 MHz, $CDCl_3$) δ 165.5, 152.4, 51.0, 30.4.
FT-IR (cm^{-1}) 1849, 1765.

7b: L-Mandelic acid OCA
Air humidity: 65%

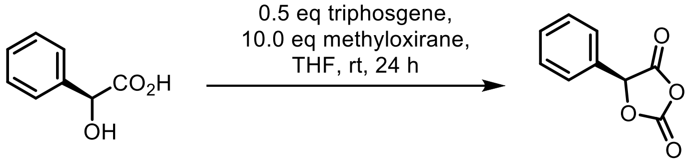

5b 1.0 g — **7b** 575 mg, yield = 49%

To a pressure vessel with heavy wall, L-mandelic acid **5b** (1.0 g, 6.6 mmol, 1.0 eq), THF (20 mL), methyloxirane (4.6 mL, 66 mmol, 10.0 eq) were added sequentially under magnetic stirring. Triphosgene (976 mg, 3.3 mmol, 0.5 eq) was finally added in one portion and the vessel was sealed immediately. The reaction was stirred at room temperature for 24 h in total. After the removal of solvent by rotatory evaporation *in vacuum under 45 °C*, the crude product was purified by crystallization in hexane/THF **below 10 °C (preferably in a cold room)** without N_2 protection. The pure product **7b** was obtained as a white needle crystal (575 mg, yield = 49%). The NCA was stored at -10 °C for 2 month.

^{1}**H NMR** (400 MHz, DMSO-d_6) δ 7.47 – 7.40 (m, 2H), 7.39 – 7.24 (m, 3H), 5.04 (s, 1H).
^{13}**C NMR** (101 MHz, DMSO-d_6) δ 174.2, 140.3, 128.2, 127.8, 126.7, 72.5.
HRMS (EI, *m/z*): $[M]^+$ calculated for $C_9H_6O_4^+$: 178.02606; found: 178.02615.
FT-IR (cm^{-1}) 3447, 1727.

8a: β-alanine NCA
Air humidity: 45%

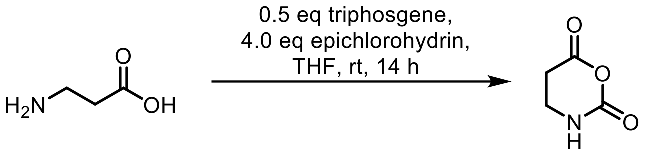

6a 1.0 g — **8a** 879 mg, yield = 68%

To a pressure vessel with heavy wall, β-alanine **6a** (1.0 g, 11.3 mmol, 1.0 eq), THF (40 mL), epichlorohydrin (3.5 mL, 45.1 mmol, 4.0 eq) were added sequentially under magnetic stirring. Triphosgene (1.7 g, 5.7 mmol, 0.5 eq) was finally added in one portion and the vessel was sealed immediately. The reaction was stirred at room temperature for 14 h and filtered directly. After the removal of solvent by rotatory evaporation *in vacuum under 40 °C*, the crude product was purified by crystallization in PE/THF **below 10 °C (preferably in a cold room)** without N_2 protection. The pure product **8a** was obtained as a white crystal (879 mg, yield = 68%). The NCA was stored at -20 °C for 1 month.

^{1}**H NMR** (400 MHz, DMSO-d_6) δ 8.38 (br, 1H), 3.26 (td, J = 6.7, 2.8 Hz, 2H), 2.75 (t, J = 6.7 Hz, 2H).

^{13}C NMR (101 MHz, DMSO-d_6) δ 167.0, 149.6, 34.3, 28.3.
FT-IR (cm^{-1}) 1801, 1763.

8b: β-aminobutyric acid NCA
Air humidity: 45%

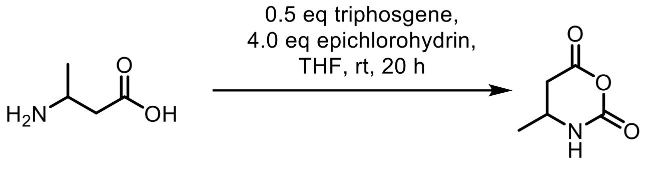

6b 1.0 g — **8b** 1.1 g, yield = 86%

To a pressure vessel with heavy wall, β-aminobutyric acid **6b** (1.0 g, 9.8 mmol, 1.0 eq), THF (30 mL), epichlorohydrin (3.0 mL, 39.2 mmol, 4.0 eq) were added sequentially under magnetic stirring. Triphosgene (1.4 g, 4.9 mmol, 0.5 eq) was finally added in one portion and the vessel was sealed immediately. The reaction was stirred at room temperature for 20 h and filtered directly. After the removal of solvent by rotatory evaporation *in vacuum under 45 °C*, the crude product was purified by crystallization in PE/THF **below 10 °C (preferably in a cold room)** without N_2 protection. The pure product **8b** was obtained as a white crystal (1.1 g, yield = 86%). The NCA was stored at -20 °C for 1 month.

^{1}H NMR (400 MHz, DMSO-d_6) δ 8.46 (s, 1H), 3.71 – 3.59 (m, 1H), 2.81 (dd, J = 15.9, 4.0 Hz, 1H), 2.58 (dd, J = 15.9, 9.2 Hz, 1H), 1.11 (d, J = 6.5 Hz, 3H).
^{13}C NMR (101 MHz, DMSO-d_6) δ 166.7, 149.1, 41.9, 35.5, 20.2.
HRMS (EI, *m/z*): $[M+H]^+$ calculated for $C_5H_8NO_3^+$: 130.0499; found: 130.0499.
FT-IR (cm^{-1}) 1804, 1760.

8d: L-aspartic acid β-benzyl ester NCA
Air humidity: 60%

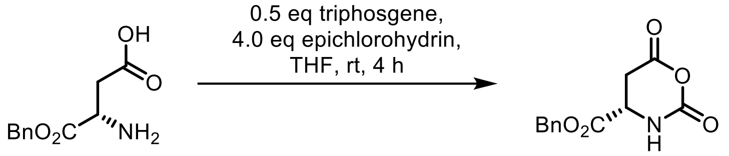

6d 1.0 g — **8d** 1.1 g, yield = 96%

To a pressure vessel with heavy wall, L-aspartic acid β-methyl ester **6d** (1.0 g, 4.6 mmol, 1.0 eq), THF (30 mL), epichlorohydrin (1.4 mL, 17.9 mmol, 4.0 eq) were added sequentially under magnetic stirring. Triphosgene (675 mg, 2.3 mmol, 0.5 eq) was finally added in one portion and the vessel was sealed immediately. The reaction was stirred at room temperature for 4 h. After the removal of solvent by rotatory evaporation *in vacuum under 45 °C*, the crude product was purified by crystallization in PE/THF **below 10 °C (preferably in a cold room)** without N_2 protection. The pure product **8d** was obtained as a white crystal (1.1 g, yield = 96%). The NCA was stored at -20 °C for 2 months.

^{1}H NMR (400 MHz, DMSO-d_6) δ 9.06 – 8.99 (br, 1H), 7.38 (s, 6H), 5.19 (s, 2H), 4.52 – 4.20 (m, 1H), 3.26 (dd, J = 16.8, 7.1 Hz, 1H), 2.94 (d, J = 16.8 Hz, 1H).
^{13}C NMR (101 MHz, DMSO-d_6) δ 170.3, 165.1, 148.8, 135.3, 128.6, 128.4, 128.1, 67.1, 48.3, 30.6.
HRMS (EI, *m/z*): $[M+H]^+$ calculated for $C_{12}H_{12}NO_5^+$: 250.0710; found: 250.0708.
FT-IR (cm^{-1}) 1808, 1764.

8e: 3-amino-3-(p-tolyl)propanoic acid NCA
Air humidity: 70%

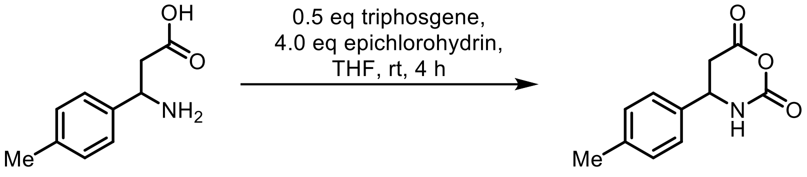

6e
1.0 g

8e
1.0 g, yield = 87%

To a pressure vessel with heavy wall, 3-amino-3-(*p*-tolyl)propanoic acid **6e** (1.0 g, 5.7 mmol, 1.0 eq), THF (30 mL), epichlorohydrin (1.8 mL, 22.3 mmol, 4.0 eq) were added sequentially under magnetic stirring. Triphosgene (827 mg, 2.8 mmol, 0.5 eq) was finally added in one portion and the vessel was sealed immediately. The reaction was stirred at room temperature for 4 h and filtered directly. After the removal of solvent by rotatory evaporation *in vacuum under 45 °C*, the crude product was purified by crystallization in PE/THF **below 10 °C (preferably in a cold room)** without N_2 protection. The pure product **8e** was obtained as a white crystal (1.0 g, yield = 87%). The NCA was stored at -20 °C for 2 months.

^{1}H NMR (400 MHz, DMSO-d_6) δ 8.90 (d, J = 3.0 Hz, 1H), 7.21 (s, 4H), 4.76 (td, J = 6.2, 3.0 Hz, 1H), 3.13 (dd, J = 16.1, 6.2 Hz, 1H), 2.94 (dd, J = 16.1, 6.2 Hz, 1H), 2.29 (s, 3H).
^{13}C NMR (101 MHz, DMSO-d_6) δ 166.1, 149.3, 137.5, 136.7, 129.4, 126.0, 49.0, 36.3, 20.7.
HRMS (ESI−FTICR, *m/z*): $[M + Na]^+$ calculated for $C_{11}H_{11}NNaO_3^+$: 228.0631; found: 228.0629.
FT-IR (cm^{-1}) 1797, 1735.

8f: 3-amino-3-(4-chlorophenyl)propanoic acid NCA
Air humidity: 90%

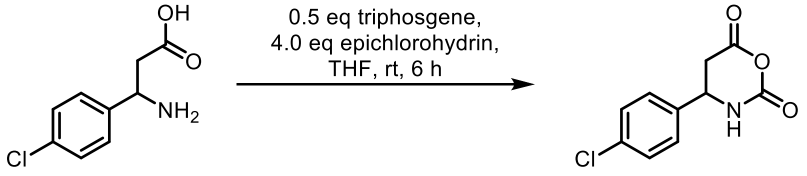

6f
1.0 g

8f
953 mg, yield = 84%

To a pressure vessel with heavy wall, 3-amino-3-(4-chlorophenyl)propanoic acid **6f** (1.0 g, 5.0 mmol, 1.0 eq), THF (30 mL), epichlorohydrin (1.6 mL, 20.4 mmol, 4.0 eq) were added sequentially under magnetic stirring. Triphosgene (756 mg, 2.6 mmol, 0.5 eq) was finally added in one portion and the vessel was sealed immediately. The reaction was stirred at room temperature for 6 h and filtered directly. After the removal of solvent by rotatory evaporation *in vacuum under 45 °C*, the crude

product was purified by crystallization in PE/THF **below 10 °C (preferably in a cold room)** without N_2 protection. The pure product **8f** was obtained as a white crystal (953 mg, yield = 84%). The NCA was stored at -20 °C for 2 months.

^{1}H NMR (400 MHz, DMSO-d_6) δ 8.95 (d, J = 2.8 Hz, 1H), 7.48 (d, J = 8.1 Hz, 2H), 7.37 (d, J = 8.1 Hz, 2H), 4.86 – 4.80 (m, 1H), 3.15 (dd, J = 16.1, 5.5 Hz, 1H), 2.98 (dd, J = 16.1, 7.2 Hz, 1H).
^{13}C NMR (101 MHz, DMSO-d_6) δ 165.8, 149.2, 138.6, 132.8, 128.8, 128.2, 48.8, 36.0.
HRMS (ESI−FTICR, *m/z*): $[M + NH_4]^+$ calculated for $C_{10}H_{12}ClN_2O_3^+$: 243.0531; found: 243.0530.
FT-IR (cm^{-1}) 1807, 1760.

Preparation of P(EG3-Glu)-*branch*-PCys:

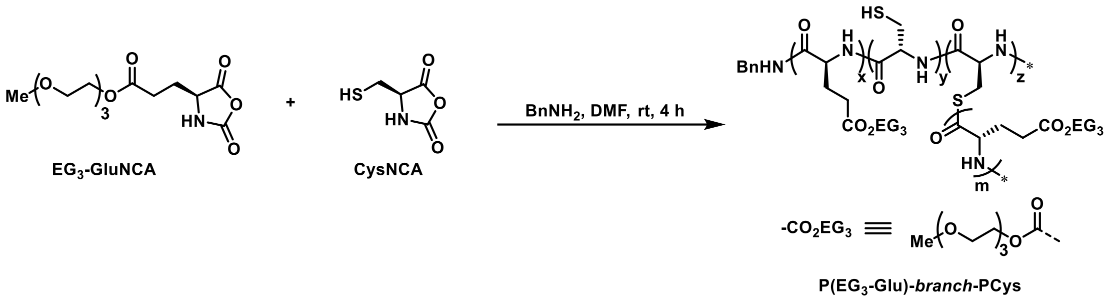

To the solution of EG3-GluNCA (1.0 g, 3.1 mmol, 5 eq) and CysNCA (75 mg, 0.6 mmol, 1 eq) in ultradry DMF (1.0 mL) under room temperature, the initiator benzyl amine (25 µL × 0.5 M, 0.02 eq) was added. The reaction was stirred at room temperature for 4 h. The polymer was precipitated in diethyl ether and centrifuged, which was further purified by extensive dialysis. After lyophilization, the polymer was obtained as a white powder (780 mg, yield = 86%).

Calibration of thiol determination with cysteine

Into 1 ml of sodium phosphate buffer (0.1 M, pH 8.0), 4.0 mg DTNB was dissolved. Then, into 50 ml of buffer, 11.8 mg (75 µmol) of cysteine hydrochloride were added and stirred for several minutes until all cysteine was dissolved. Subsequently, a set of cysteine standards by diluting cysteine hydrochloride solution at 1.5 mM, 1.25 mM, 1.0 mM, 0.75 mM, 0.5 mM, 0.25 mM were prepared. The mixture of 2 µL DTNB and 10 µL standards was diluted with 100 µL buffer and incubated at room temperature for 15 minutes. The absorbance at 412 nm was measured to generate a standard curve.

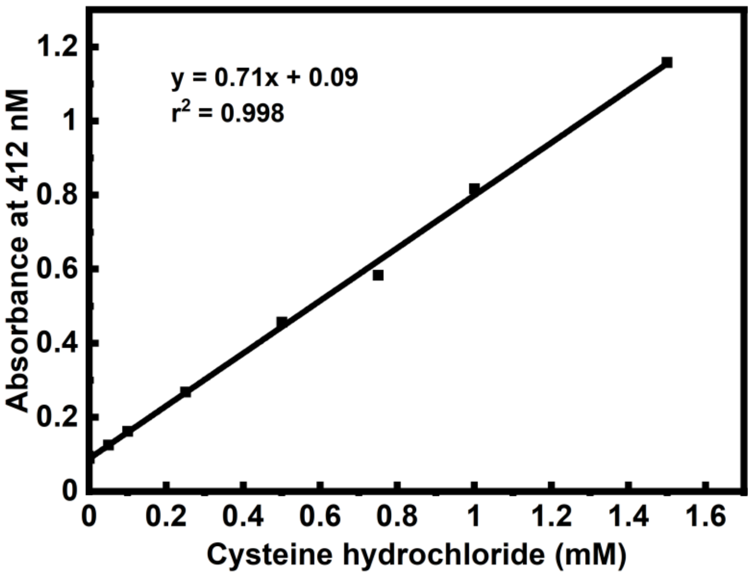

Measurement of polymer solution

Into 2 mL sodium phosphate buffer (0.1 M, pH 8.0), 2.1 mg P(EG_3-Glu)-*branch*-PCys was dissolved. The mixture of 2 μL DTNB and 10 μL polymer solution was diluted with 100 μL buffer and incubated at room temperature for 15 minutes. The absorbance at 412 nm was measured. The 0.24 mM concentration of free thiol of polymer was determined by standard curve.

Supplementary Figures and Tables

Kinetic study of Bn-GluNCA with D_2O in THF-d_8:

Bn-GluNCA (267 mg, 1.0 mmol, 1.0 eq) was dissolved in 0.5 mL THF-d_8, to which was added D_2O (20 µL, 1.1 mmol, 1.1 eq). The mixture was analyzed at different time points by ^{1}H NMR to calculate the NCA conversion.

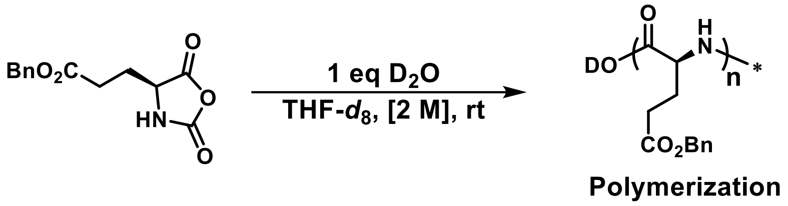

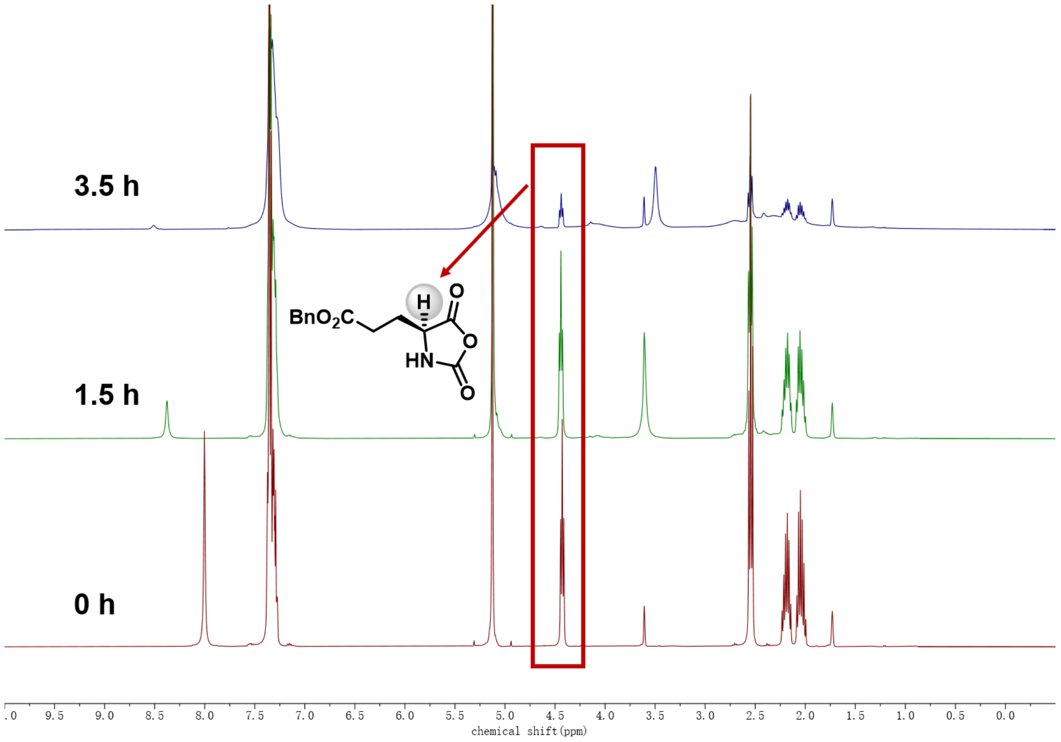

entry	time (h)	conversion (%)
1	0	0
2	1.5	13
3	3.5	80

Supplementary Figure 1 Overlay of ^{1}H NMR spectra of Bn-GluNCA with D_2O at different time points, the conversion calculation was based on the integrate of α-H of NCA using the solvent peak as the internal standard.

Kinetic study of Bn-GluNCA with D_2O and DCl in THF-d_8:

1. In situ generation of DCl and D_2O mixture in THF-d_8: To a 25 mL flask, triphosgene (395 mg, 1.3 mmol, 0.33 eq) and 2.0 mL THF-d_8 were added sequentially under magnetic stirring at 0 °C. To the mixture was added D_2O (160 µL, 8.0 mmol, 2.0 eq) and CO_2 gas was generated immediately. After stirring at 0 °C for 20 min, the DCl solution in THF-d_8 was used directly.

2. Kinetic study: To the above mixture of DCl and D_2O in THF-d_8, Bn-GluNCA (1.05 g, 4.0 mmol, 1 eq) was added at room temperature. An aliquot (400 µL) of the reaction solution was taken out at different time and analyzed immediately by ^1H NMR to calculate the NCA conversion. Note the peaks became broader over time because a large amount of amino acid precipitated in the NMR tube, which makes the shimming unsatisfactory. The precipitation was separated and confirmed by ^1H NMR as amino acid.

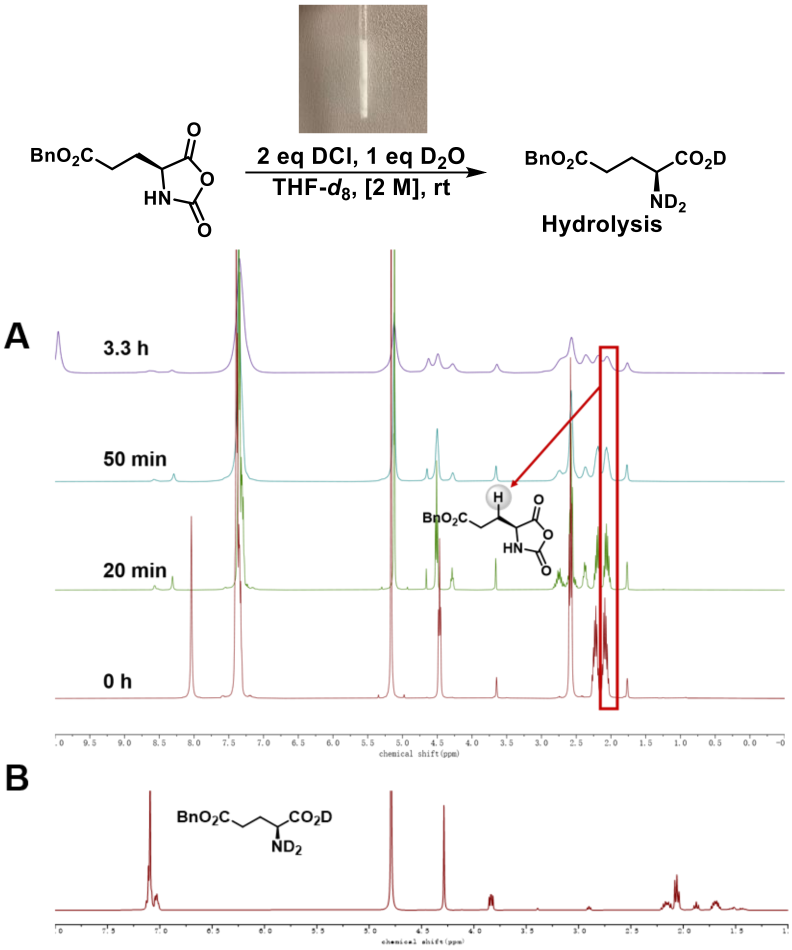

entry	time (h)	conversion (%)
1	0	0.0
2	0.33	49
3	0.83	58
4	3.3	79

Supplementary Figure 2 (A) Overlay of ^1H NMR spectra of Bn-GluNCA with D_2O and DCl at different time, the conversion calculation was based on the integrate of β-H of NCA using the solvent peak as the internal standard. (B) ^1H NMR spectra of hydrolyzed Bn-GluNCA.

Kinetic study of α-pinene with HCl in THF-d_8:

To a NMR tube, α-pinene (79 µL, 0.5 mmol, 1.0 eq) and 0.5 mL THF-d_8 were mixed and record as 0 h. Then, 12 N concentrated HCl (aq, 42 µL, 0.5 mmol, 1.0 eq) was added to the NMR tube. The mixture was analyzed at different time points by ^{1}H NMR to calculate the α-pinene conversion.

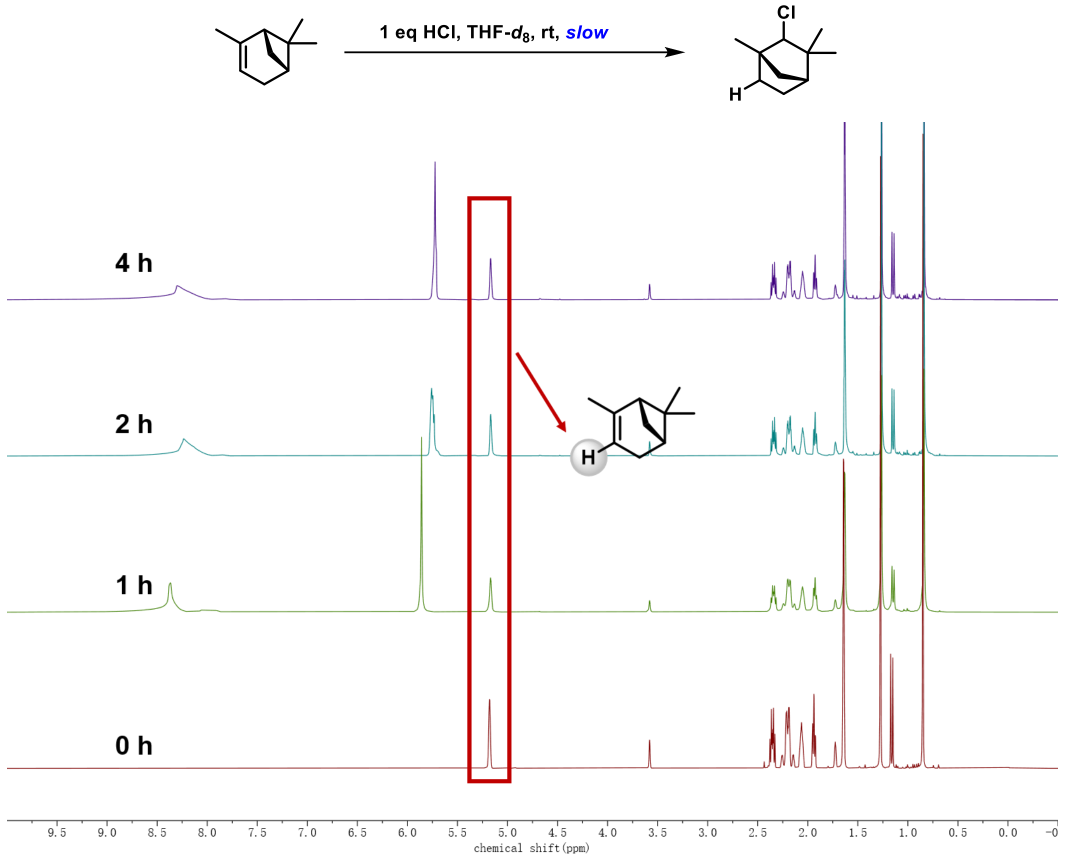

entry	time (h)	conversion (%)
1	0	0
2	1	18
3	2	20
4	4	21

Supplementary Figure 3 Overlay of ^{1}H NMR spectra of α-pinene with HCl at different time points, the conversion calculation was based on the integrate of alkenyl H using the solvent peak as the internal standard.

Kinetic study of PO with HCl in THF-d_8:

To a NMR tube, PO (35 μL, 0.5 mmol, 1 eq) and 0.5 mL THF-d_8 were mixed and recorded by NMR as 0 h. Then, 12 N concentrated HCl (aq, 42 μL, 0.5 mmol, 1 eq) was added and heat was instantly released. The mixture was analyzed immediately by ^{1}H NMR to calculate the PO conversion.

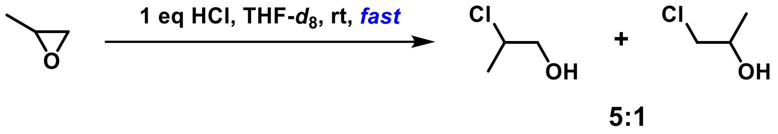

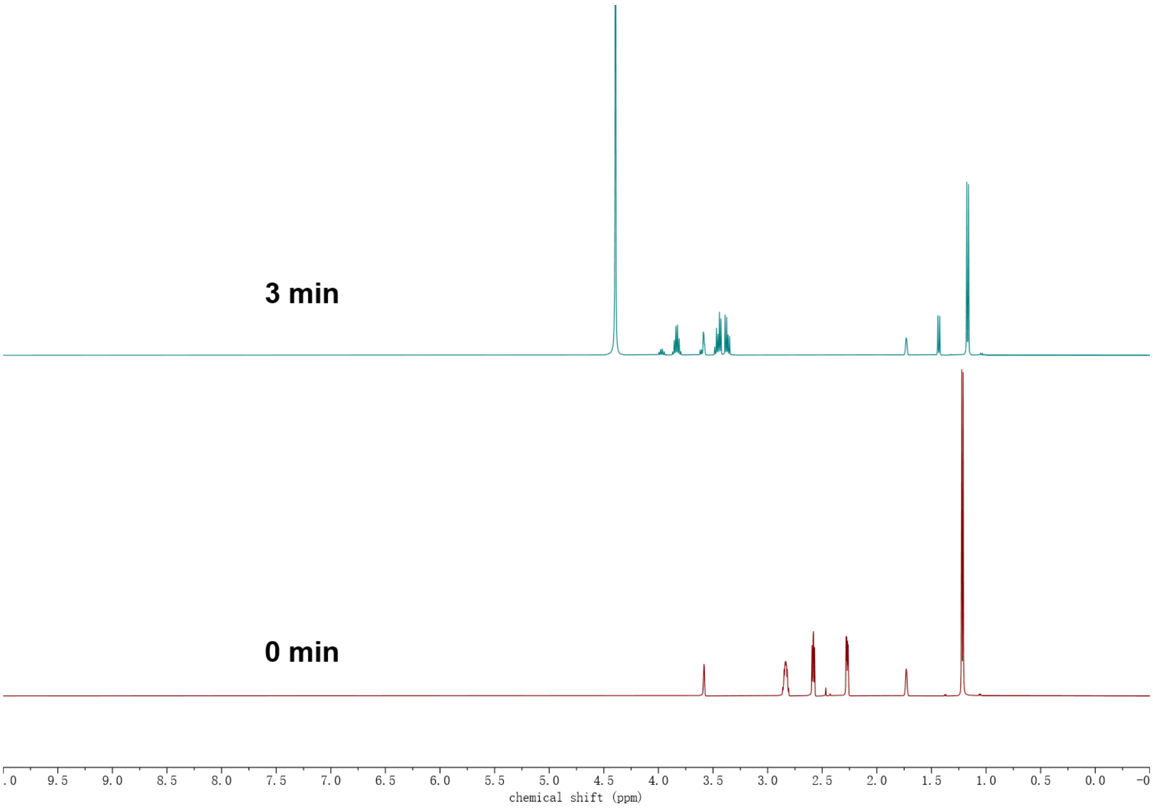

entry	time (min)	conversion (%)
1	0	0
2	3	100

Supplementary Figure 4 Overlay of ^{1}H NMR spectra of PO with HCl at different time points.

DFT Study (Supplementary Figure 5-6)

All DFT calculations were performed in Gaussian 09 E program.[2] Becke3LYP functional[3] was used to locate all the stationary points and execute frequency analysis in the THF solution phase with SMD solvation model.[4] The Pople 6-31G(d)[5] basis set was applied for all elements. The intrinsic reaction coordinate (IRC) calculations were also preformed to confirm whether the stationary points were transition states or intermediates. Single point energies were calculated using M06-2X[6] functional and the def2-TZVPP basis set[7] in THF using the SMD solvation model.

We elucidated the mechanism of phosgenation of amino acid using L-alanine as the model molecule. Two pathways were initially considered: the acid acylation (red) and the amine acylation (green). The DFT calculations revealed that the amino acylation process was favored over acid acylation process by 5.5 kcal/mol due to the stronger nucleophilicity of amine. This result indicated that the amine acylation pathway was the favorable one. Thus, the amine acylation process was conducted to investigate the epoxide effect in the ring-closing step.

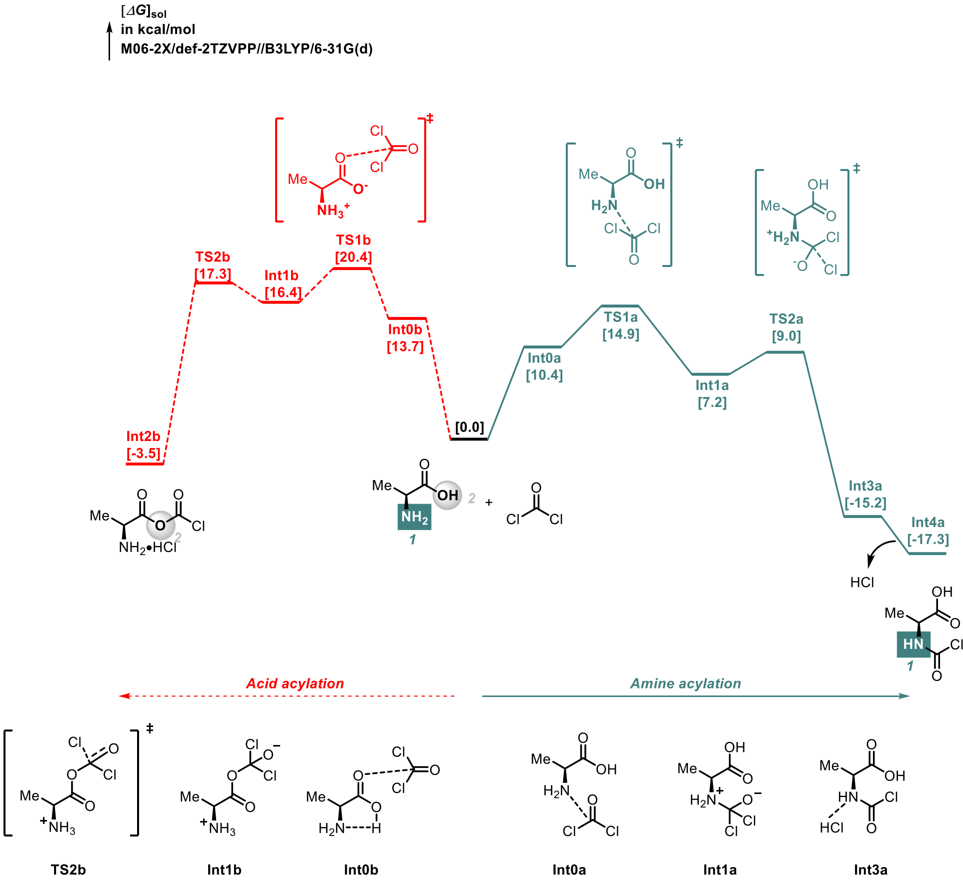

Supplementary Figure 5 DFT study of the acylation step.

Next, we investigated the effect of epoxide. The DFT calculations revealed that the rate-determining step of the whole reaction was the ring closing step from **Int4a** to **Int5c** with the activation Gibbs free energy to be 26.1 kcal/mol. The non-epoxide pathway from **Int4a** to **Int5a** was disfavored over 2.9 kcal/mol. Moreover, the IRC showed that the Cl anion could directly shift to the neighboring carbonyl group to form the **Int5c**.

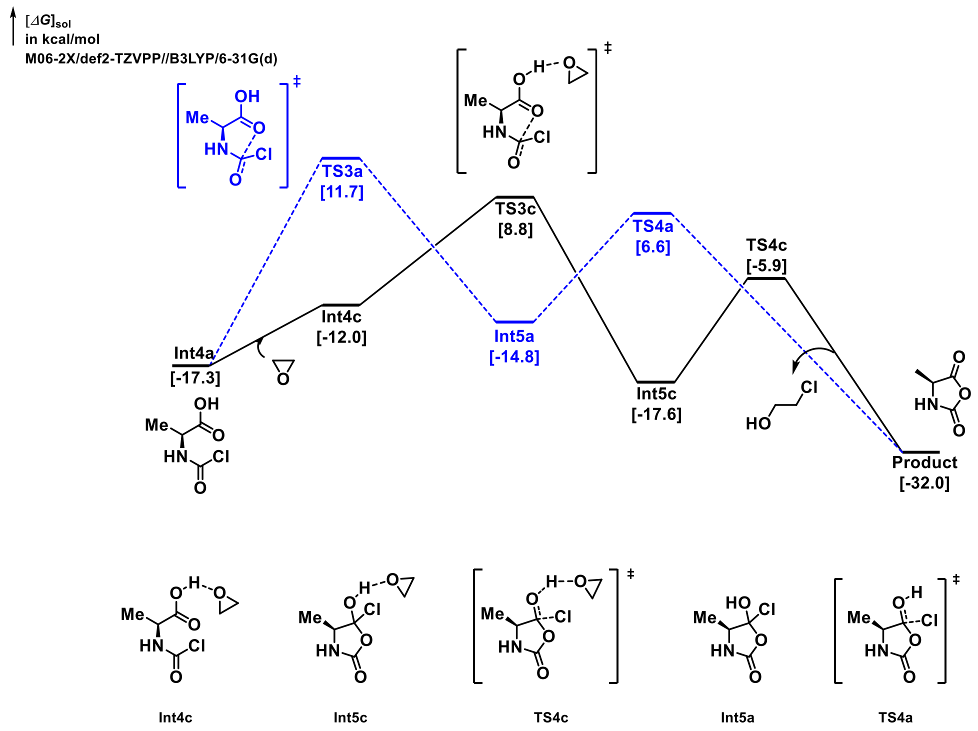

Supplementary Figure 6 DFT calculations of the effect of epoxide with model reaction.

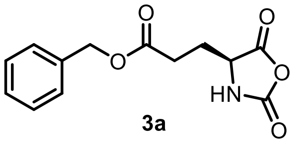

3a

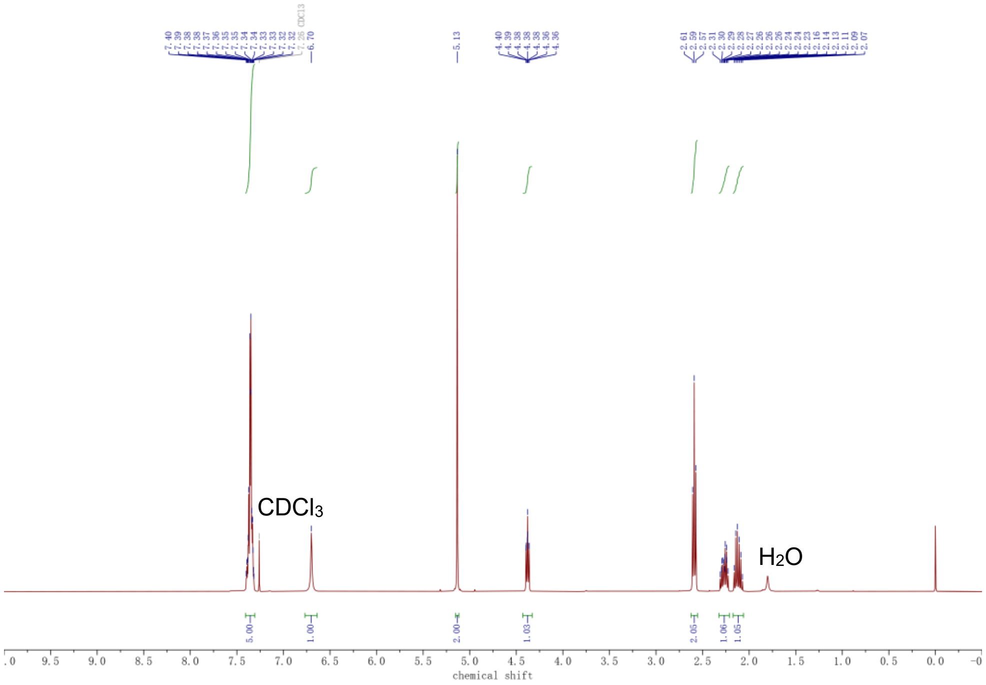

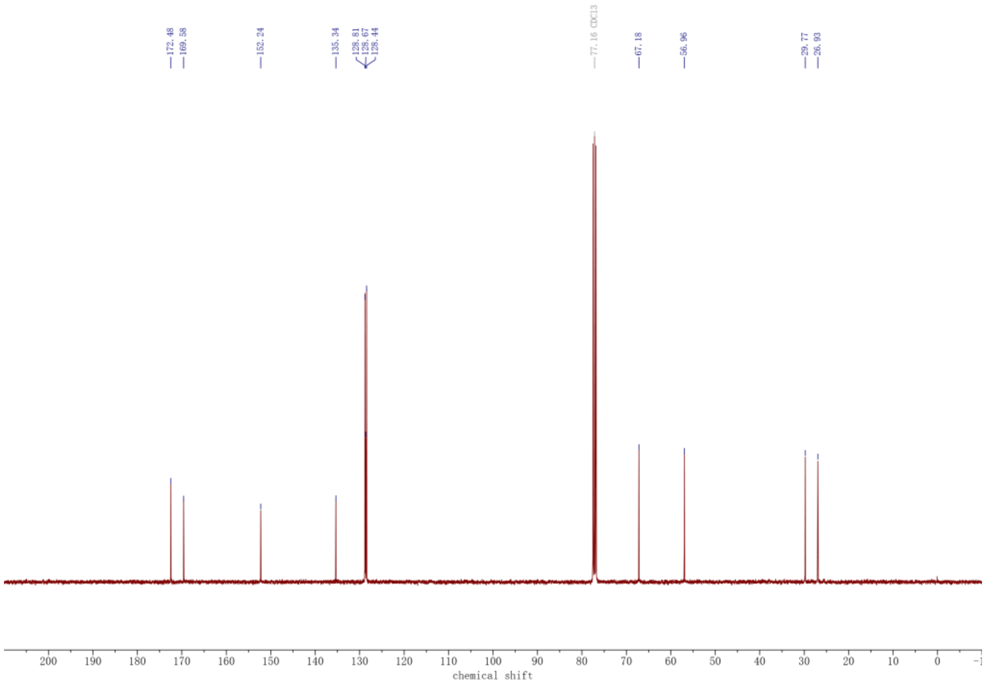

Supplementary Figure 7 ^{1}H and ^{13}C NMR spectra of 3a in $CDCl_3$.

Tests of PO Loading on NCA yield

	NCA yield	
	4 eq PO	10 eq PO
Sarcosine NCA	79%	63%
L-Phenyl lactic acid OCA	62%	65%
L-Mandelic acid OCA	36%	49%
L-Tryptophan NCA	90%	89%

Supplementary Table 1

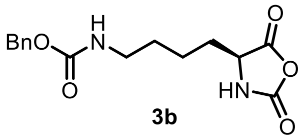

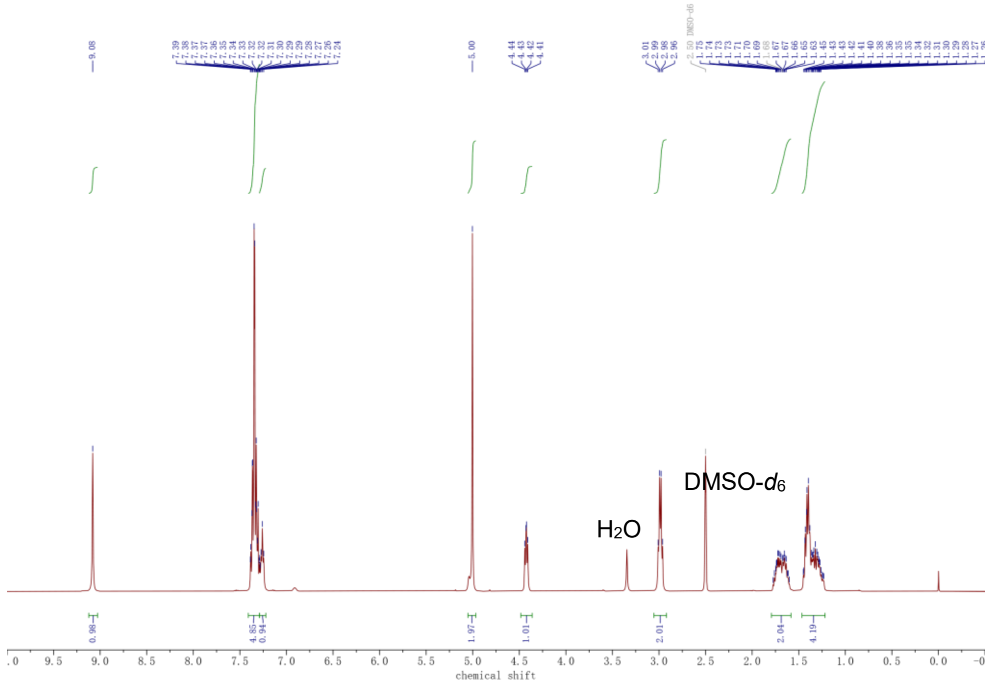

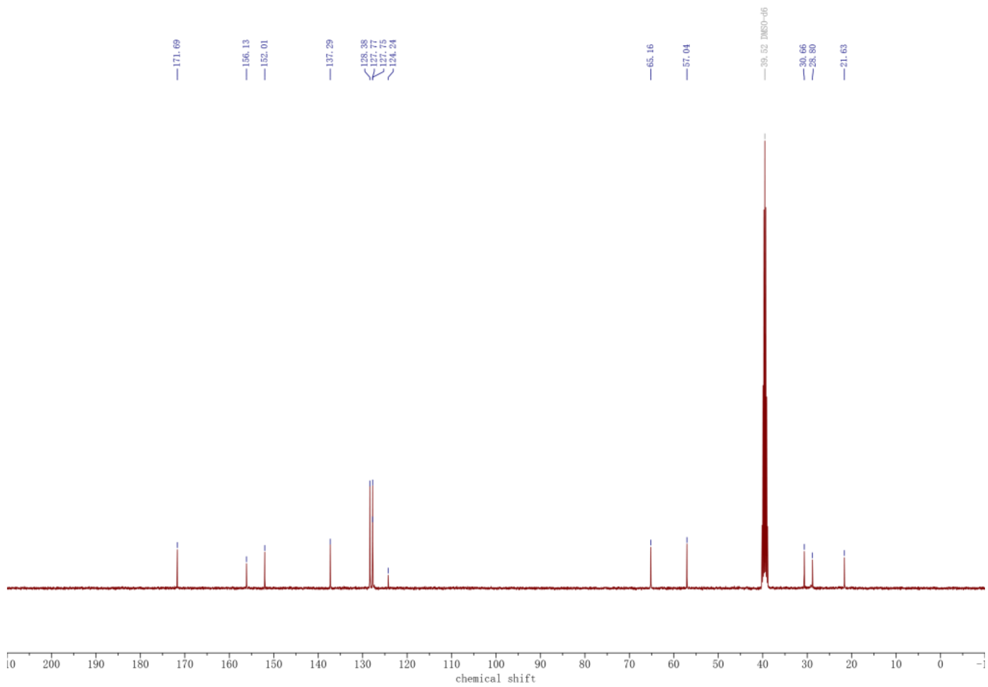

Supplementary Figure 8 ^{1}H and ^{13}C NMR spectra of 3b in DMSO-d_6

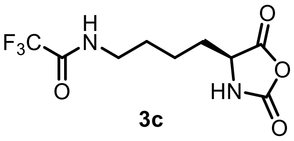

3c

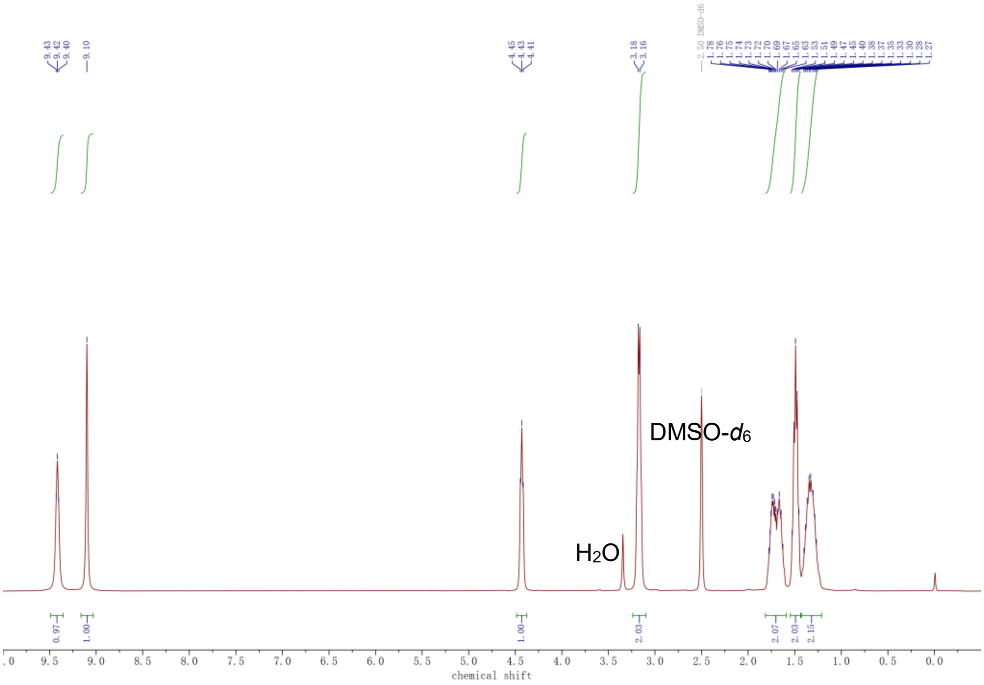

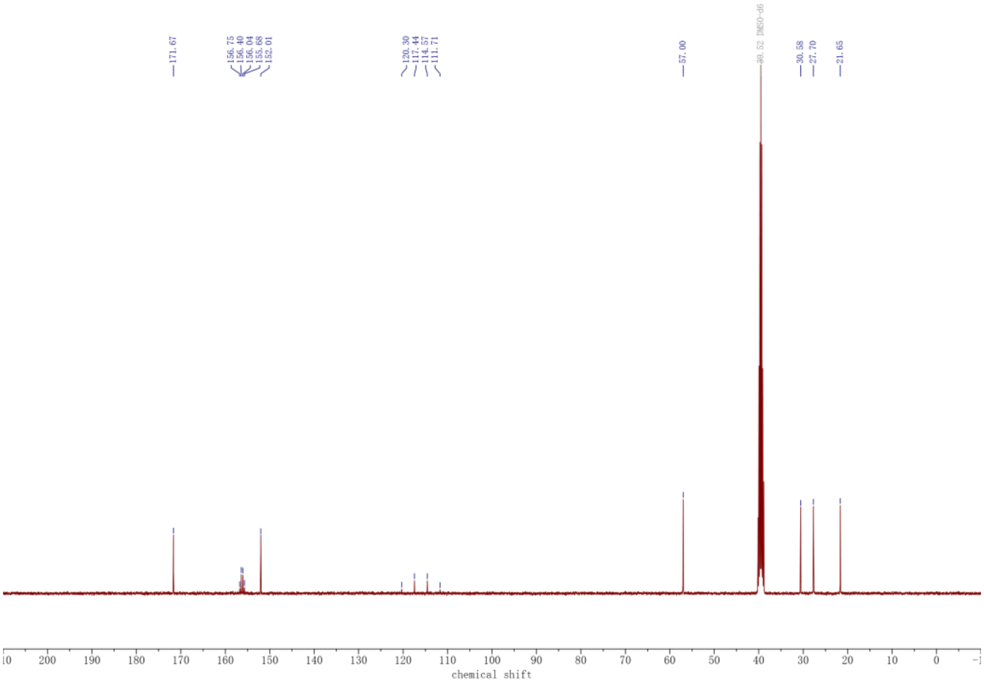

Supplementary Figure 9 ^{1}H and ^{13}C NMR spectra of 3c in DMSO-d_6.

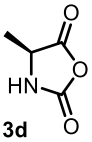

3d

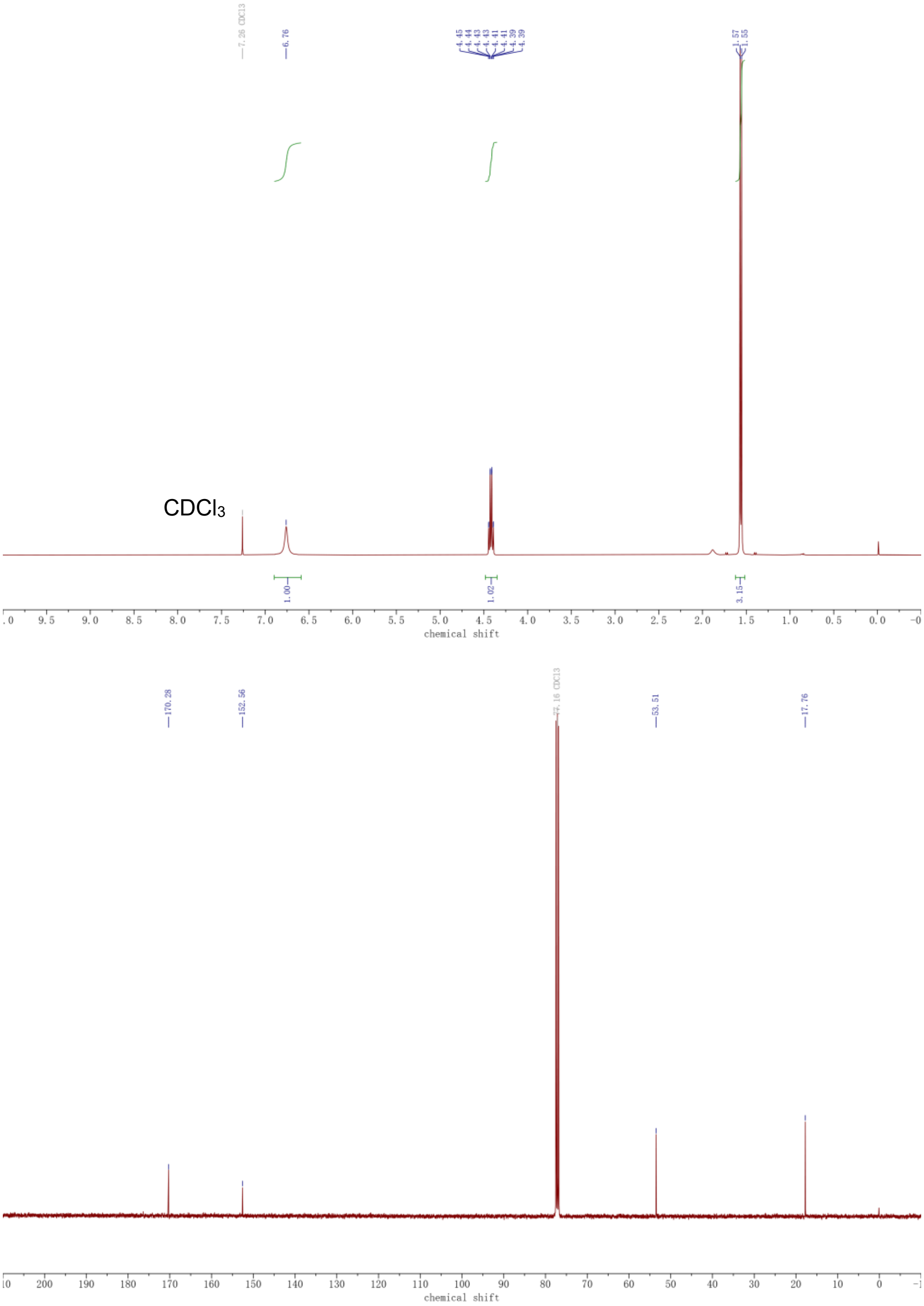

Supplementary Figure 10 ^{1}H and ^{13}C NMR spectra of 3d in $CDCl_3$.

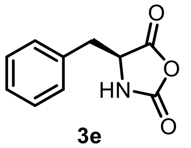

3e

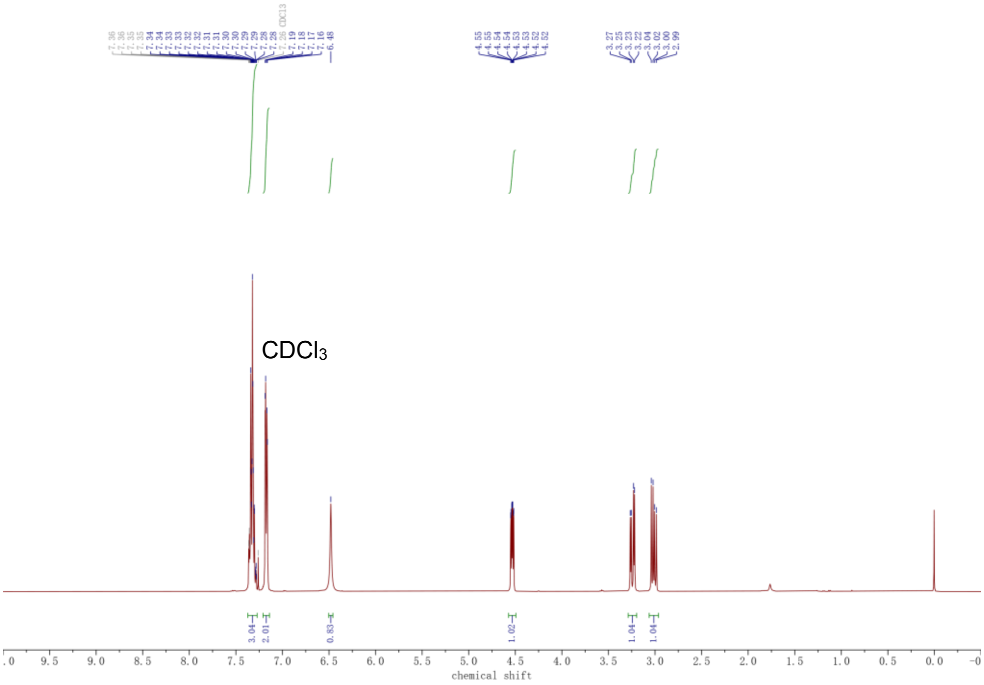

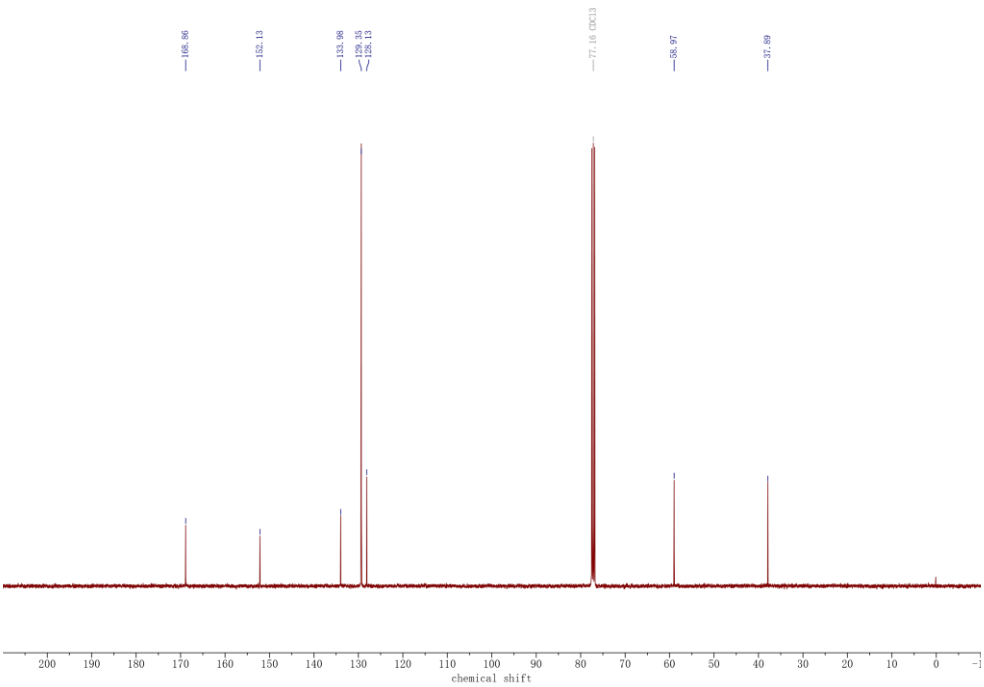

Supplementary Figure 11 ^{1}H and ^{13}C NMR spectra of 3e in $CDCl_3$.

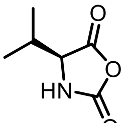

3f

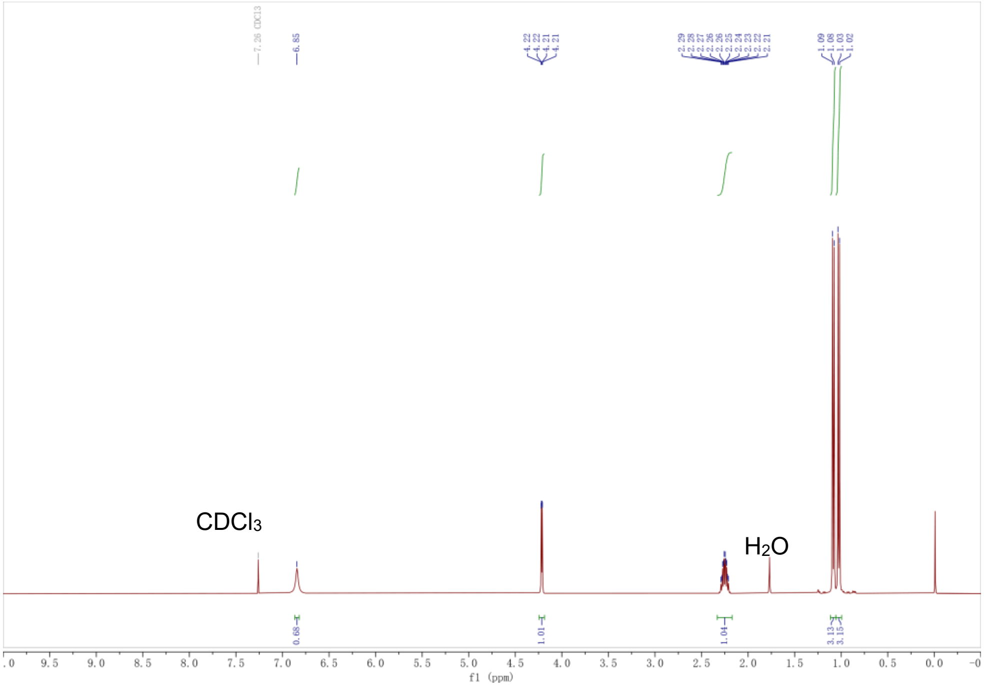

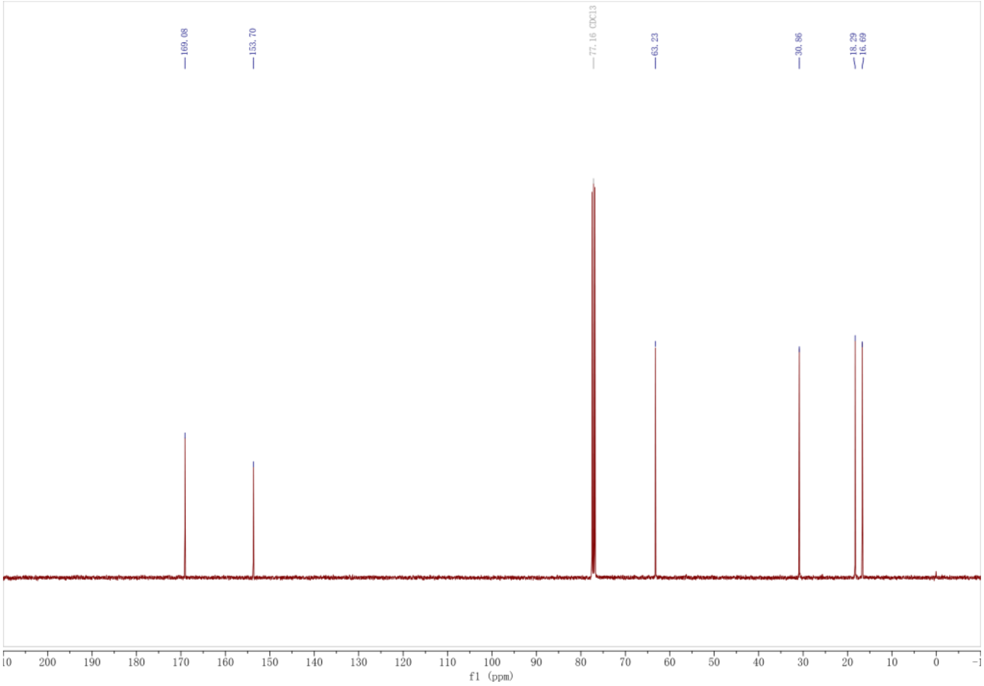

Supplementary Figure 12 ^{1}H and ^{13}C NMR spectra of 3f in $CDCl_3$.

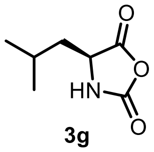

3g

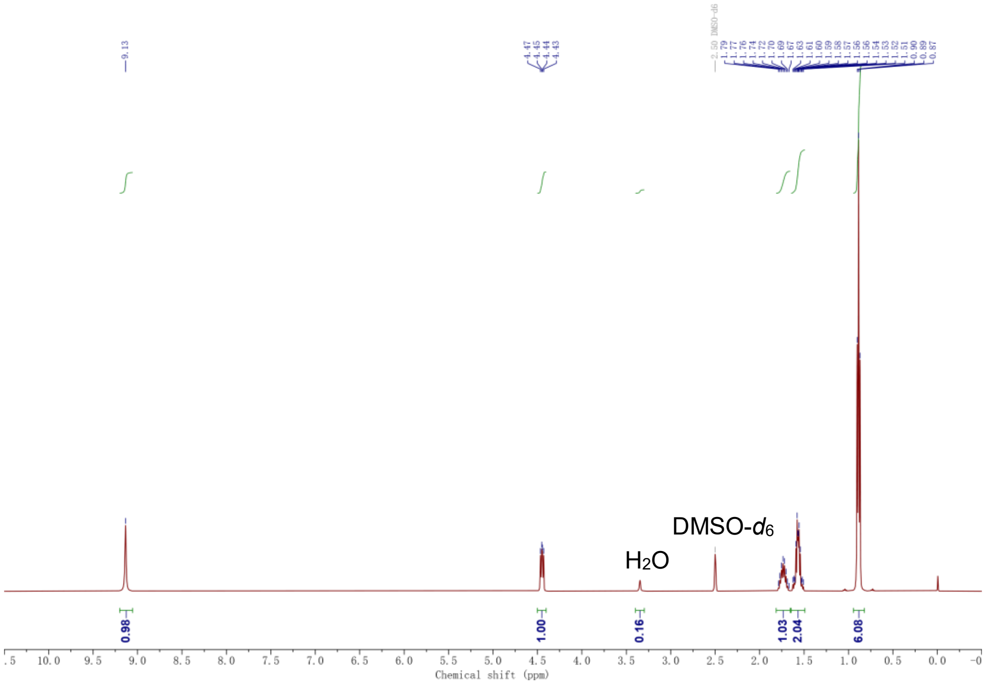

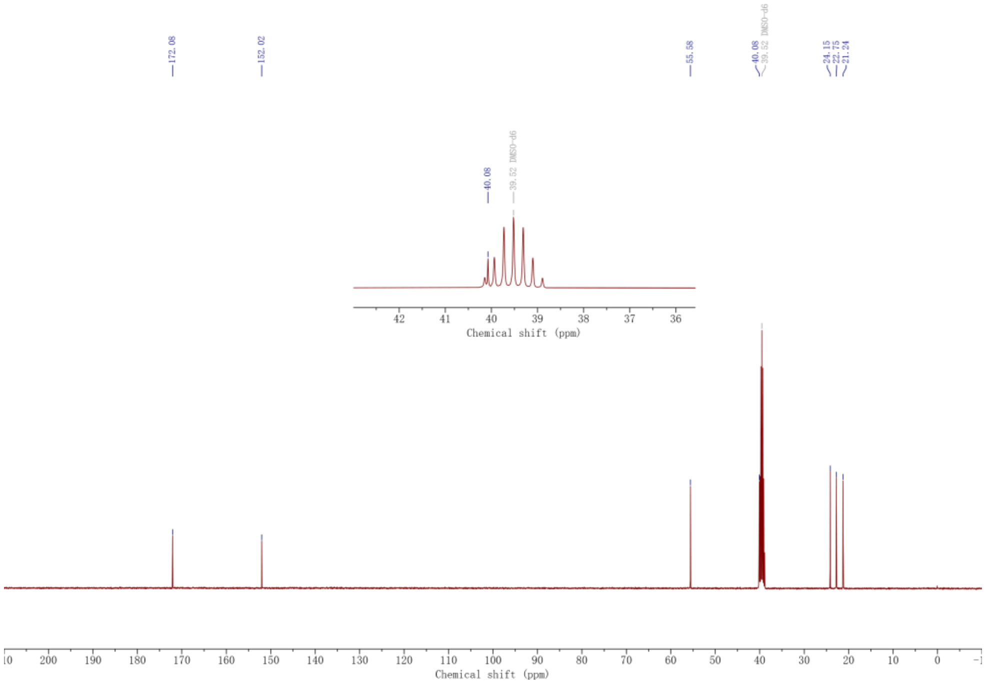

Supplementary Figure 13 ^{1}H and ^{13}C NMR spectra of 3g in DMSO-d_6.

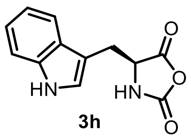

3h

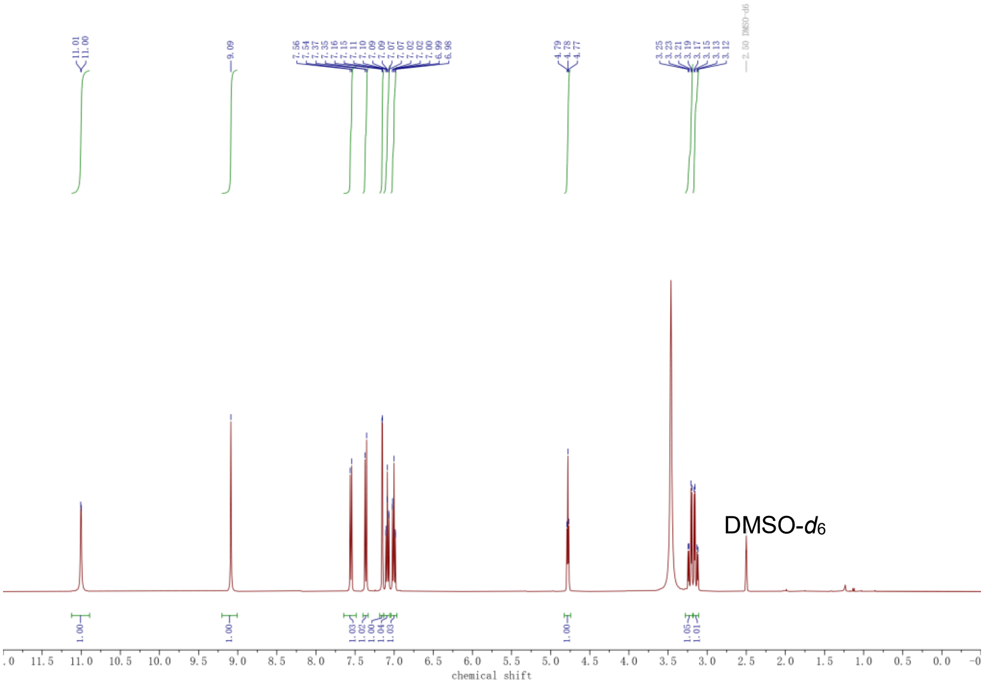

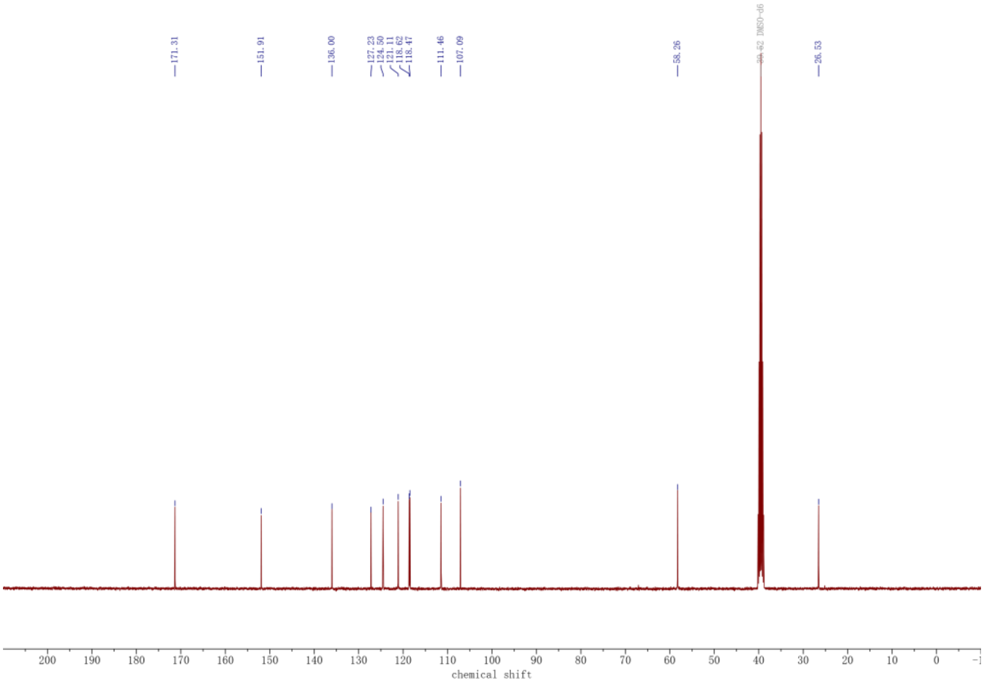

Supplementary Figure 14 ^{1}H and ^{13}C NMR spectra of 3h in DMSO-*d*$_6$.

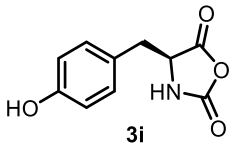

3i

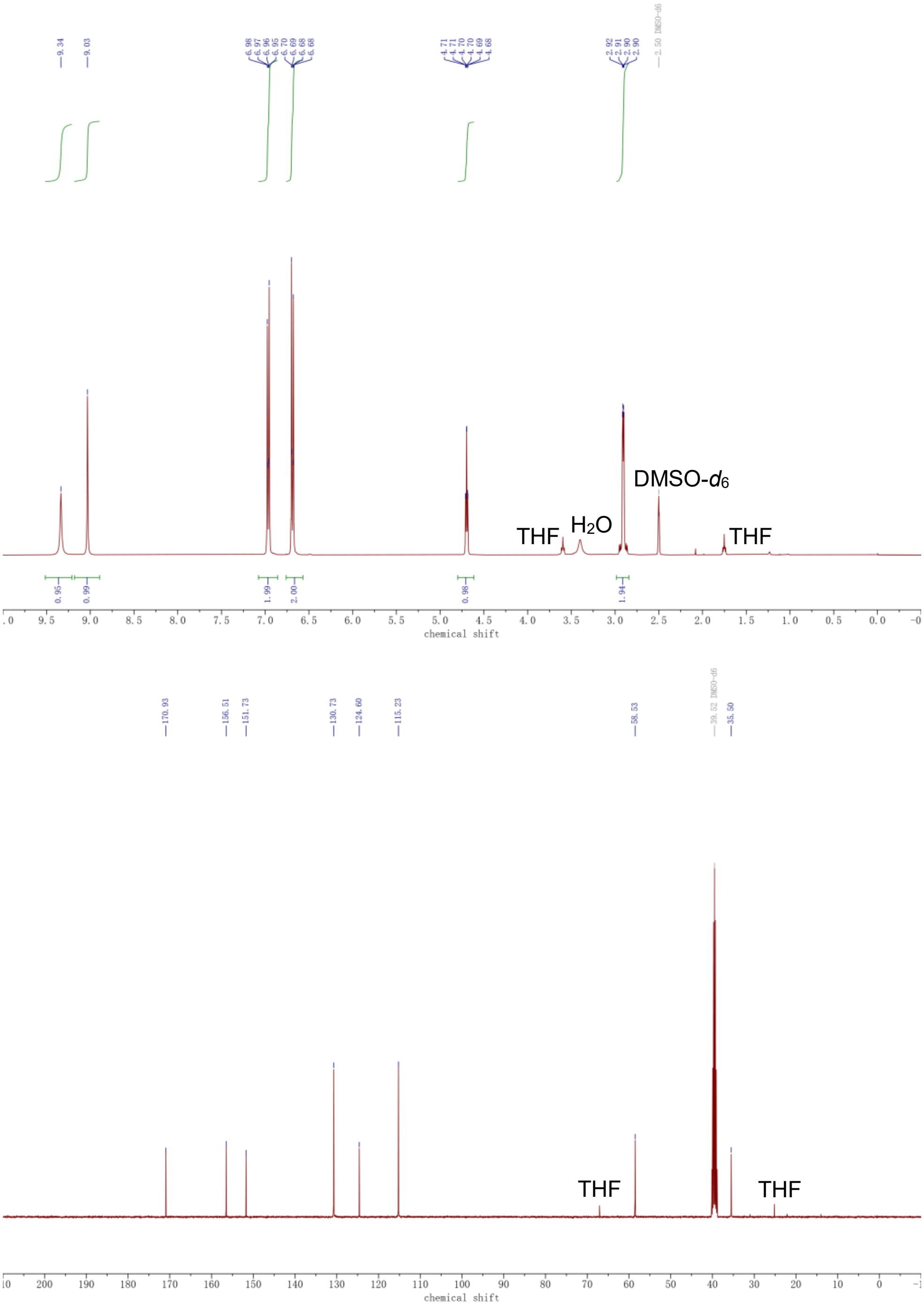

Supplementary Figure 15 ^{1}H and ^{13}C NMR spectra of 3i in DMSO-d_6.

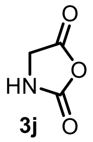

3j

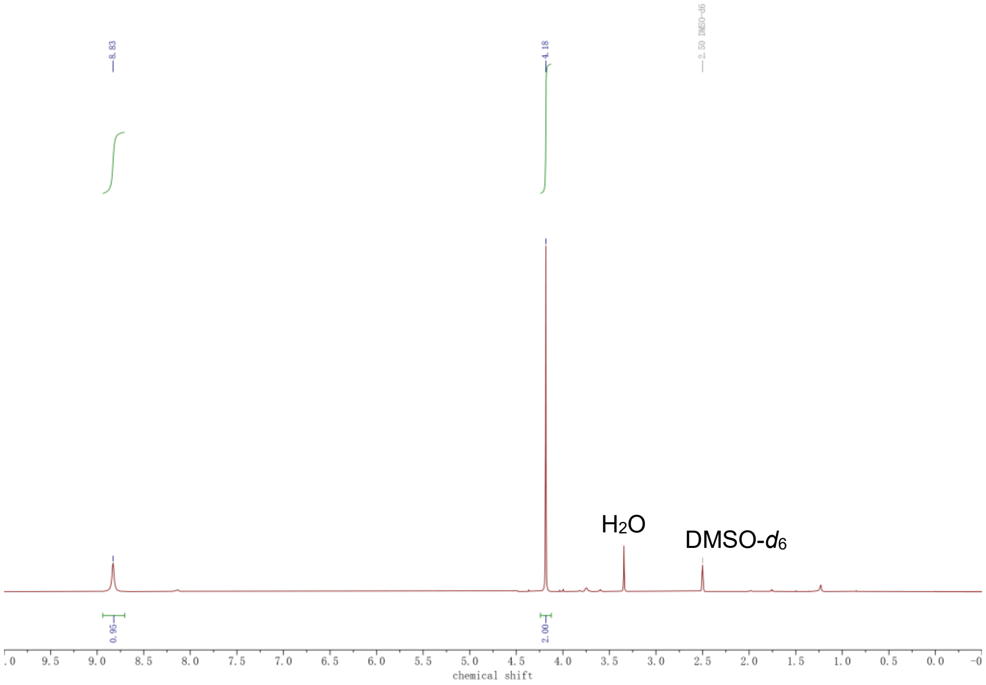

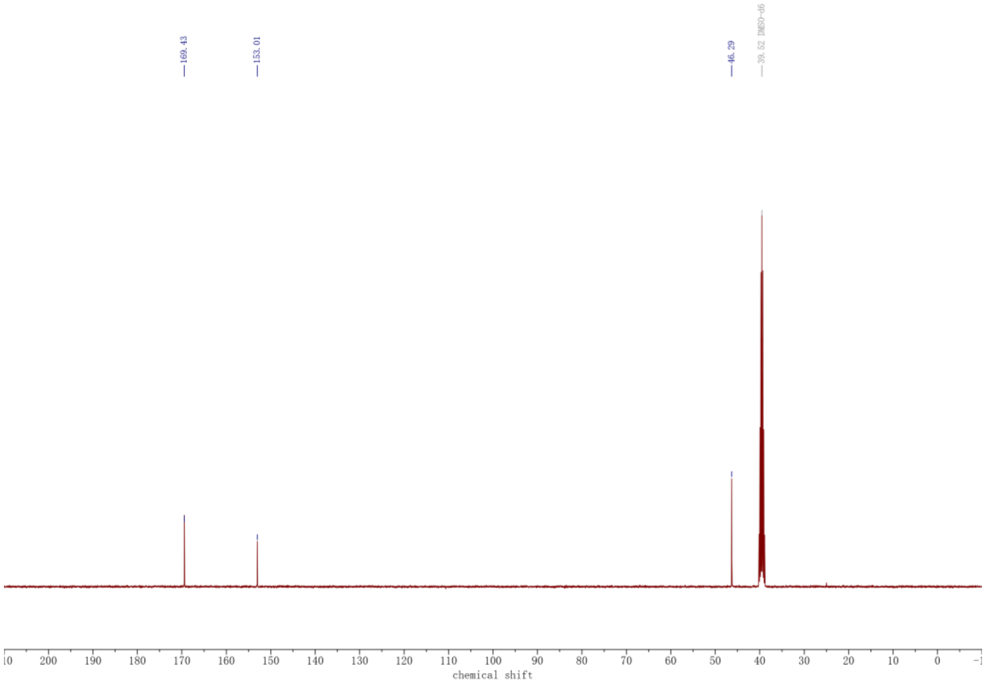

Supplementary Figure 16 ^{1}H and ^{13}C NMR spectra of 3j in DMSO-d_6.

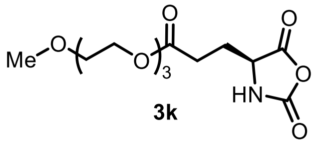

3k

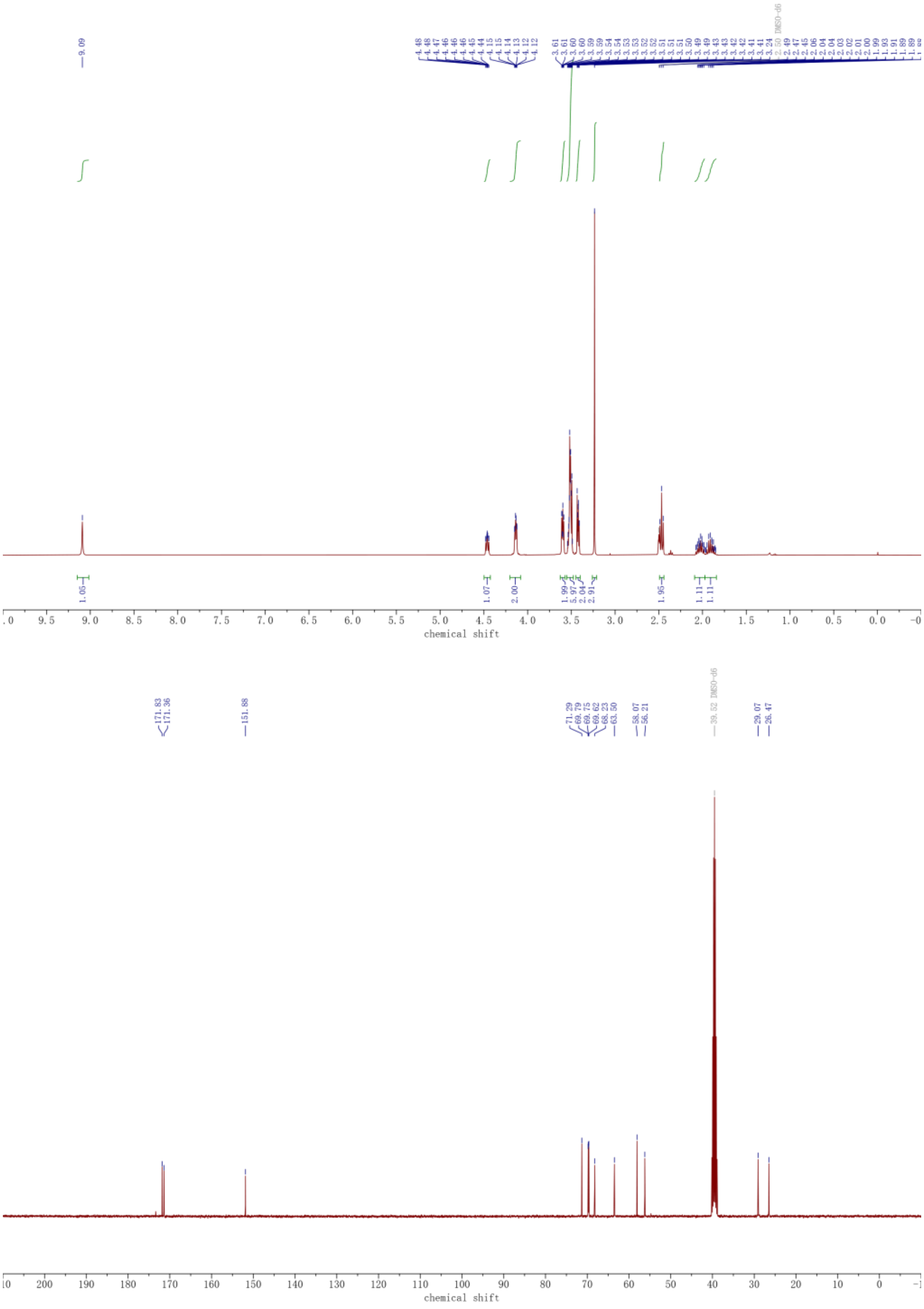

Supplementary Figure 17 ^{1}H and ^{13}C NMR spectra of 3k in DMSO-d_6.

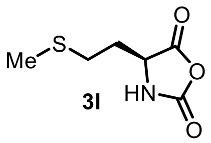

3l

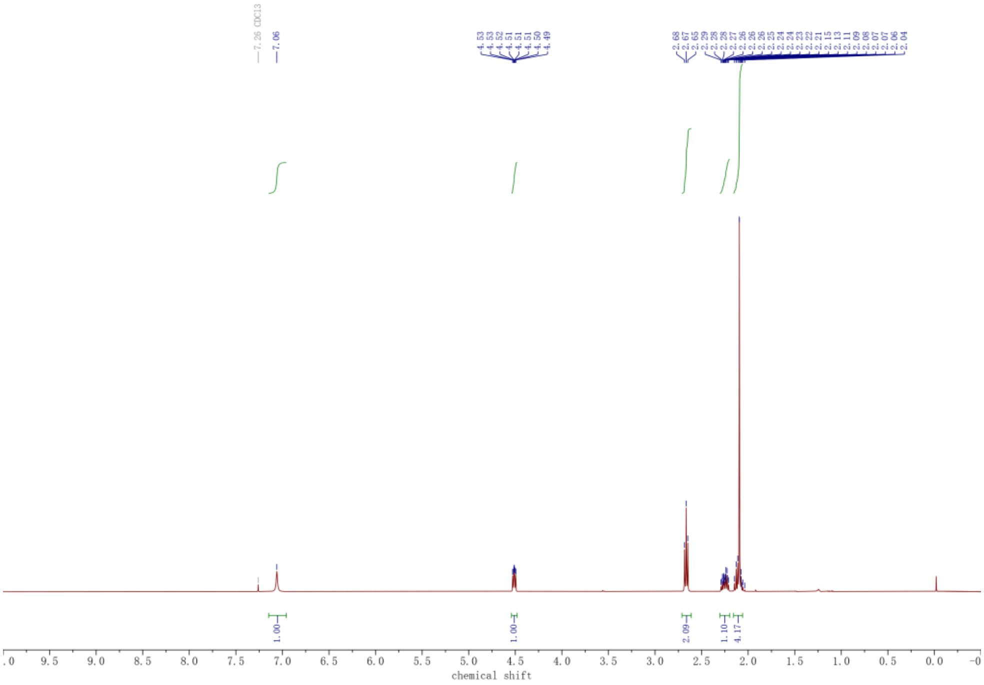

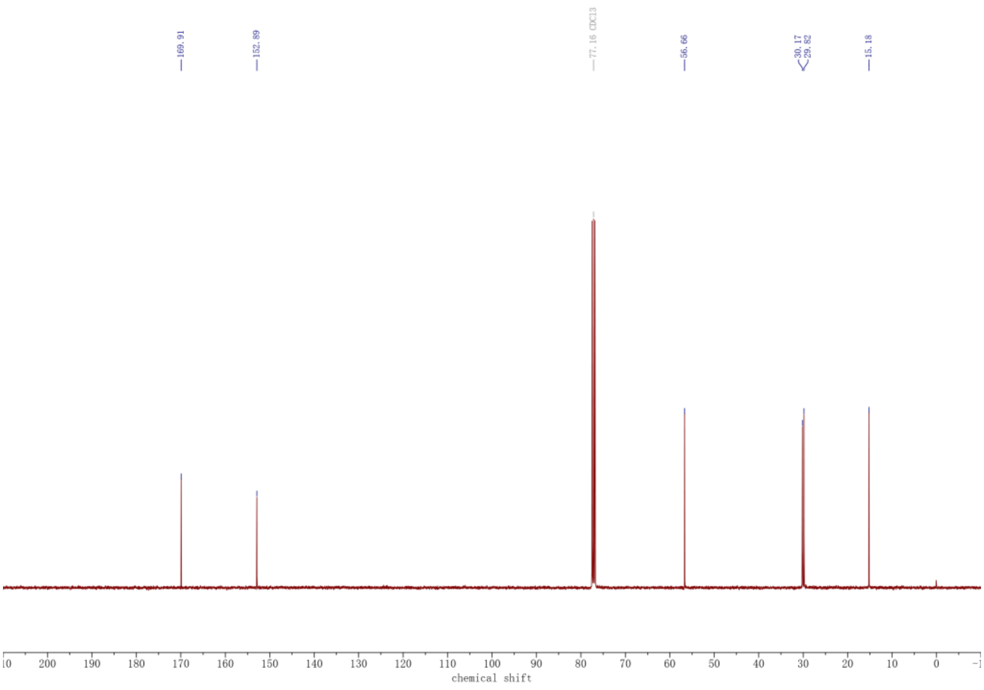

Supplementary Figure 18 ^{1}H and ^{13}C NMR spectra of 3l in $CDCl_3$.

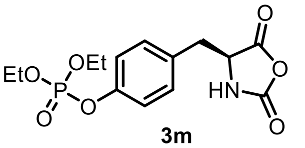

3m

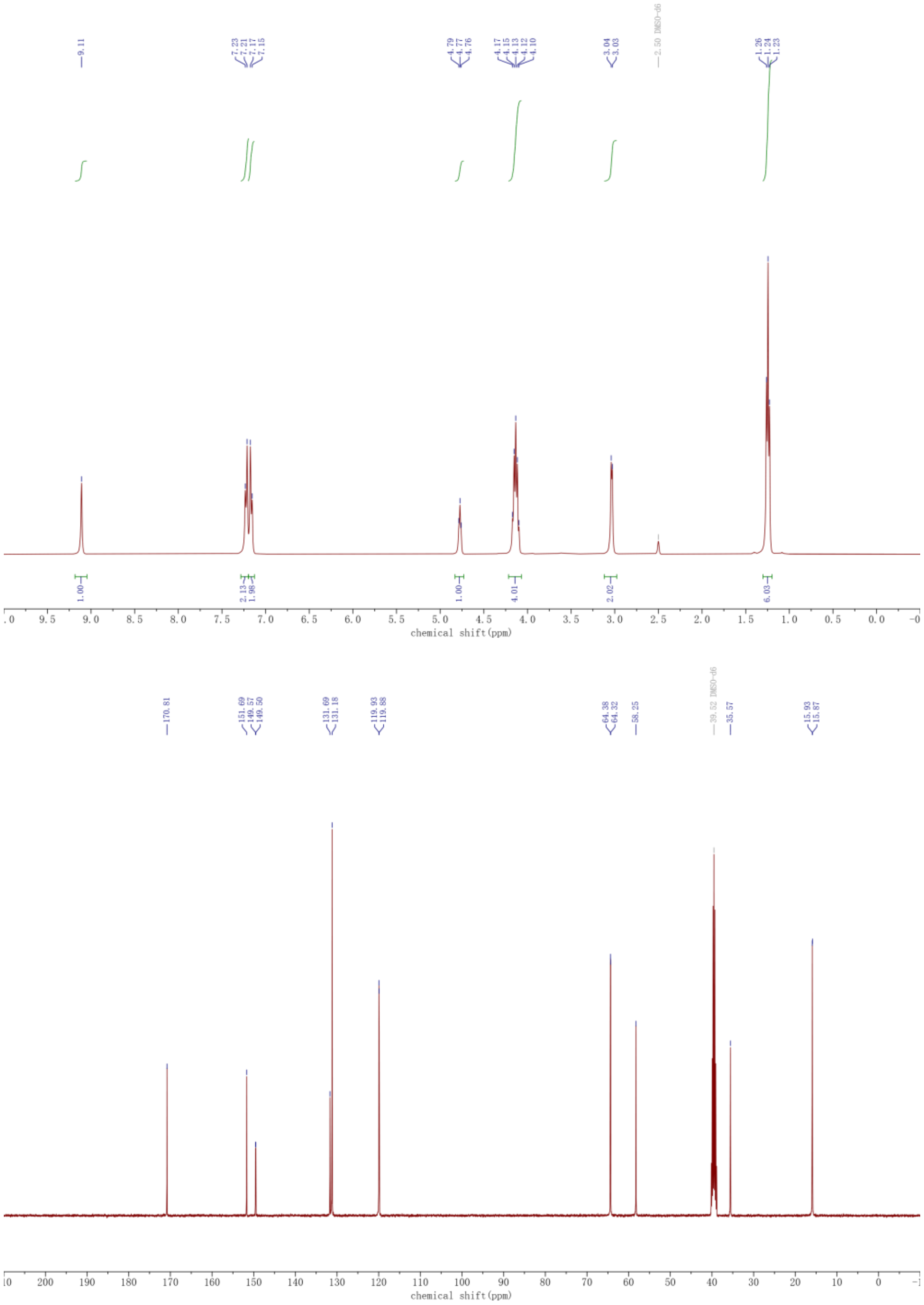

Supplementary Figure 19 ^{1}H and ^{13}C NMR spectra of 3m in DMSO-d_6.

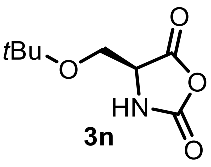

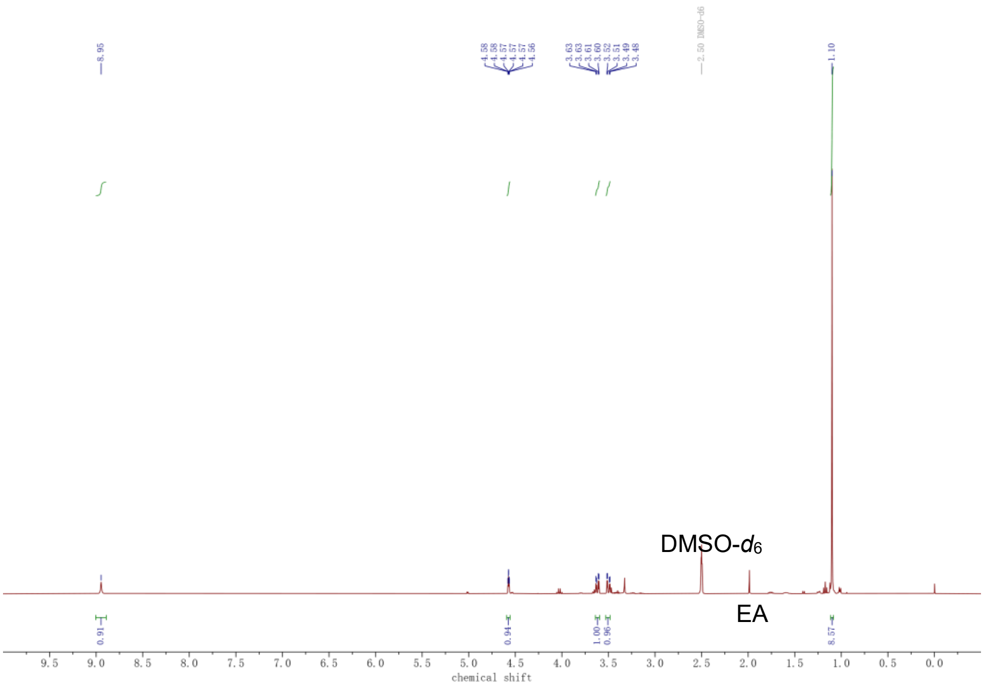

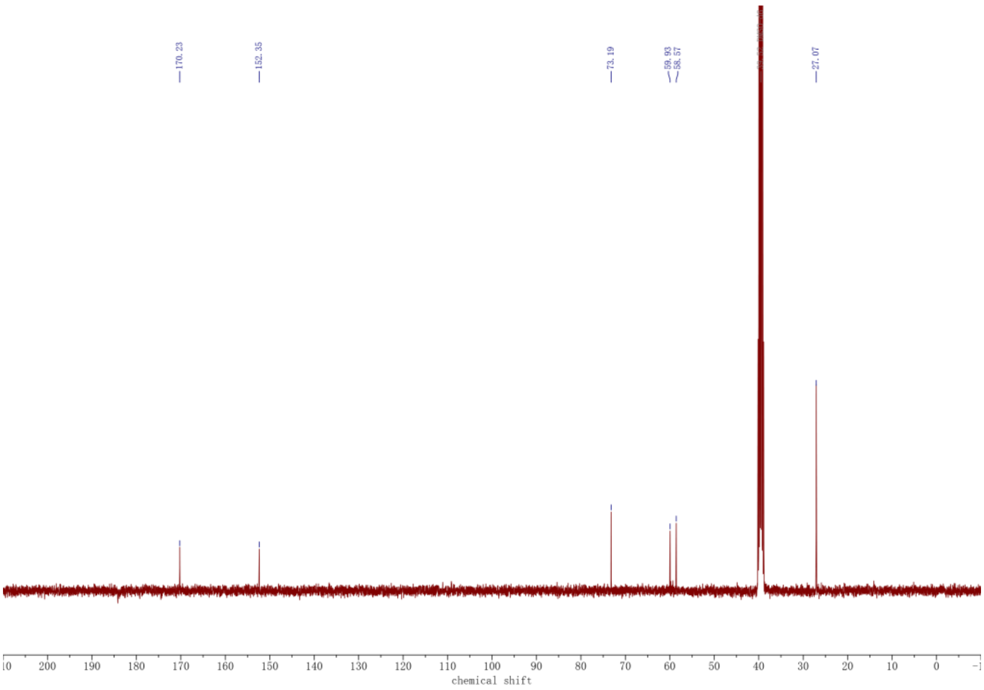

Supplementary Figure 20 ^{1}H and ^{13}C NMR spectra of 3n in DMSO-*d*$_6$.

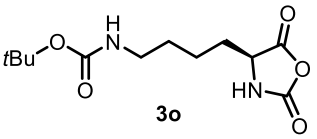

3o

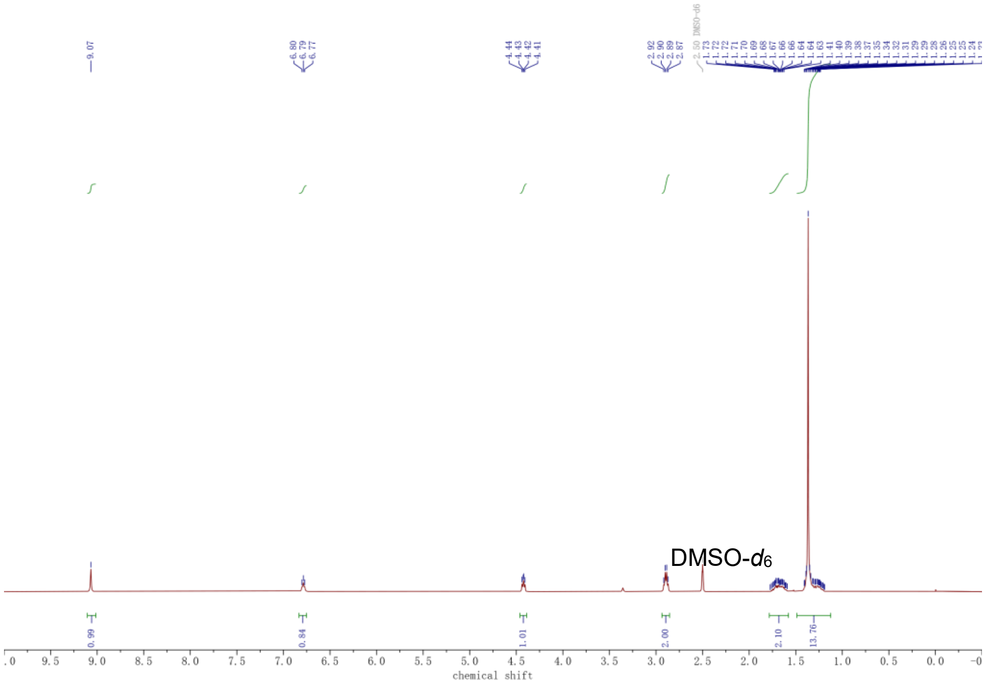

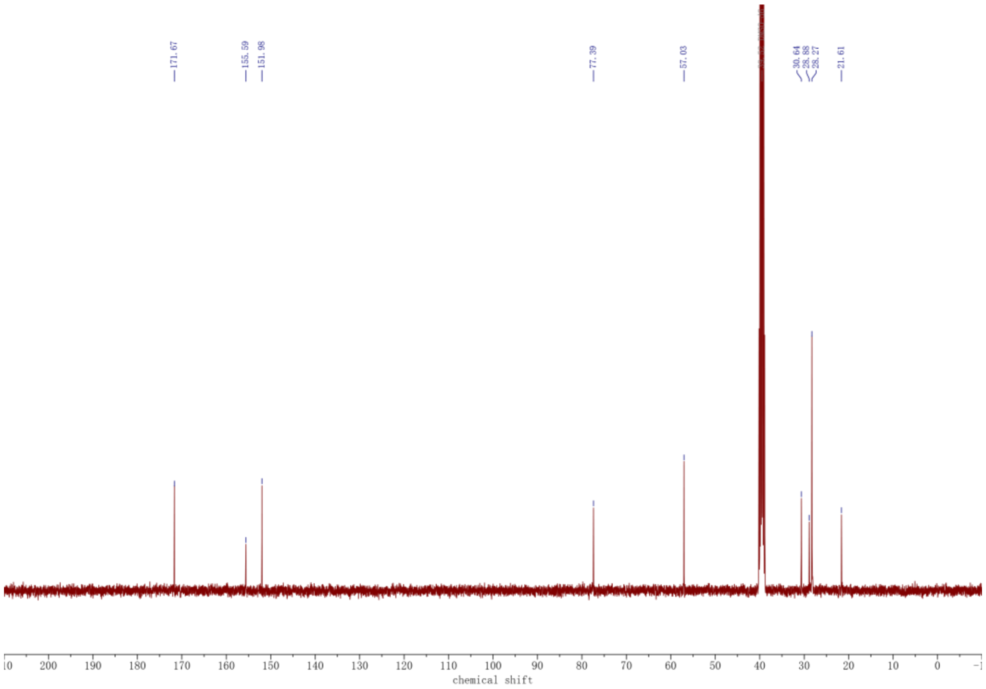

Supplementary Figure 21 ^{1}H and ^{13}C NMR spectra of 3o in DMSO-d_6.

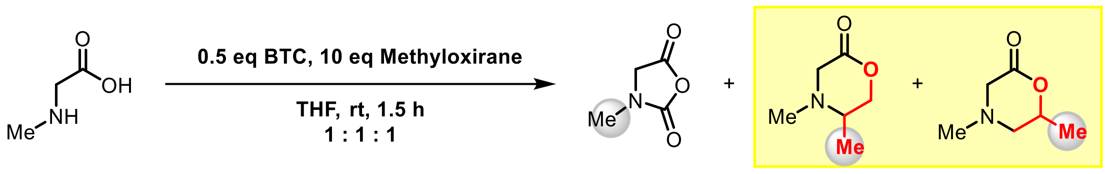

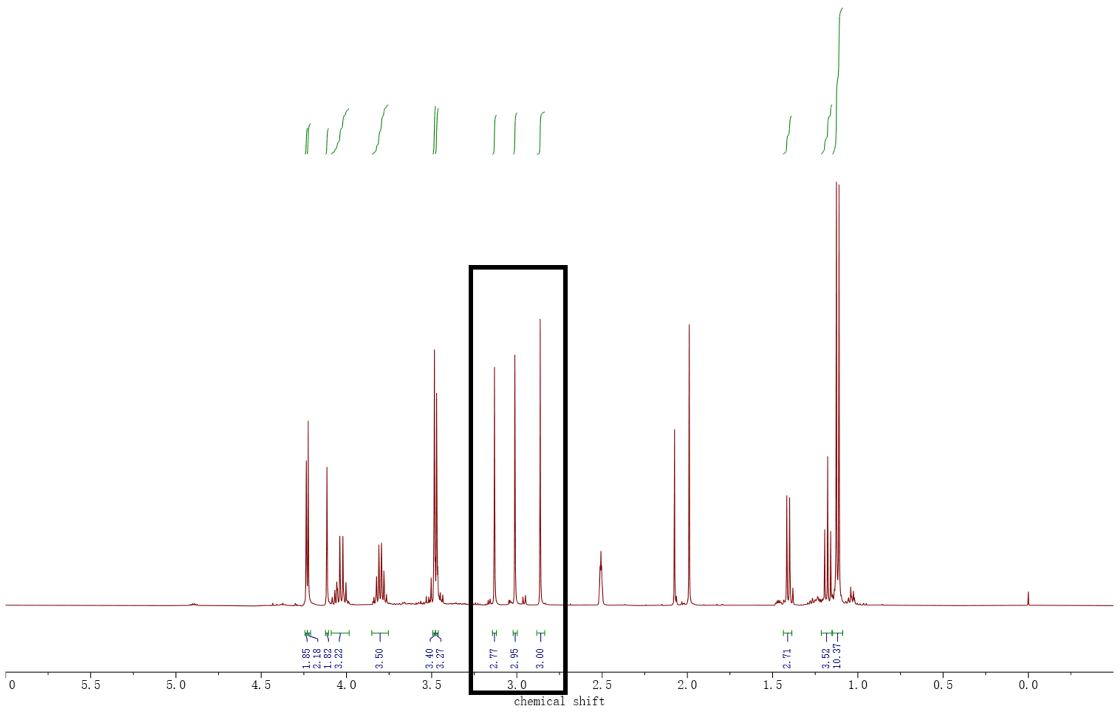

Supplementary Figure 22 Side products analyses in DMSO-d_6. The ratio of products was based on the integrate of methyl group on nitrogen atom.

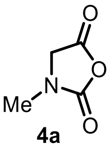

4a

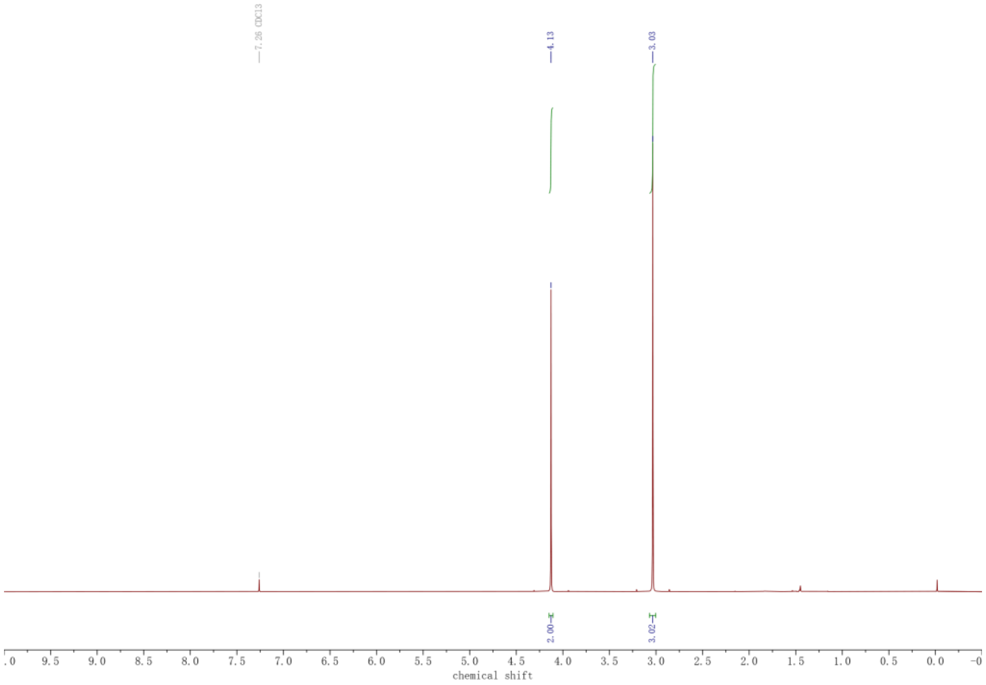

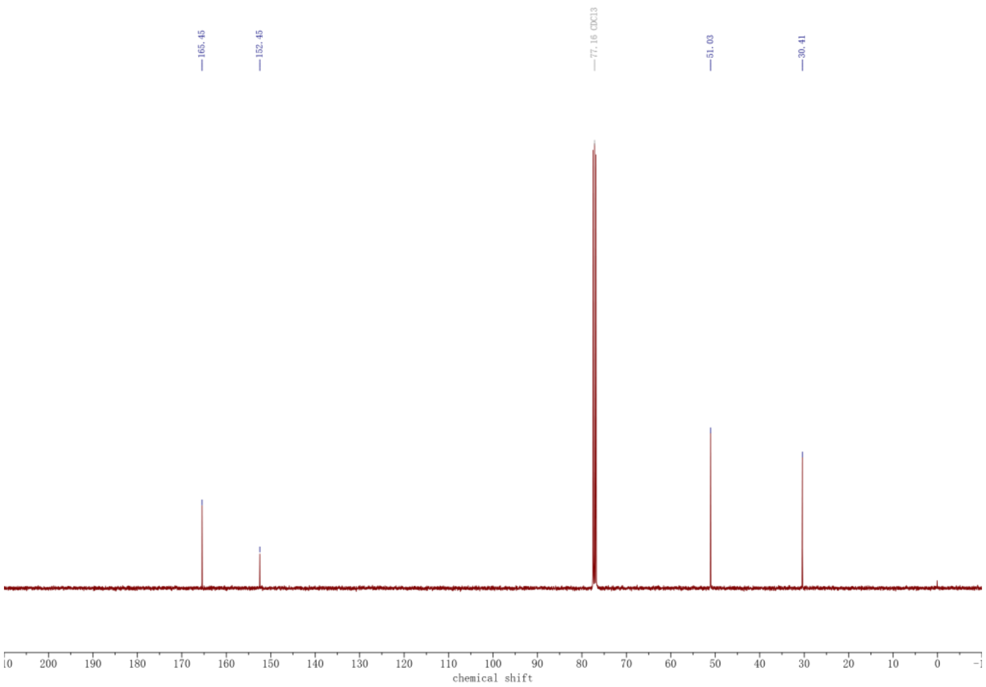

Supplementary Figure 23 ^{1}H and ^{13}C NMR spectra of 4a in CDCl$_3$.

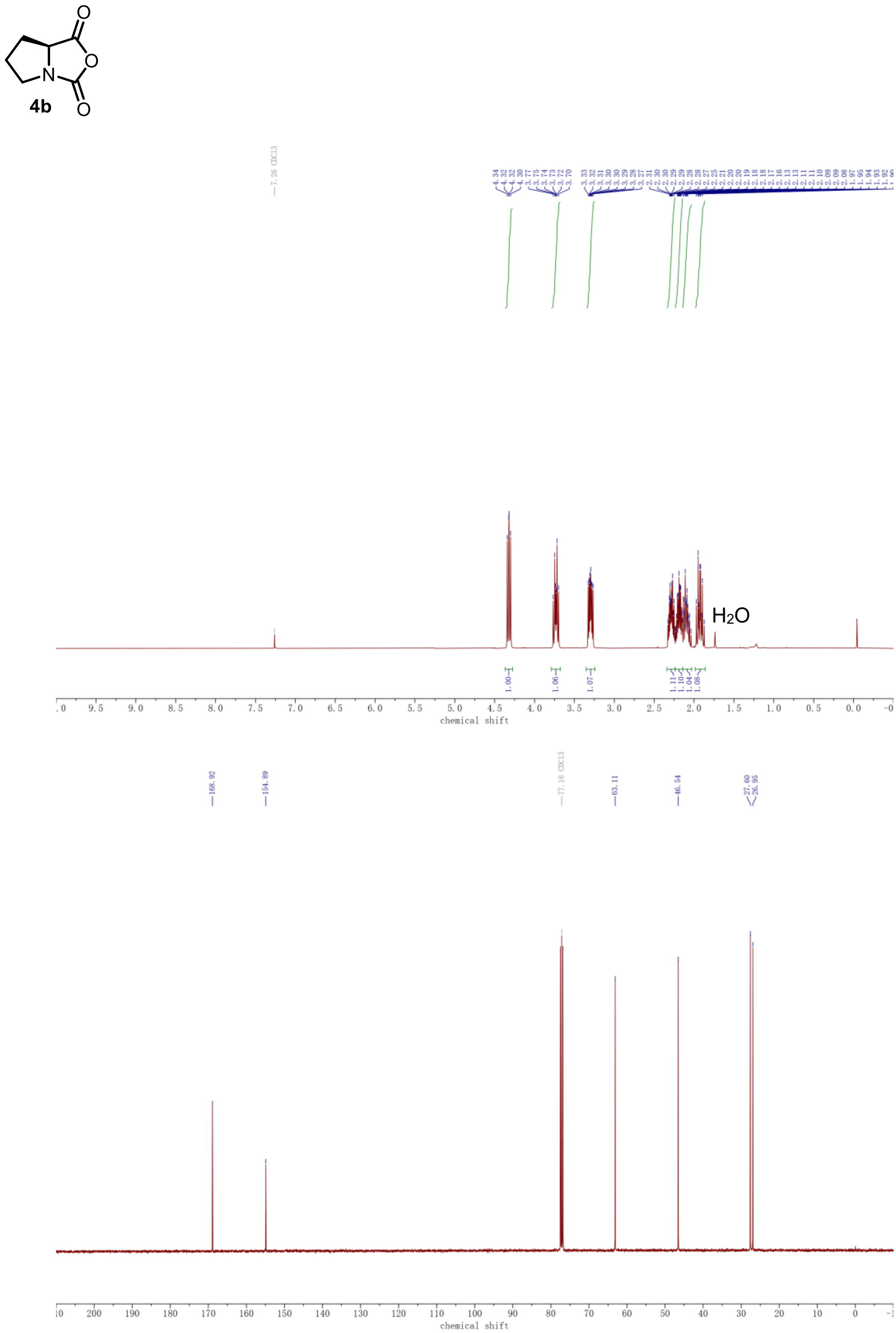

Supplementary Figure 24 ^{1}H and ^{13}C NMR spectra of 4b in $CDCl_3$.

Supplementary Table 2 ROP of Bn-Glu NCA, Sar NCA, and EG3-GluNCA by various initiators

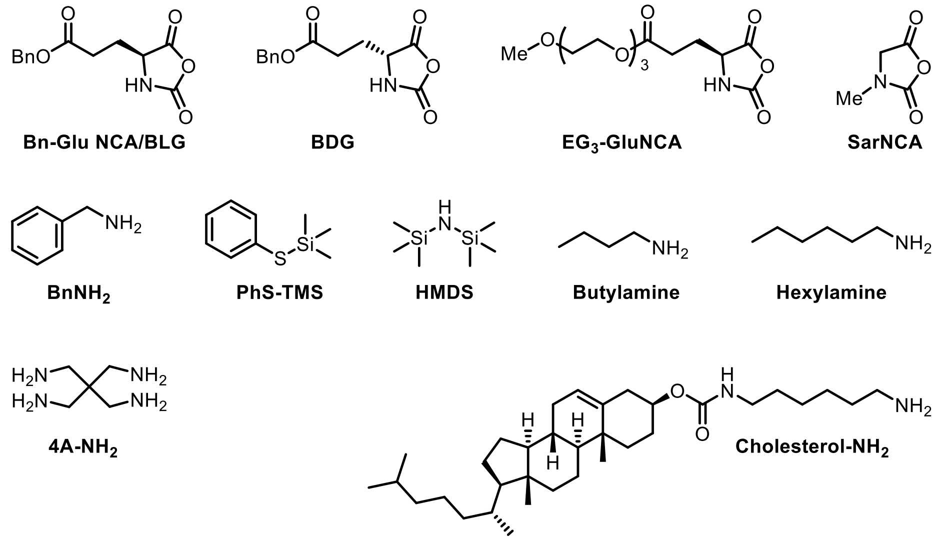

Entry	Monomer	Initiator	$[M]_0$	$[M]_0/[I]_0$	Time	Conv.	Solvent	$M_{n\ cal}$ ($\times 10^4$ g mol^{-1})	$^a M_{n\ obt}$ ($\times 10^4$ g mol^{-1})	$^a Đ$
**1**	Bn-Glu NCA (Tradition)	$BnNH_2$	0.20 M	50	12	> 99%	DMF	1.1	0.7	1.24
**2**	Bn-Glu NCA (Tradition)	$BnNH_2$	0.20 M	100	12 h	> 99%	DMF	2.2	1.5	1.05
**3**	Bn-Glu NCA	$BnNH_2$	0.20 M	50	12 h	> 99%	DMF	1.1	1.5	1.05
**4**	Bn-Glu NCA	$BnNH_2$	0.20 M	100	12 h	> 99%	DMF	2.2	2.2	1.05
**5**	BLG/BDG	$BnNH_2$	0.20 M	50	22 h	> 99%	DMF	1.0	1.0	1.06
**6**	EG_3-GluNCA (Batch 1)	PhS-TMS	0.15 M	75	24 h	> 99%	DMF	2.1	2.0	1.07
**7**	EG_3-GluNCA (Batch 2)	PhS-TMS	0.15 M	75	20 h	> 99%	DMF	2.1	2.0	1.06
**8**	EG_3-GluNCA (Batch 2)	HMDS	0.15 M	73	24 h	> 99%	DMF	2.0	2.0	1.13
**9**	EG_3-GluNCA (Batch 3)	HMDS	0.31 M	73	12 h	> 99%	DMF	2.0	1.9	1.05
**10**	EG_3-GluNCA (Batch 3)	HMDS	0.31 M	73	18 h	> 99%	DMF	2.0	1.9	1.07
**11**	EG_3-GluNCA (Batch 3)	HMDS	0.31 M	73	12 h	> 99%	DMF	2.0	1.9	1.07
**12**	EG_3-GluNCA (Batch 4)	HMDS	0.31 M	77	22 h	> 99%	DMF	2.1	1.9	1.08
**13**	EG_3-GluNCA (Batch 2)	Butylamine	0.31 M	75	12 h	> 99%	DMF	2.1	1.9	1.13

**14**	EG_3-GluNCA (Batch 5)	Hexylamine	0.31 M	50	12 h	> 99%	DMF	1.4	1.2	1.16
**15**	EG_3-GluNCA	Cholesterol-NH_2	0.2 M	40	22 h	> 99%	DMF	1.1	1.1	1.06
**16**	SarNCA	Hexylamine	1.1 M	150	12 h	> 99%	DMF	1.1	1.0	1.07
**17**	SarNCA Batch 1	4A-NH_2	1.0 M	250	16 h	> 99%	DMF	1.8	1.8	1.01
**18**	SarNCA (Batch 2)	4A-NH_2	1.0 M	250	16 h	> 99%	DMF	1.8	1.7	1.04
**19**	SarNCA (Batch 3)	4A-NH_2	1.0 M	250	16 h	> 99%	DMF	1.8	1.7	1.01
**20**	SarNCA (Batch 4)	4A-NH_2	1.0 M	250	16 h	> 99%	DMF	1.8	1.8	1.01

[a]absolute M_n obtained from SEC equipped with a multi-angle light scattering detector using 0.1 M LiBr in DMF as the mobile phase. PBLG dn/dc = 0.093 mL/g polysarcosine dn/dc = 0.096 mL/g, P(EG_3-Glu) dn/dc = 0.069 mL/g.

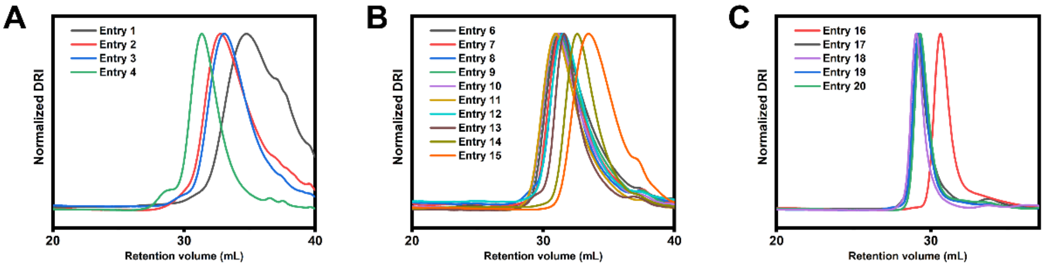

Supplementary Figure 25 (A) GPC of entry 1 to 5. (B) GPC of entry 6 to 15. (C) GPC of entry 16 to 20.

Discussion: we tested the ROP of Bn-Glu NCA, Sar NCA, and EG_3-GluNCA (Supplementary Table 2 and Supplementary Figure 25), three most frequently used monomers in our lab. Our results indicated that each of these monomers produced the corresponding polypeptide with a well-defined structure, a predictable molar mass (M_n), and narrow dispersity ($Đ$). For example, when conducted in parallel, benzyl amine ($BnNH_2$)-mediated ROP of BLG-NCA prepared from the traditional (entry 1-2) method showed poorer M_n control compared to the same reaction using monomers that were synthesized by our new method (entry 3-5). Moreover, the good reproducibility was demonstrated by our observation that the M_n and $Đ$ of the polypeptide products remained largely unchanged regardless of the monomer batch or who performed the synthesis (entry 6&7, entry 8-11, and entry 17-20). We also tested a variety of initiators, including $BnNH_2$ (entry 3-5), hexyl amine (entry 14 and 16), butyl amine (entry 13), cholesterol amine (entry 15), a four-armed amine initiator (4A-NH_2, entry 17-20), Hexamethyldisilazane (HMDS, entry 8-12), and trimethylsilyl phenylsulfide (PhSTMS, entry 6-7). The results revealed that our

new method could confer satisfactory and consistent control of both M_n and $Đ$. The reproducibility of entry 6&7 is remarkable because PhSTMS-mediated ROP is very sensitive to the purity of monomer based on own experience. All together, these experimental data provided sufficient evidence that the NCAs prepared by the method detailed in this study (with PO or ECH) have the same, if not higher, quality and purity as those afforded by the traditional approach.

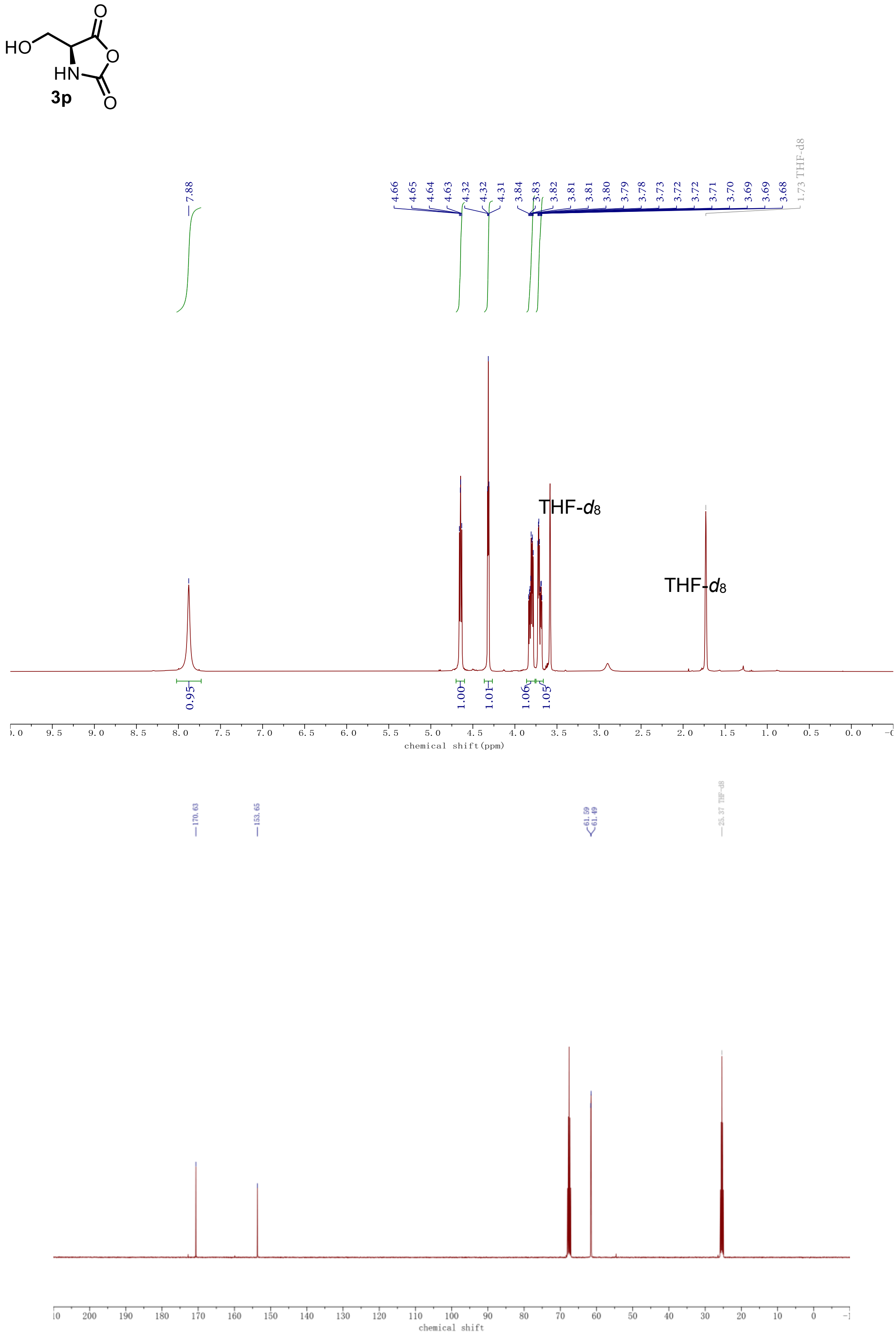

Supplementary Figure 26 ^{1}H and ^{13}C NMR spectra of 3p in THF-d_8.

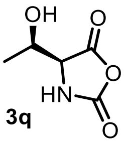

3q

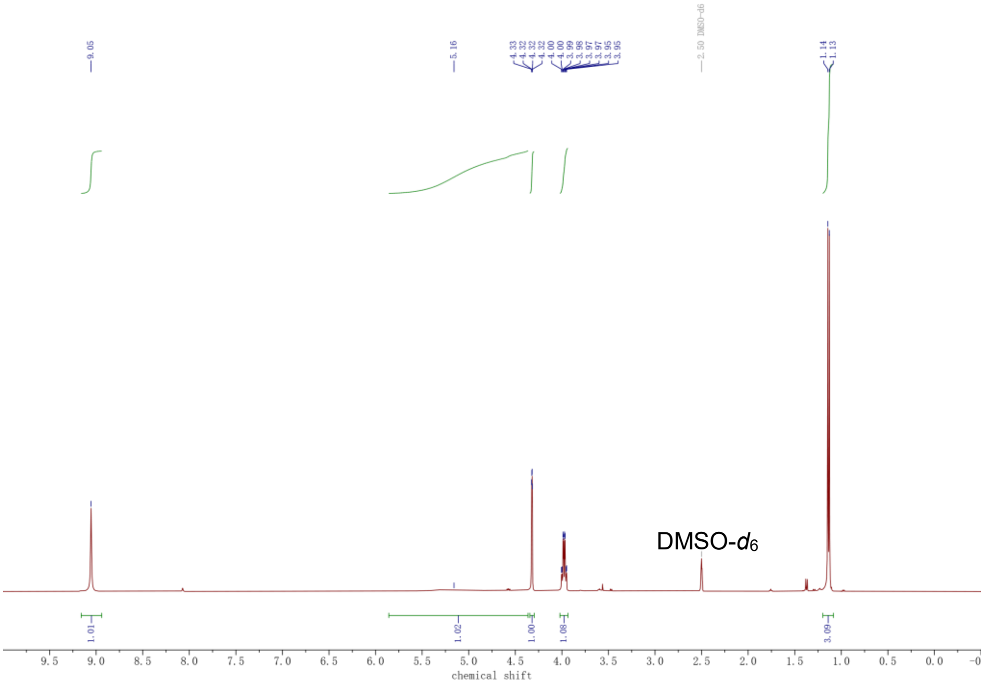

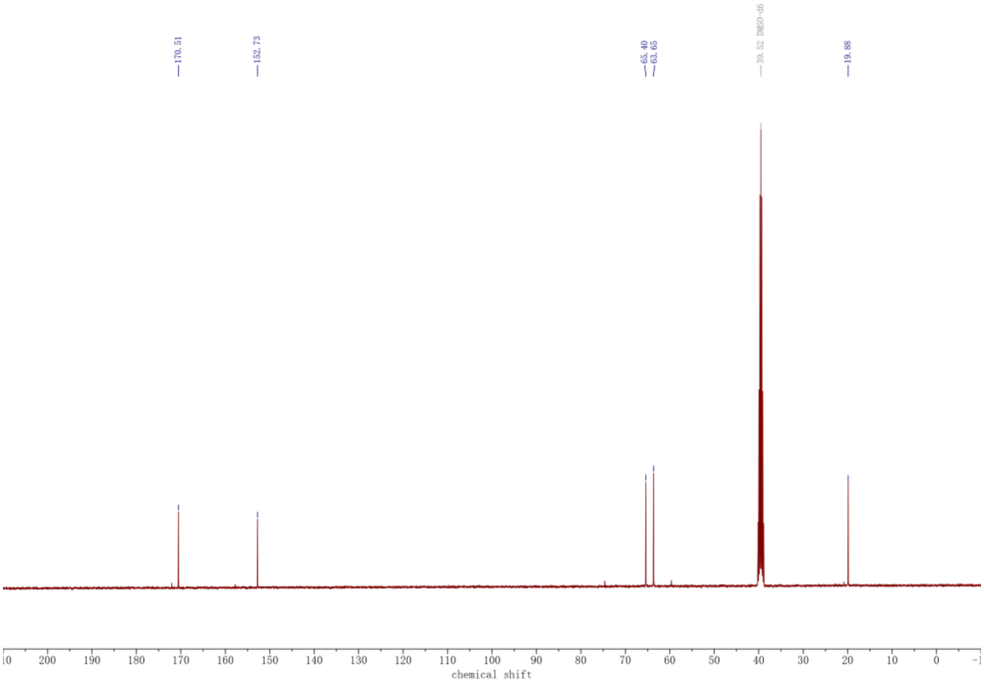

Supplementary Figure 27 ^{1}H and ^{13}C NMR spectra of 3q in DMSO-*d*$_6$.

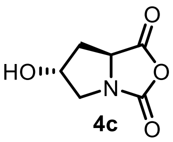

4c

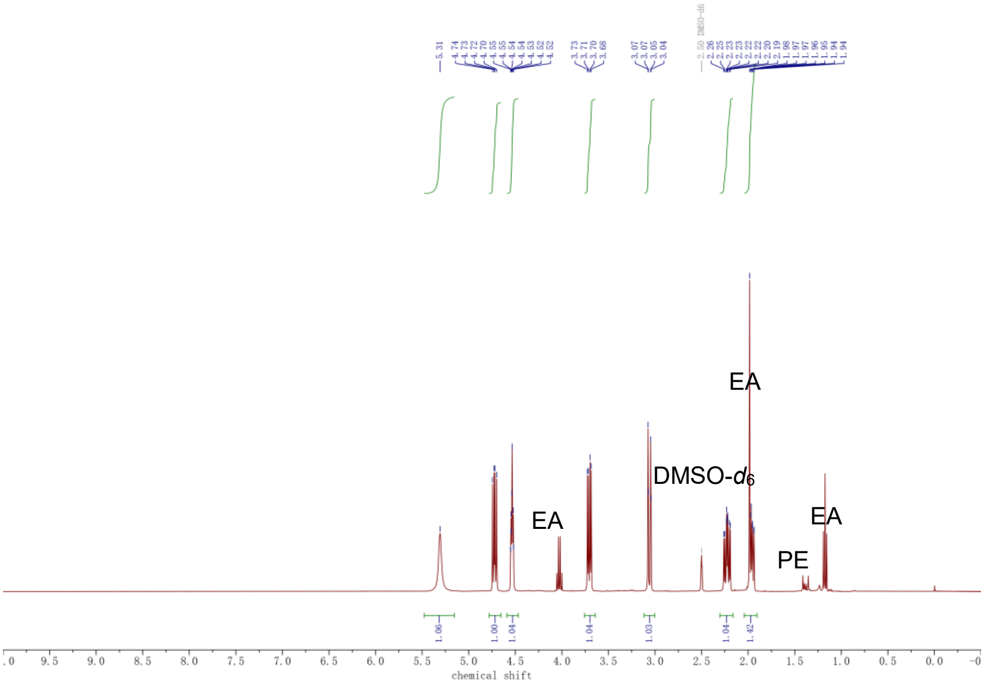

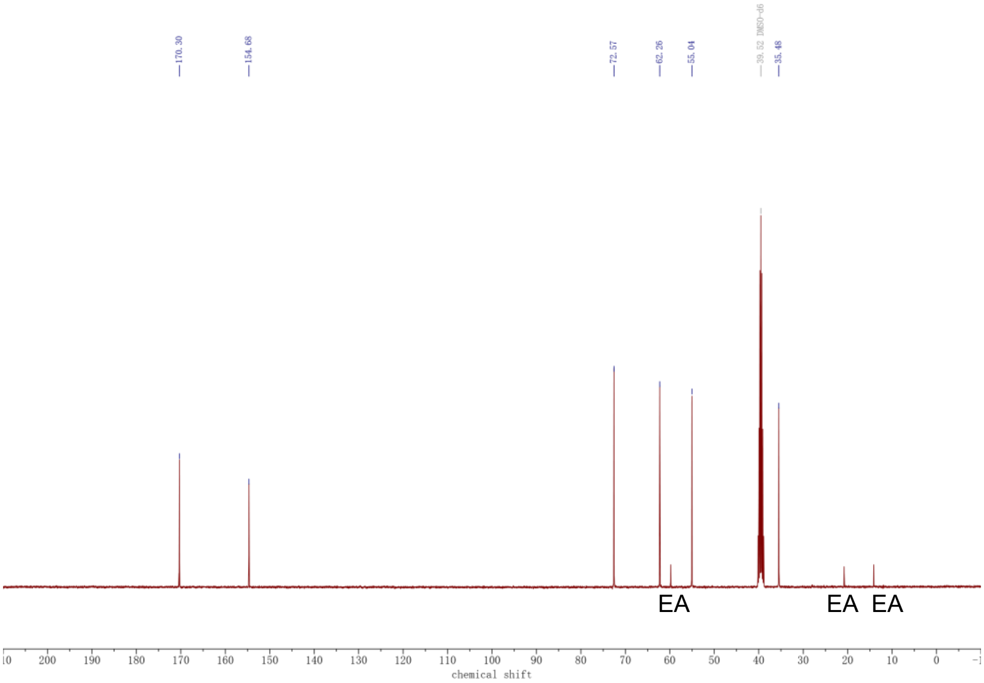

Supplementary Figure 28 ^{1}H and ^{13}C NMR spectra of 4c in DMSO-d_6.

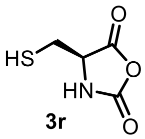

3r

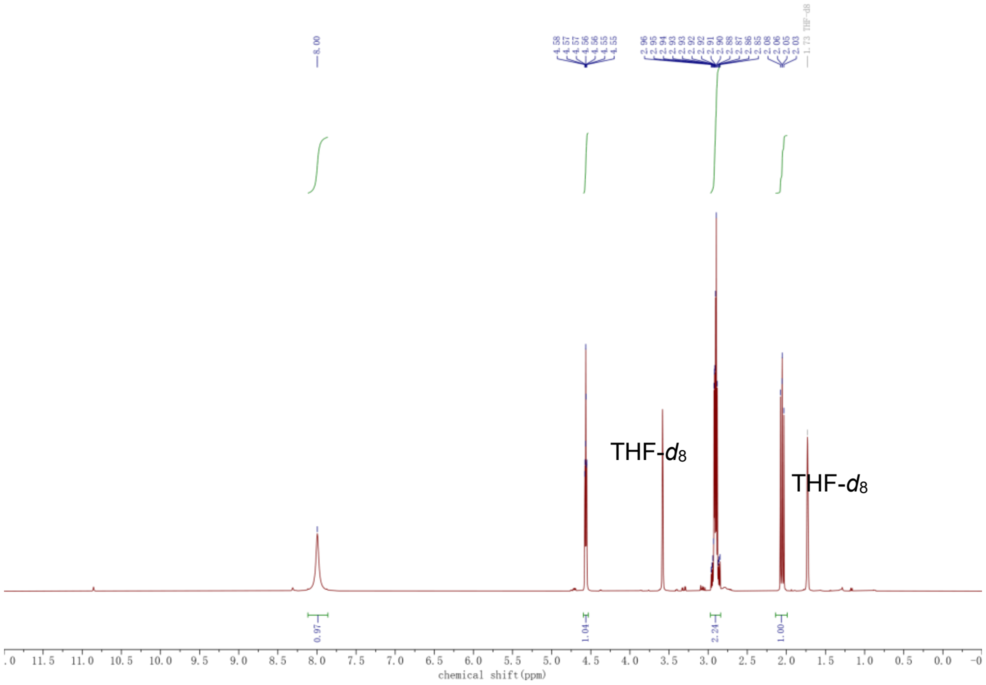

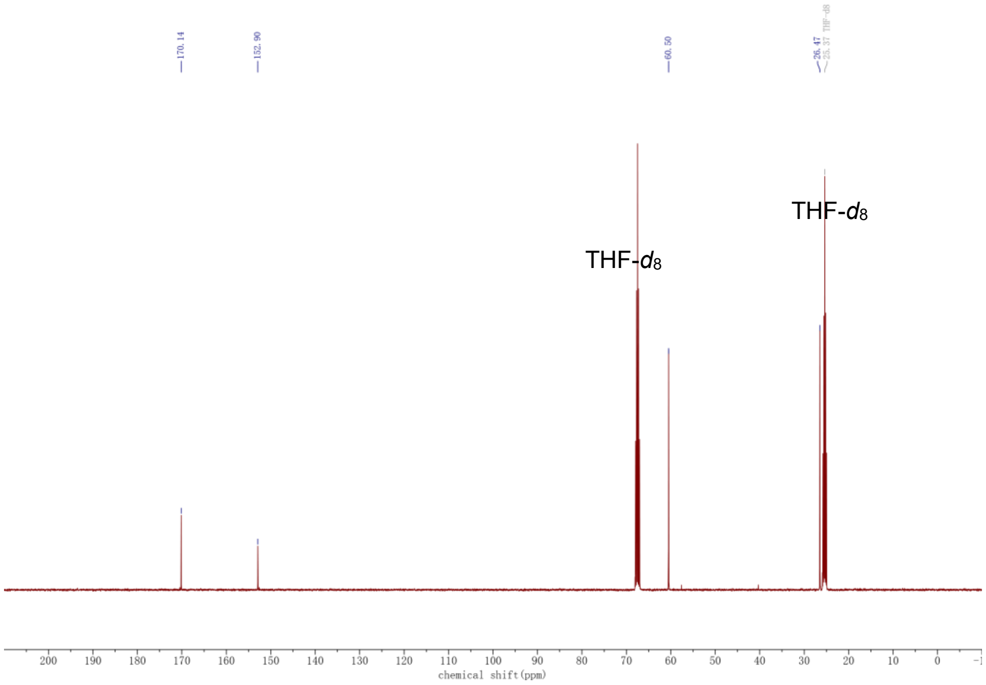

Supplementary Figure 29 ^{1}H and ^{13}C NMR spectra of 3r in THF-d_8.

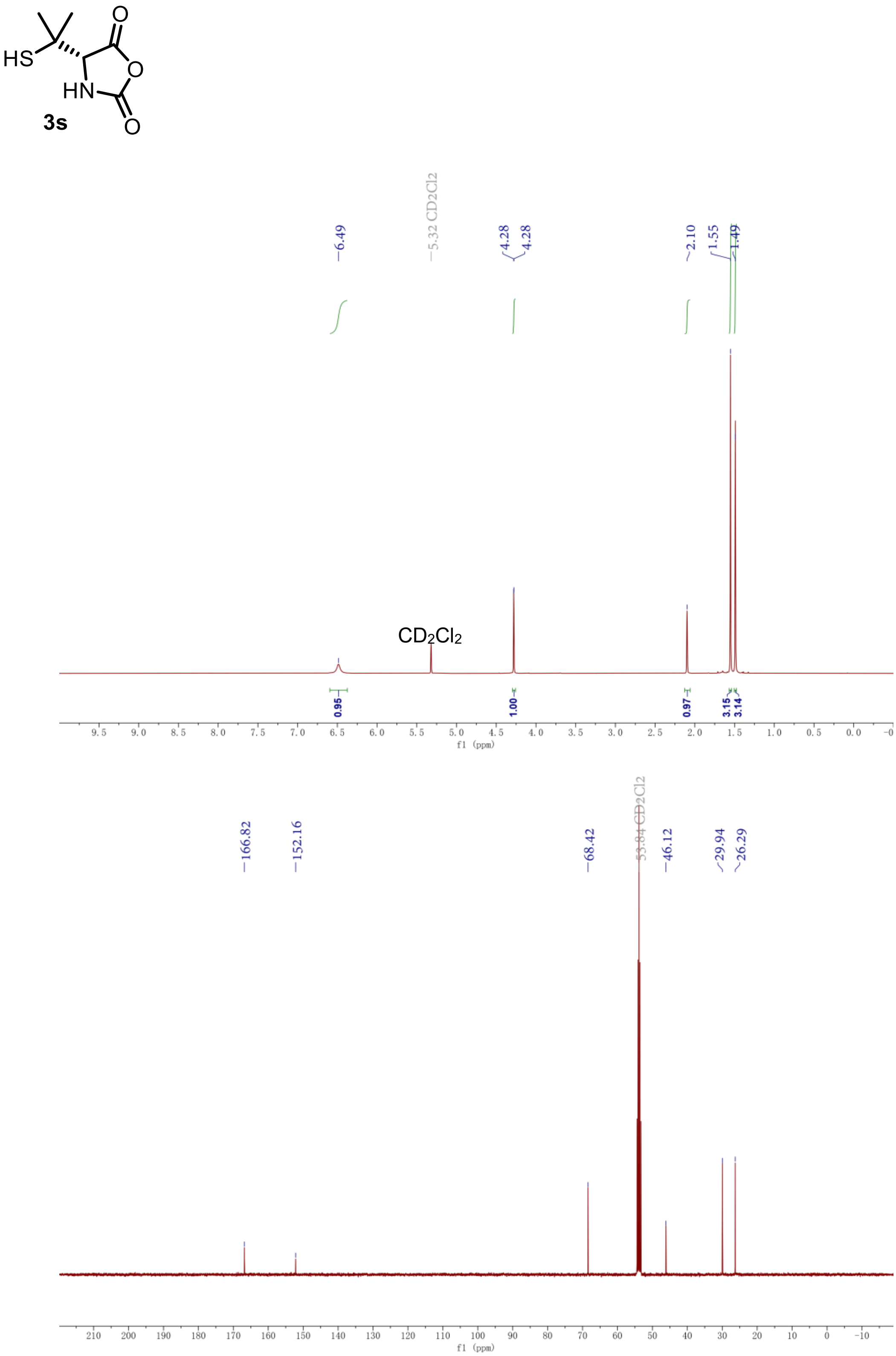

Supplementary Figure 30 ^{1}H and ^{13}C NMR spectra of 3s in CD_2Cl_2.

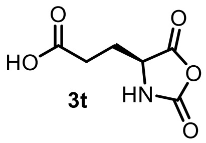

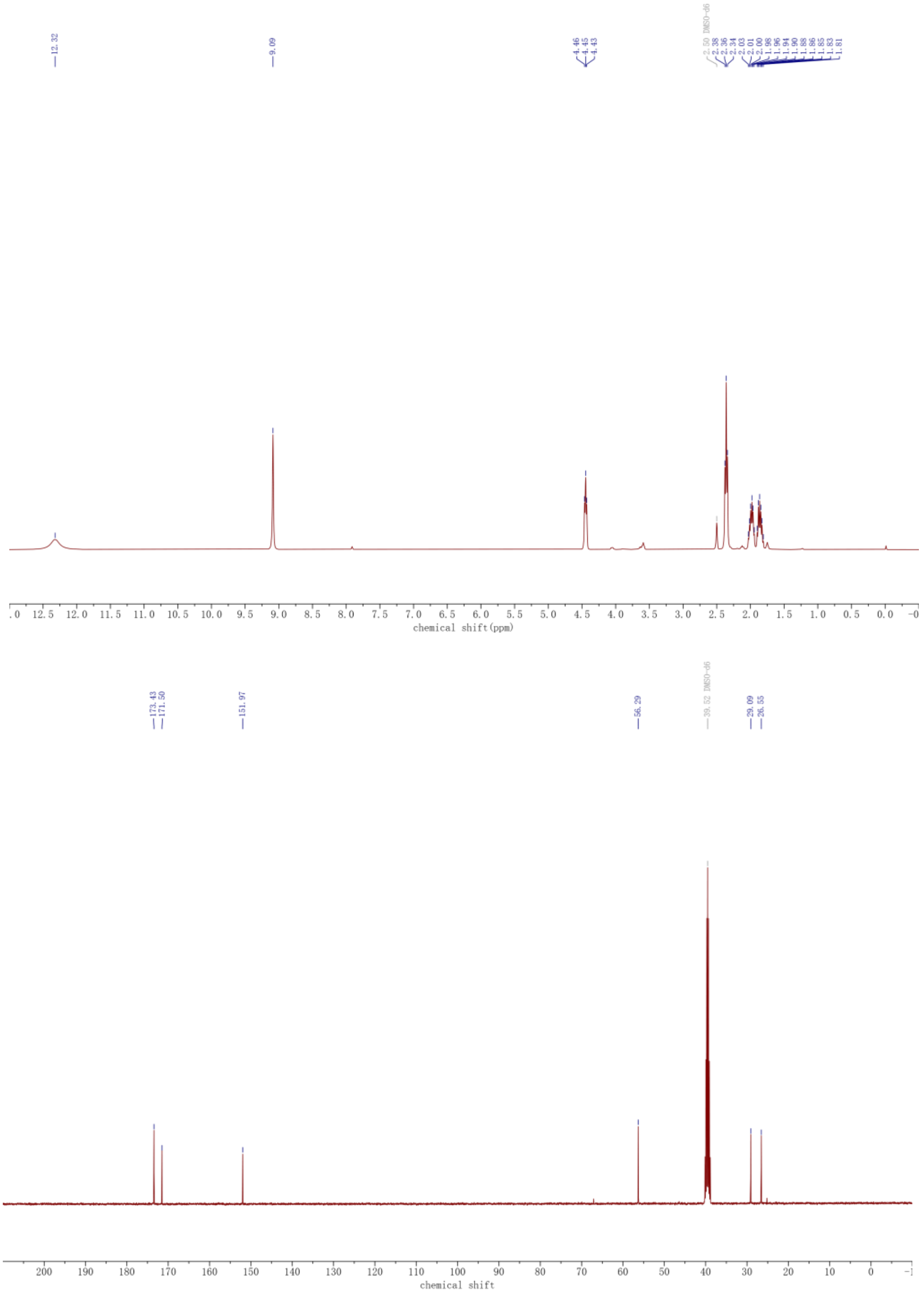

Supplementary Figure 31 ^{1}H and ^{13}C NMR spectra of 3t in DMSO-d_6.

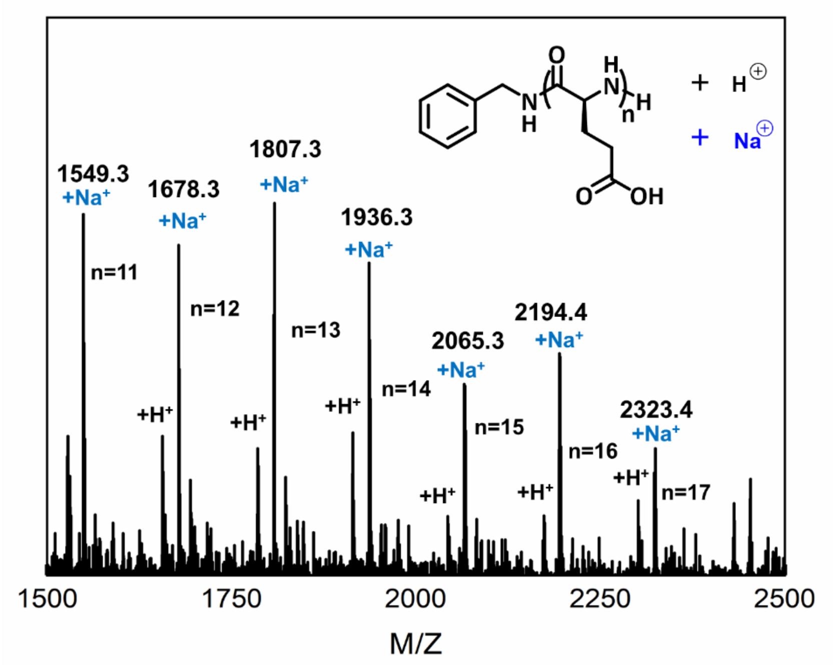

Supplementary Figure 32 MALDI-TOF mass spectrometry of poly(L-glutamic acid).

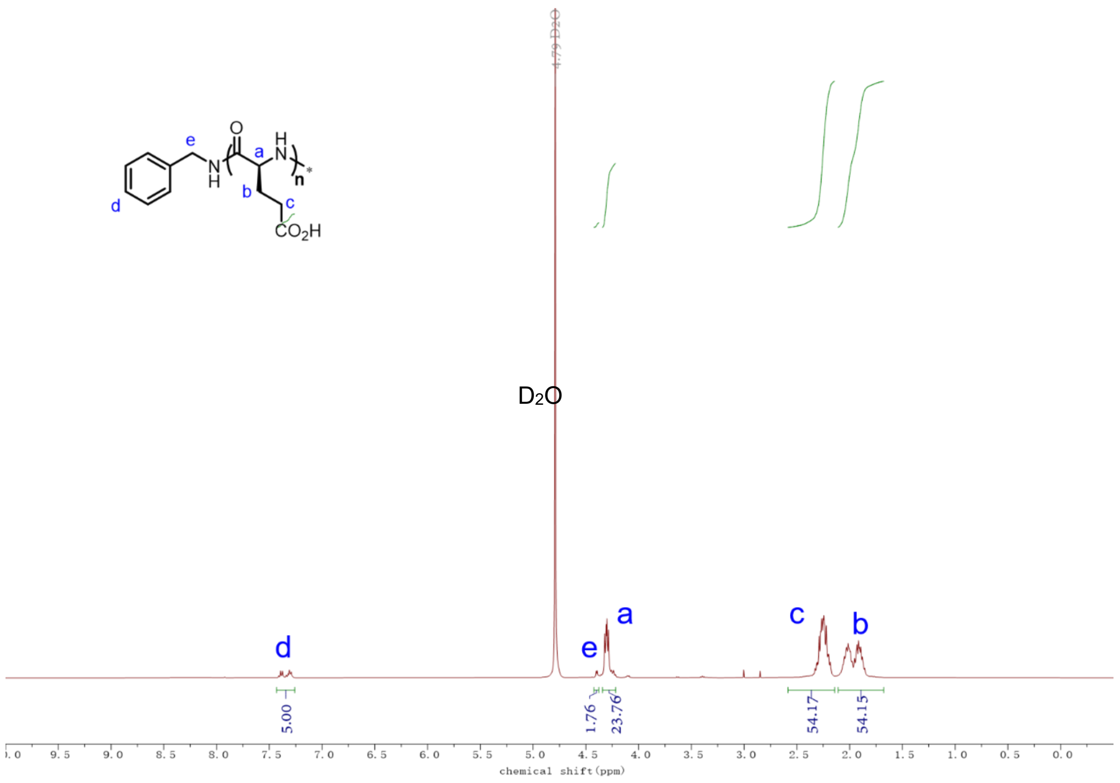

Supplementary Figure 33 ^{1}H NMR spectra of poly-L-glutamic acid. n = 20:1 in NaOD/D_2O.

D_2O

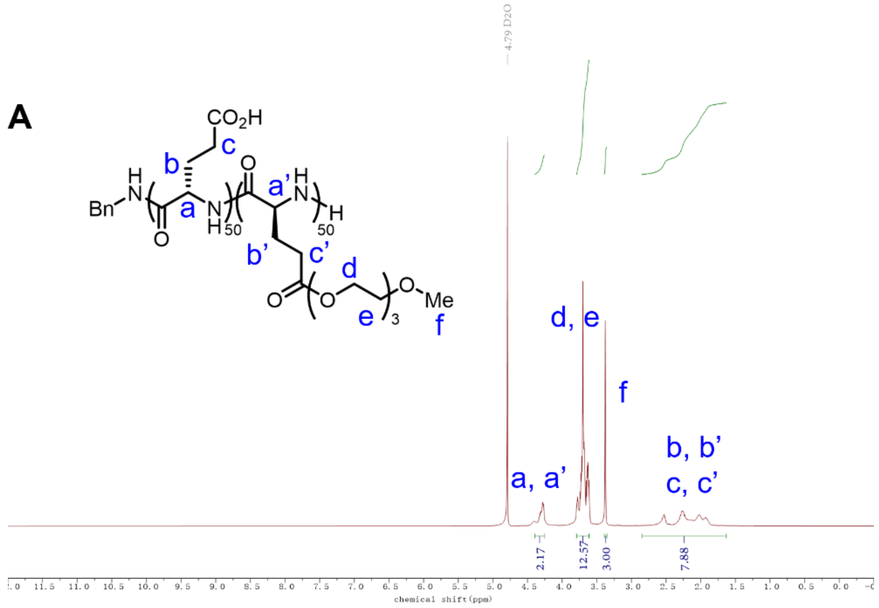

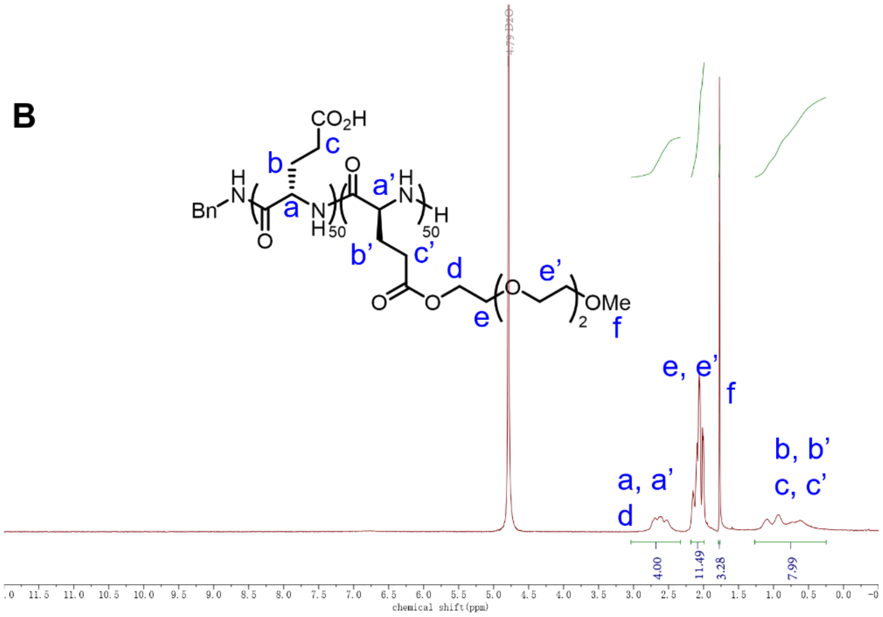

Supplementary Figure 34 ^{1}H NMR spectra of PLG-*block*-P(EG_3-Glu) in (A) NaOD/D_2O (B) TFA-*d*/D_2O

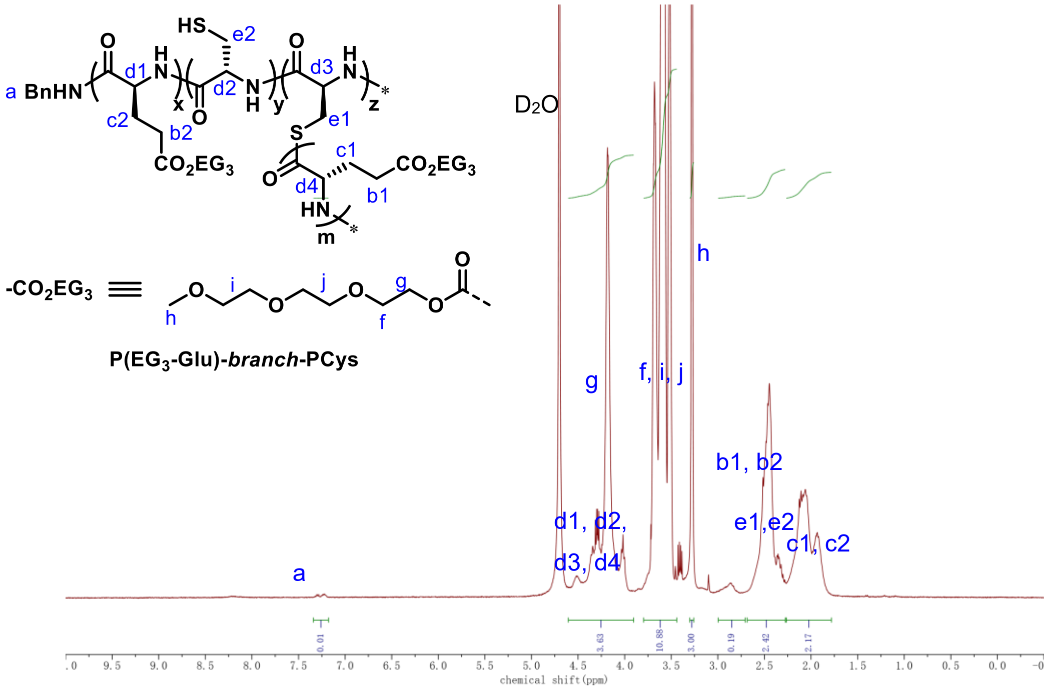

Supplementary Figure 35 ^{1}H NMR spectra of P(EG3-Glu)-*branch*-PCys in D$_2$O

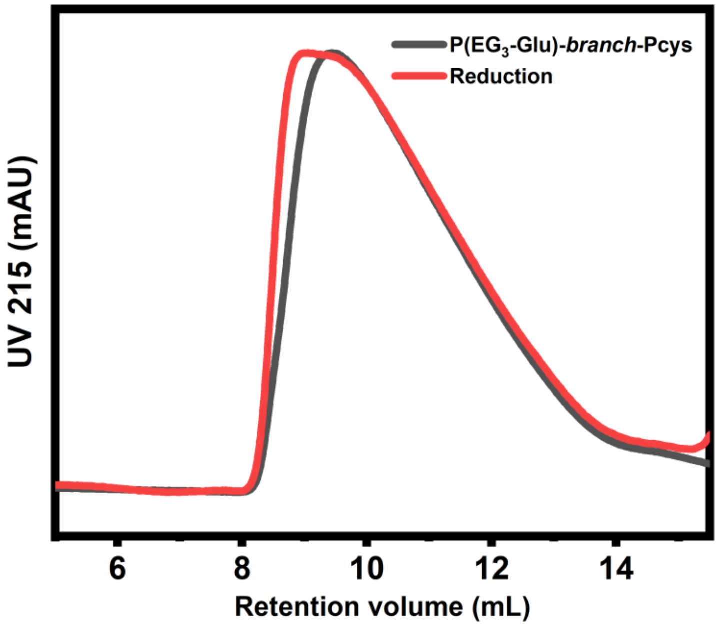

Supplementary Figure 36. Overlay of the aqueous SEC traces of P(EG$_3$-Glu)-*branch*-Pcys before (black) and after (red) treatment of TCEP.

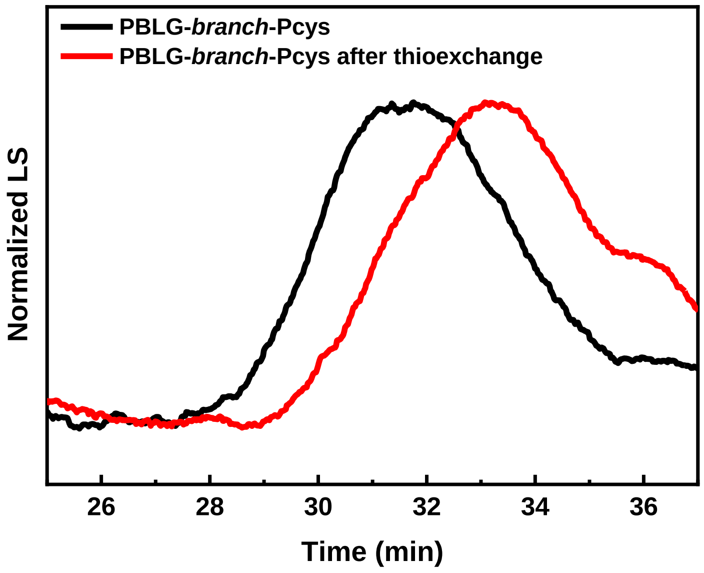

Supplementary Figure 37. DMF-phased SEC of PBLG-*branch*-Pcys and corresponding thioexchange.

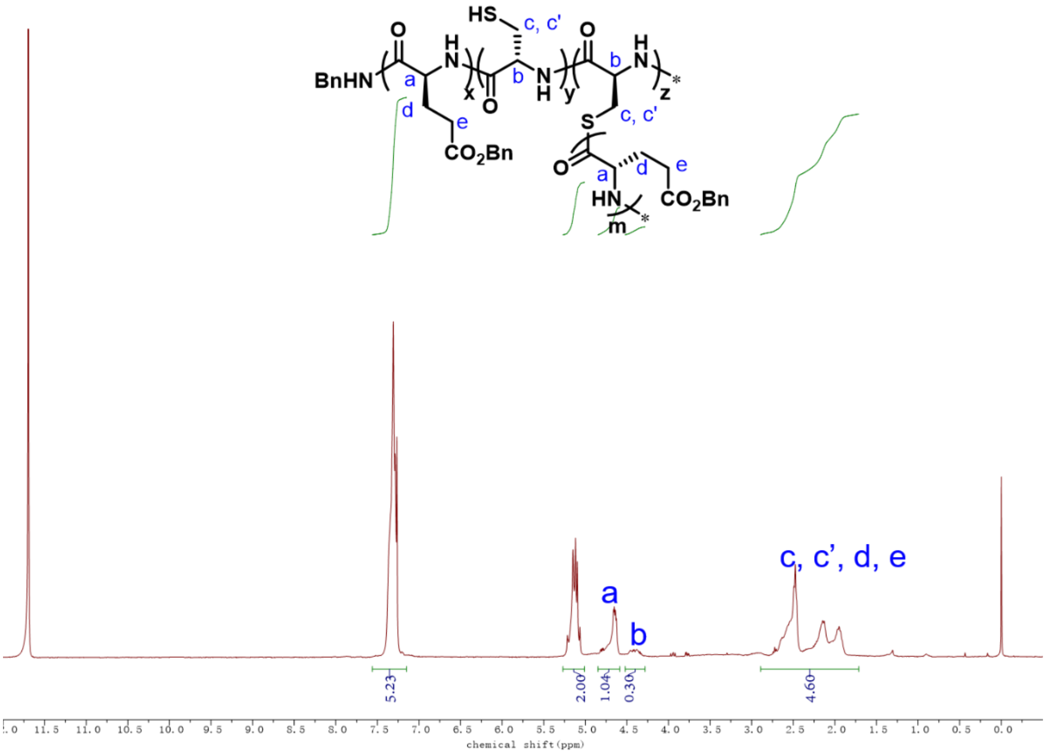

Supplementary Figure 38. ^{1}H NMR spectra of PBLG-*branch*-Pcys

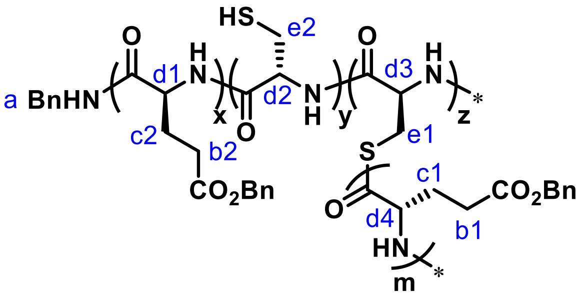

PBLG-*branch*-Pcys

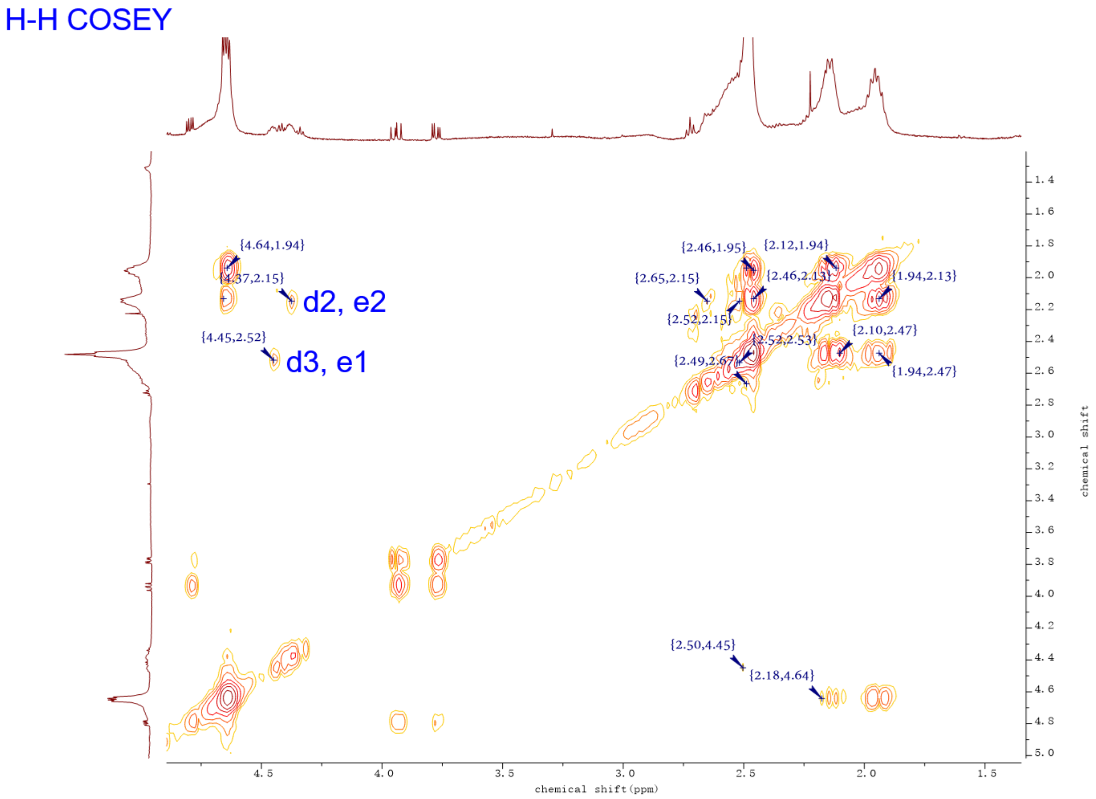

Supplementary Figure 39. H-H COSEY spectra of PBLG-*branch*-Pcys

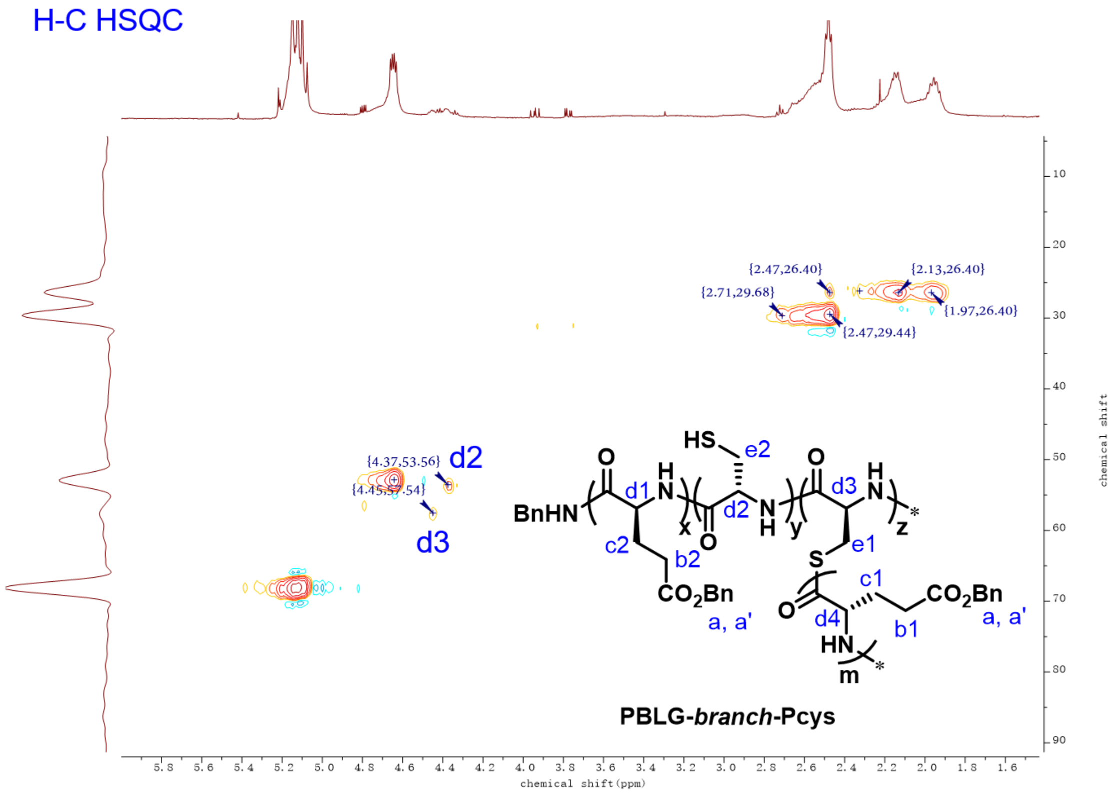

Supplementary Figure 40. H-C HSQC spectra of PBLG-*branch*-Pcys

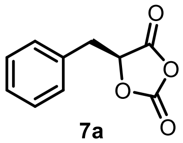

7a

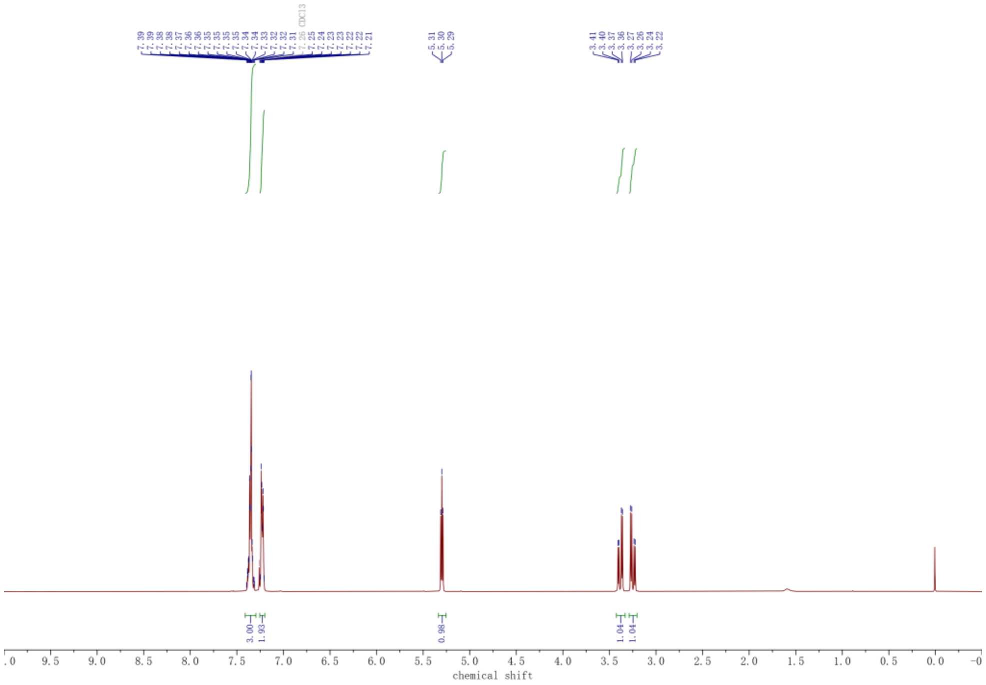

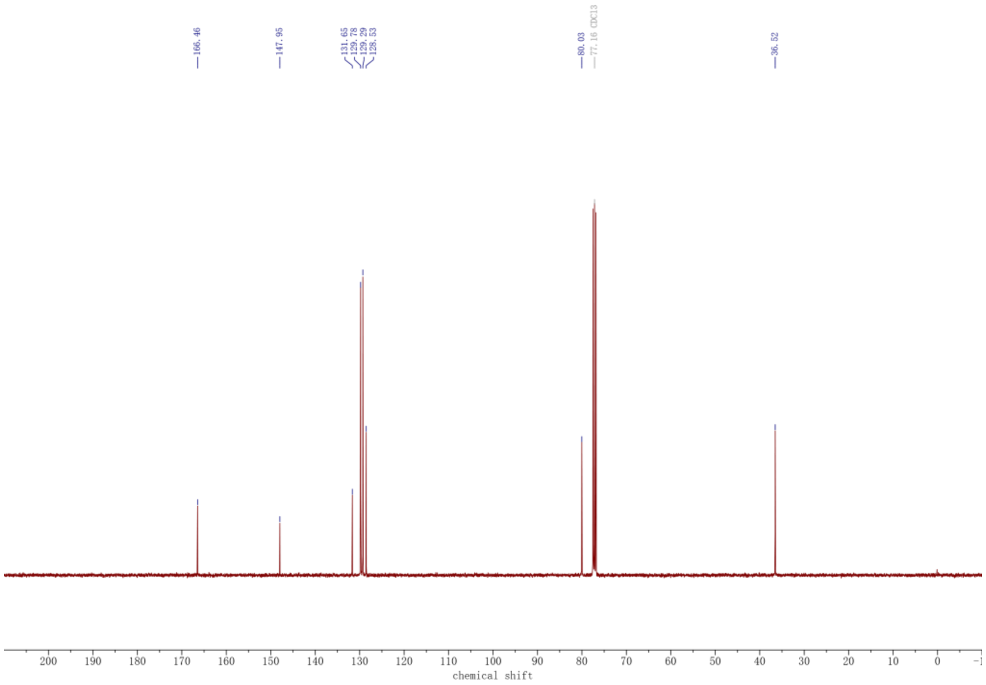

Supplementary Figure 40 ^{1}H and ^{13}C NMR spectra of 7a in $CDCl_3$.

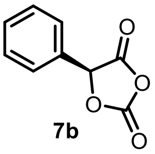

7b

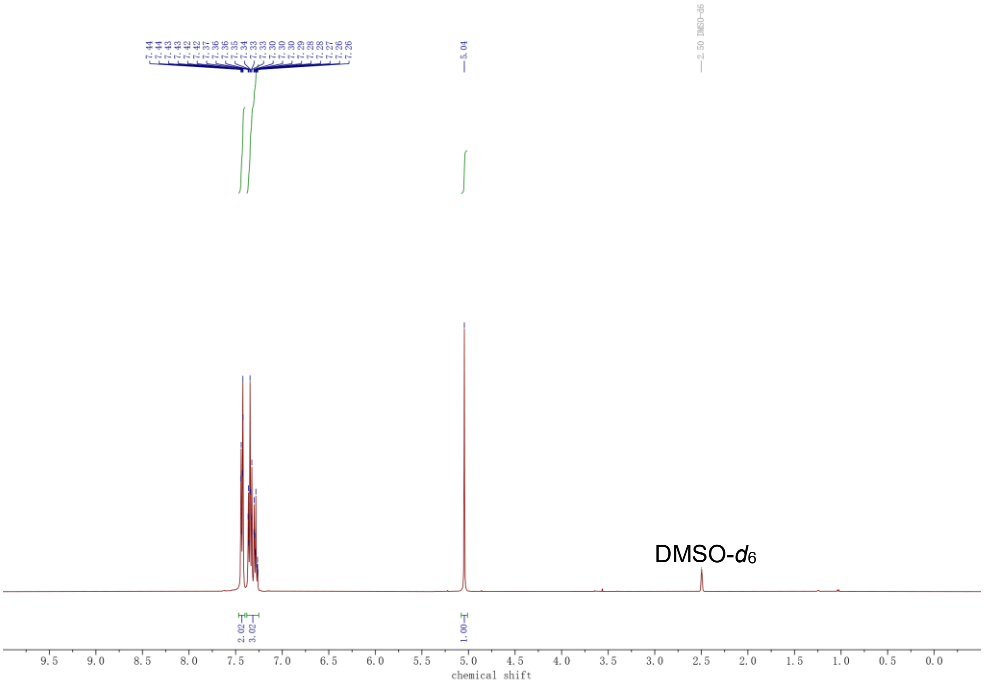

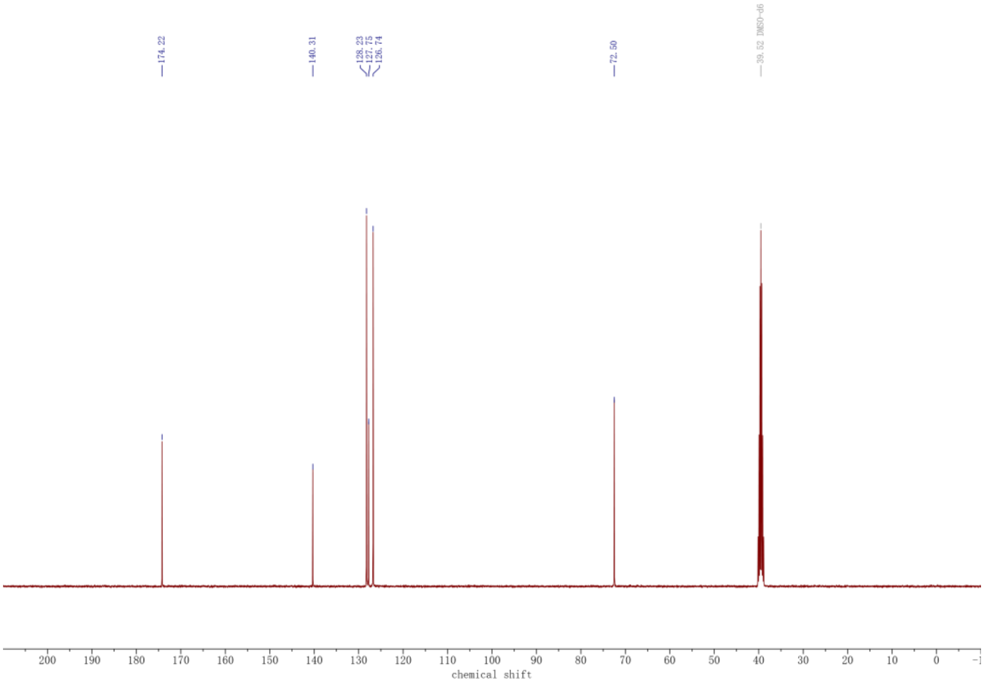

Supplementary Figure 42. **^{1}H and ^{13}C NMR spectra of 7b in DMSO-*d*$_6$.**

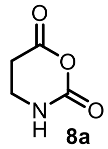

8a

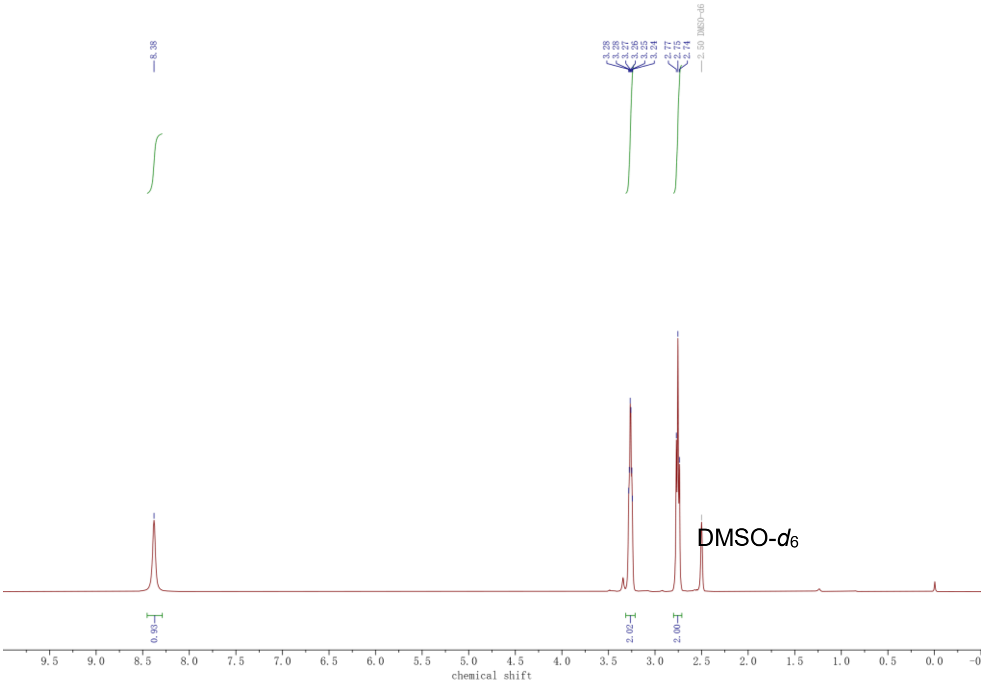

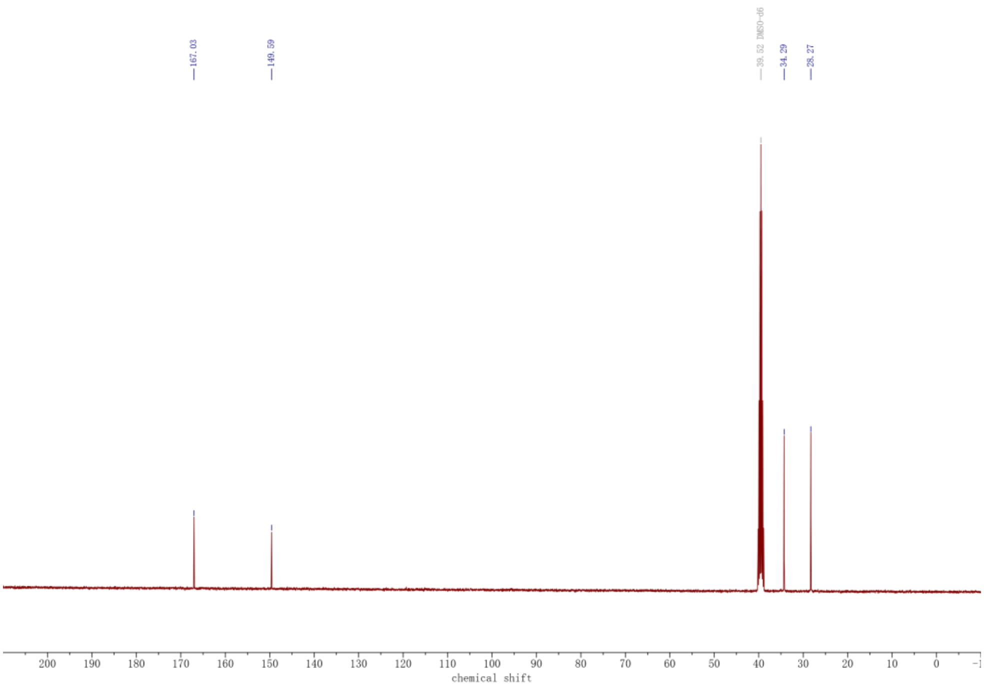

Supplementary Figure 43. ^{1}H and ^{13}C NMR spectra of 8a in DMSO-d_6.

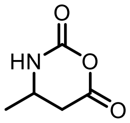

8b

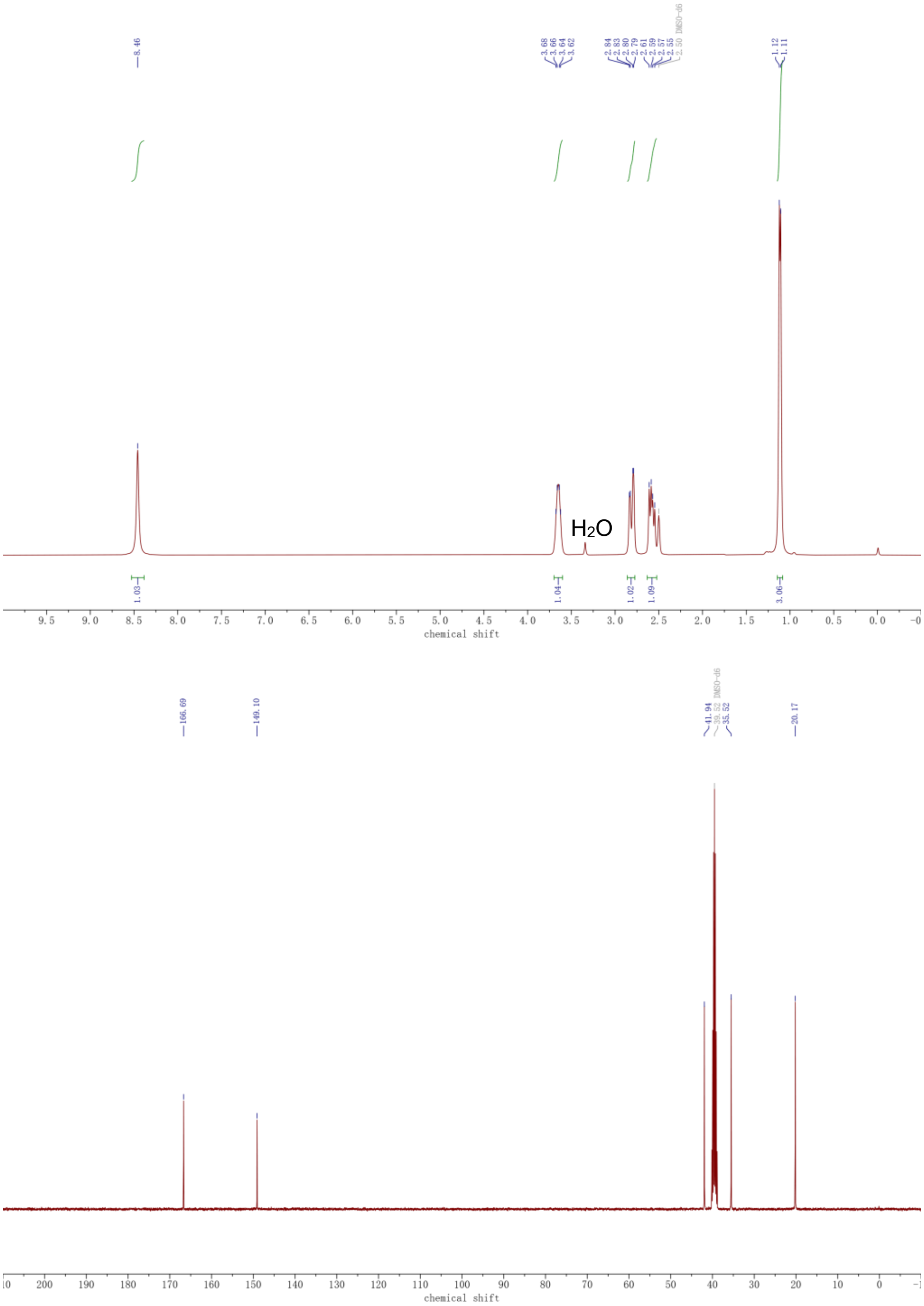

Supplementary Figure 44. ^{1}H and ^{13}C NMR spectra of 8b in DMSO-d_6.

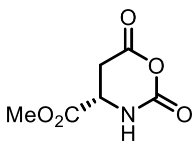

8c

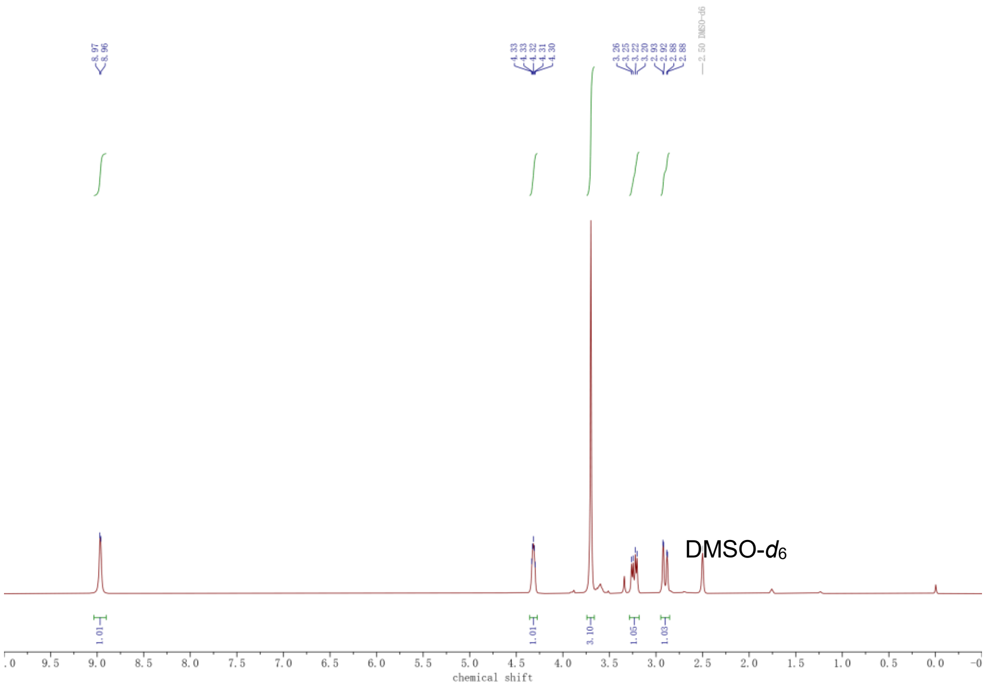

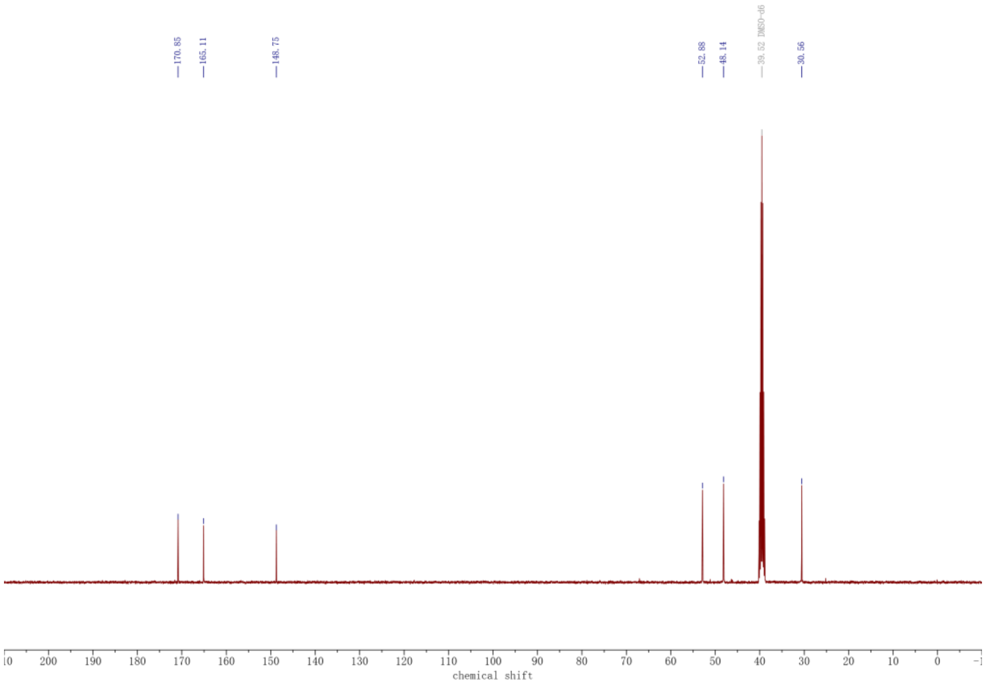

Supplementary Figure 45. ^{1}H and ^{13}C NMR spectra of 8c in DMSO-d_6.

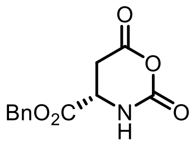

8d

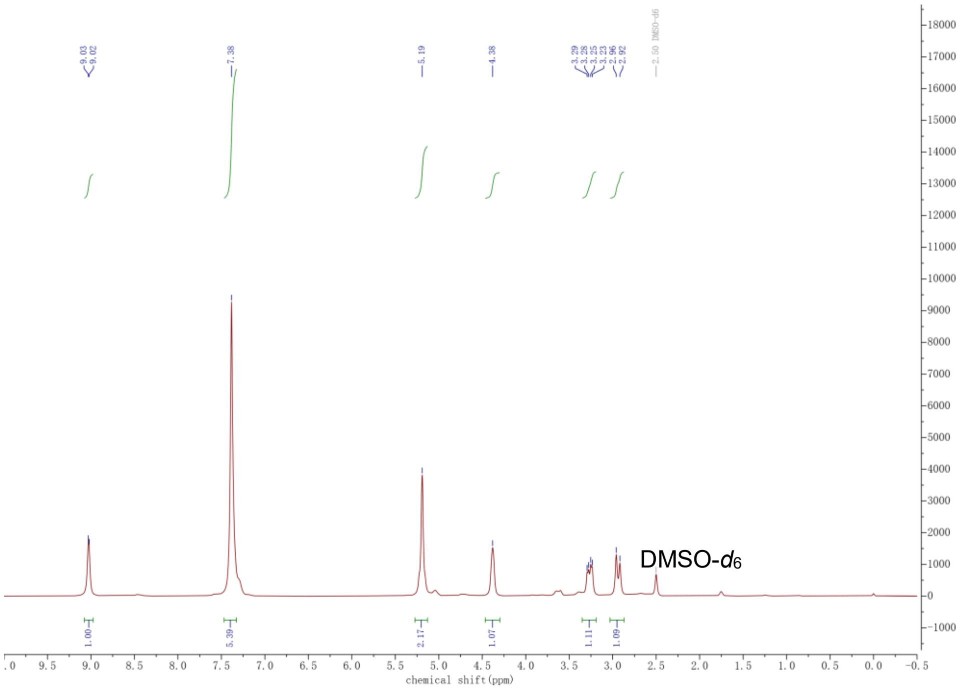

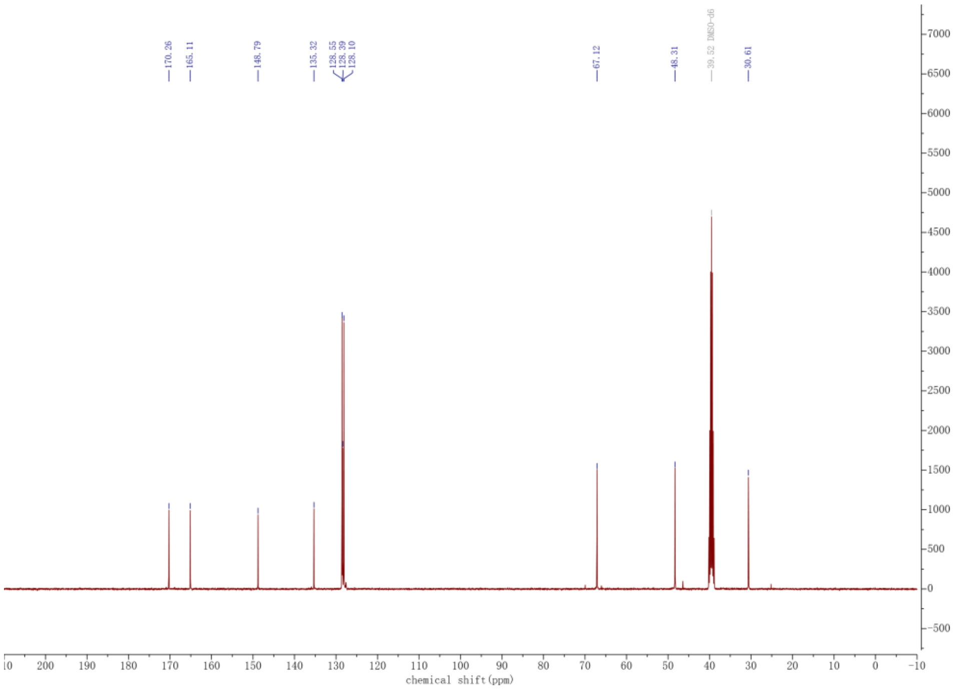

Supplementary Figure 46. ^{1}H and ^{13}C NMR spectra of 8d in DMSO-*d*$_6$.

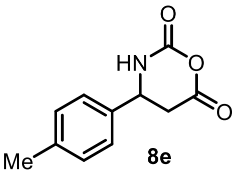

8e

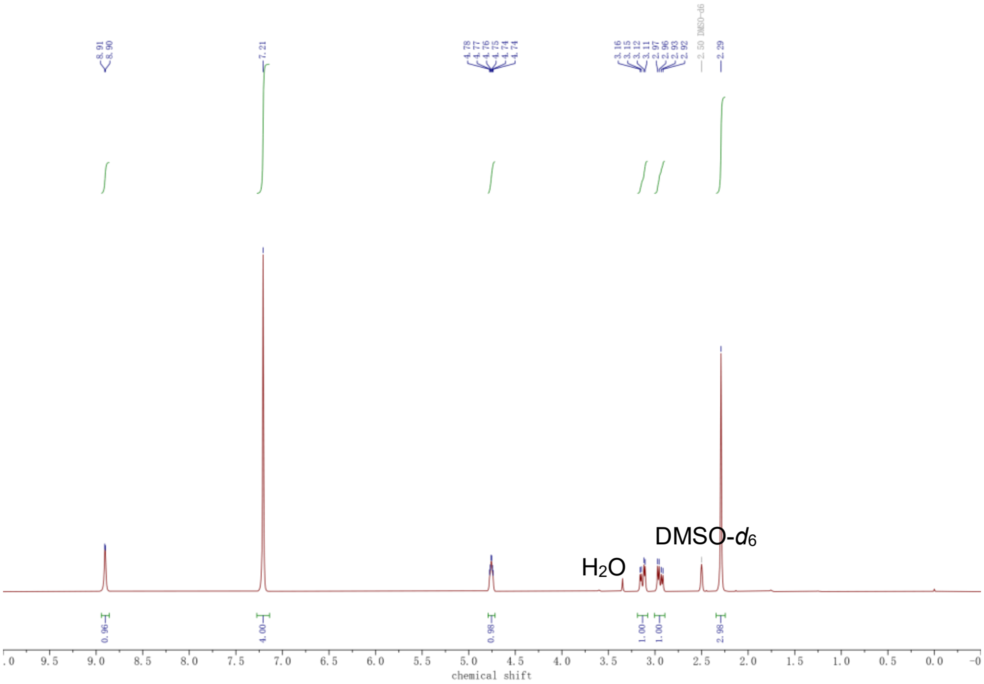

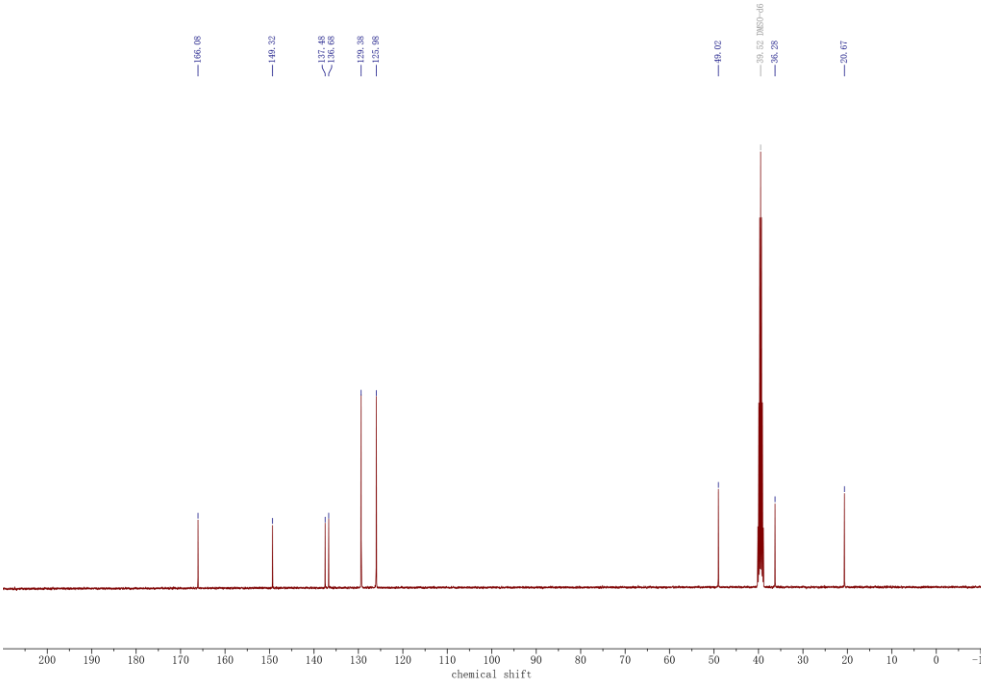

Supplementary Figure 47. ^{1}H and ^{13}C NMR spectra of 8e in DMSO-d_6.

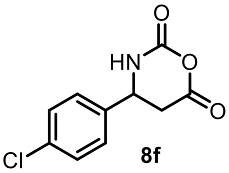

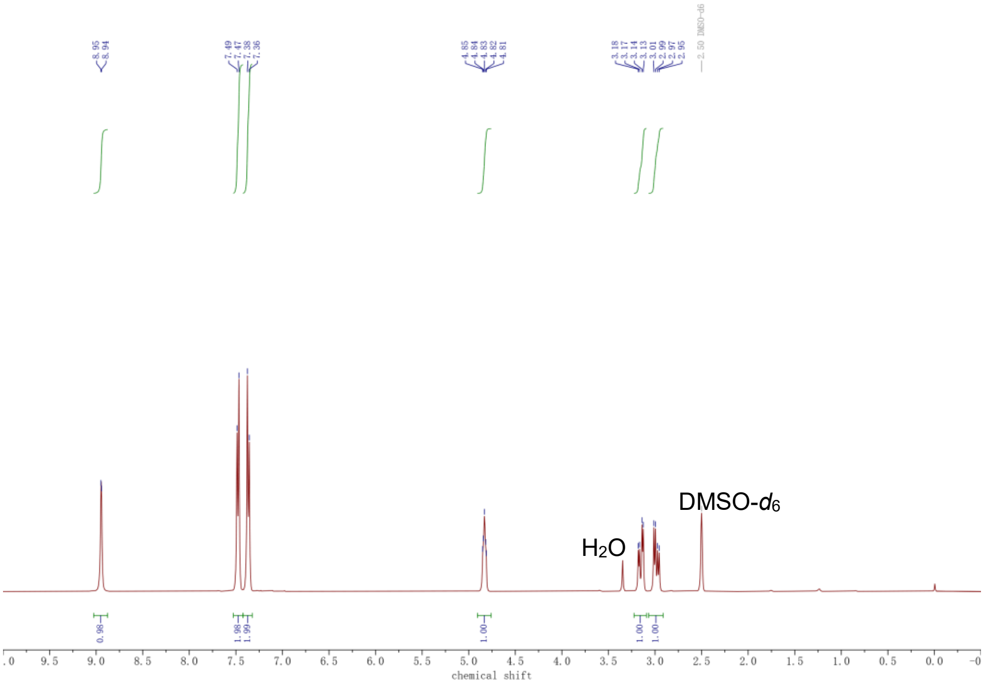

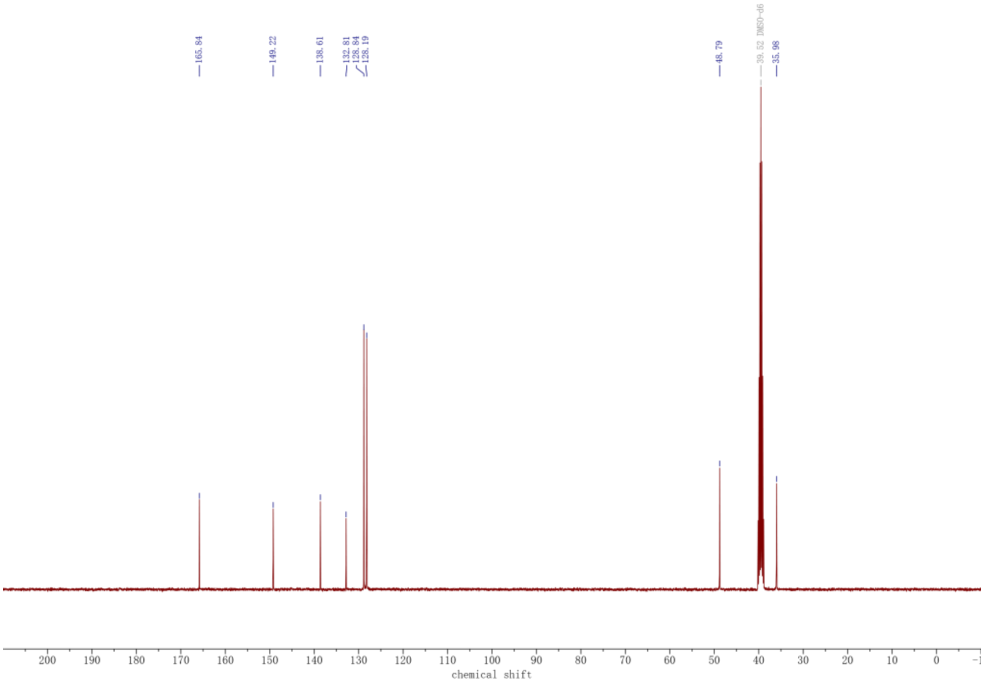

Supplementary Figure 48. ^{1}H and ^{13}C NMR spectra of 8f in DMSO-d_6.

X-Ray Crystal (Supplementary Figure 44-46)

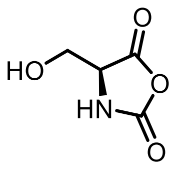

3p
CCDC 2052323

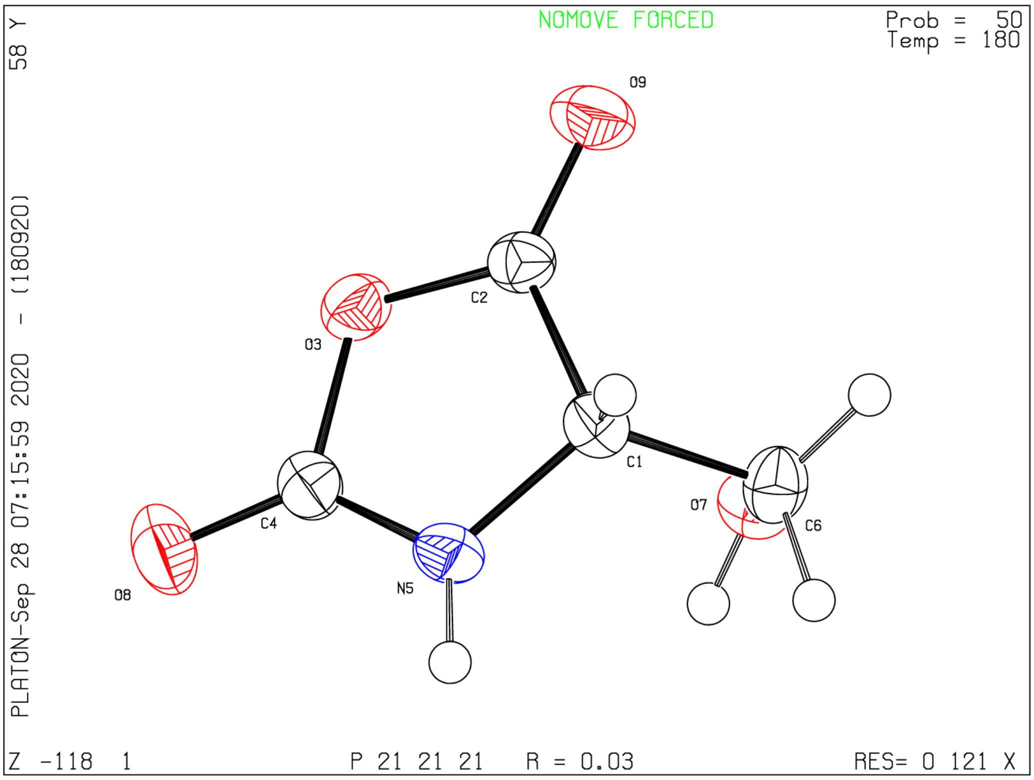

Supplementary Figure 49. Crystal structure of 3p.

https://www.ccdc.cam.ac.uk/structures/Search?ccdc=2052323

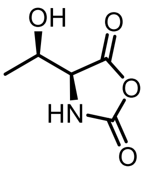

3q
CCDC 2052324

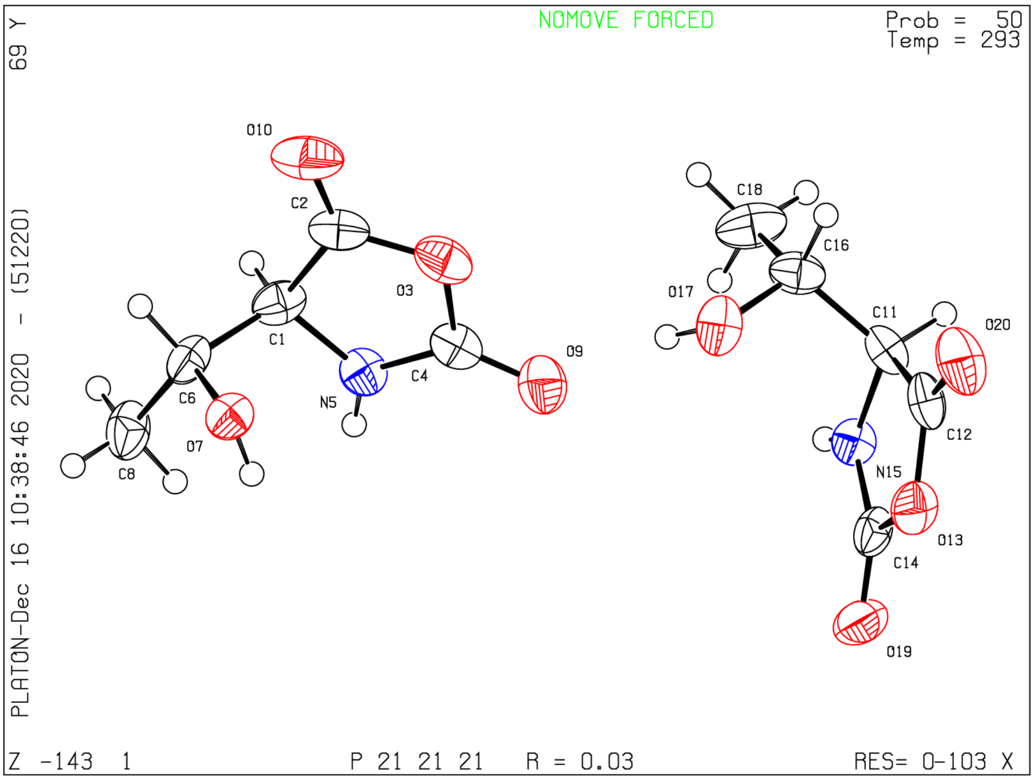

Supplementary Figure 50. Crystal structure of 3q.

https://www.ccdc.cam.ac.uk/structures/Search?ccdc=2052324

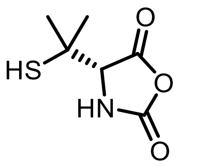

3s
CCDC 2052321

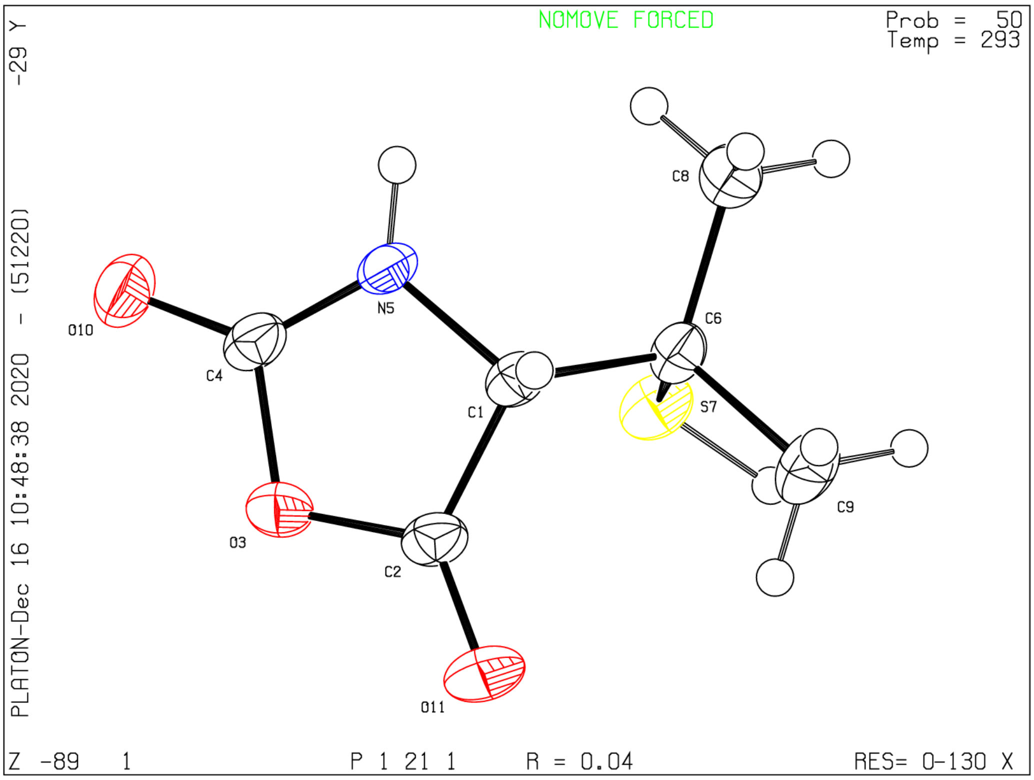

Supplementary Figure 51. Crystal structure of 3s.

https://www.ccdc.cam.ac.uk/structures/Search?ccdc=2052321

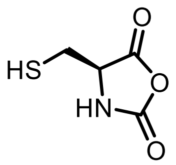

3r
CCDC 2088683

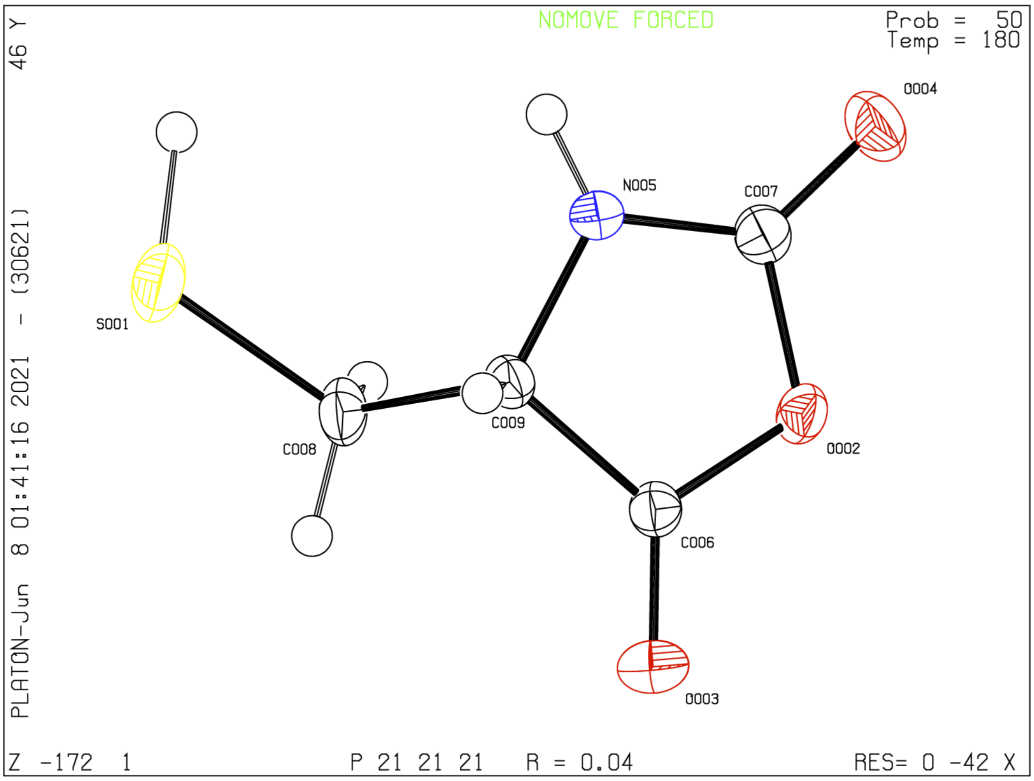

Supplementary Figure 52. Crystal structure of 3r

https://www.ccdc.cam.ac.uk/structures/Search?ccdc=2088683

Data and Coordinates of All Stationary Points

Stationary points	TCG[a]	SPE[b]	G
**L-Alanine**	0.078579	-323.7647612	-323.6861822
**NCA Product**	0.064433	-435.9204356	-435.8560026
**Phosgene**	-0.017597	-1033.731283	-1033.74888
**Epoxide**	0.033553	-153.7901143	-153.7565613
**HCl**	-0.011244	-460.8037458	-460.8149898
**Int0a**	0.076477	-1357.494906	-1357.418429
**Int0b**	0.078125	-1357.491365	-1357.41324
**Int1a**	0.083436	-1357.506999	-1357.423563
**Int1b**	0.083465	-1357.492371	-1357.408906
**Int2b**	0.080305	-1357.52089	-1357.440585
**Int3a**	0.075301	-1357.534521	-1357.45922
**Int4a**	0.073074	-896.7207426	-896.6476686
**Int4c**	0.127111	-1050.522804	-1050.395693
**Int5a**	0.07583	-896.7195369	-896.6437069
**Int5c**	0.124367	-1050.52902	-1050.404653
**TS1a**	0.079213	-1357.490516	-1357.411303
**TS1b**	0.081937	-1357.484455	-1357.402518
**TS2a**	0.08423	-1357.504967	-1357.420737
**TS2b**	0.084401	-1357.491973	-1357.407572
**TS3a**	0.072616	-896.6739646	-896.6013486
**TS3c**	0.123849	-1050.486486	-1050.362637
**TS4a**	0.072879	-896.6824523	-896.6095733
**TS4c**	0.124239	-1050.510353	-1050.386114

[a]TCG = Thermal Corrections to Gibbs Energies, computed at the B3LYP/6-31G(d) level
[b]SPE = Single Point Energy, computed at the M06-2X/def2-TZVPP//B3LYP/6-31G(d) level
All data were in Hartree unit.

Int2b

Standard orientation:

Center Number	Atomic Number	Atomic Type	Coordinates (Angstroms) X	Y	Z
1	6	0	-1.121547	1.061949	-0.527774
2	7	0	-2.444272	0.995216	0.164423
3	8	0	1.040568	0.139843	-0.271001
4	6	0	-0.095428	0.501098	0.445801
5	6	0	-0.758653	2.488458	-0.959791
6	8	0	-0.240231	0.396538	1.628588
7	6	0	2.217010	-0.141521	0.366881
8	8	0	2.523715	0.115073	1.483263
9	17	0	3.273038	-0.940697	-0.799365
10	1	0	-1.188399	0.400547	-1.394749
11	1	0	-2.361875	1.274329	1.148250
12	1	0	-3.135130	1.599108	-0.289558
13	1	0	0.206681	2.481999	-1.471556
14	1	0	-1.509620	2.866249	-1.659346
15	1	0	-0.697243	3.163062	-0.100197
16	1	0	-2.815873	-0.035932	0.124460
17	17	0	-3.239470	-1.846797	-0.152738

##

L-Alanine

Standard orientation:

Center Number	Atomic Number	Atomic Type	Coordinates (Angstroms) X	Y	Z
1	6	0	-0.646472	-0.025266	0.380173
2	7	0	-1.085573	1.331244	-0.001427
3	8	0	1.448129	1.022982	-0.196943
4	6	0	0.858544	-0.151174	0.057171
5	6	0	-1.452141	-1.172863	-0.233068
6	8	0	1.465311	-1.203118	0.061931
7	1	0	-0.708643	-0.090333	1.473946
8	1	0	-1.505577	1.324109	-0.930102
9	1	0	-1.789339	1.686667	0.640210
10	1	0	-1.027726	-2.136116	0.061187
11	1	0	-2.493002	-1.128642	0.102253

12	1	0	-1.437725	-1.115727	-1.328009
13	1	0	0.693909	1.678246	-0.155057

##

Epoxide

Standard orientation:

Center Number	Atomic Number	Atomic Type	Coordinates (Angstroms) X	Y	Z
1	6	0	-0.667408	-0.483400	0.000000
2	6	0	0.000000	0.823985	0.000000
3	8	0	0.765408	-0.390631	0.000000
4	1	0	-1.108828	-0.863991	0.920666
5	1	0	-1.108828	-0.863991	-0.920666
6	1	0	0.049422	1.404759	0.920642
7	1	0	0.049422	1.404759	-0.920642

##

HCl

Standard orientation:

Center Number	Atomic Number	Atomic Type	Coordinates (Angstroms) X	Y	Z
1	17	0	0.000000	0.000000	0.071711
2	1	0	0.000000	0.000000	-1.219092

##

NCA Product

Standard orientation:

Center Number	Atomic Number	Atomic Type	Coordinates (Angstroms) X	Y	Z
1	6	0	0.827411	-0.610572	0.452890
2	7	0	-0.540665	-1.074483	0.300041
3	8	0	-0.674594	1.100313	-0.160394
4	6	0	0.653273	0.864572	0.102290
5	6	0	1.848731	-1.310355	-0.448567
6	8	0	1.491174	1.721621	0.038302

7	6	0	-1.413906	-0.095645	-0.026744
8	8	0	-2.605869	-0.125881	-0.200935
9	1	0	1.150568	-0.674999	1.500389
10	1	0	-0.857810	-2.012775	0.499307
11	1	0	2.825328	-0.832486	-0.330536
12	1	0	1.940666	-2.363095	-0.165928
13	1	0	1.547165	-1.251685	-1.498516

##

Phosgene

Standard orientation:

Center Number	Atomic Number	Atomic Type	Coordinates (Angstroms) X	Y	Z
1	6	0	-0.000083	0.498656	-0.000005
2	8	0	-0.000097	1.681573	0.000003
3	17	0	1.463913	-0.483619	0.000000
4	17	0	-1.463838	-0.483706	0.000000

##

Int0a

Standard orientation:

Center Number	Atomic Number	Atomic Type	Coordinates (Angstroms) X	Y	Z
1	6	0	-1.657072	0.788763	-0.086009
2	7	0	-0.584306	0.817521	-1.078005
3	8	0	-2.649266	-0.965754	1.310016
4	6	0	-2.063404	-0.669785	0.128613
5	6	0	-2.914128	1.605071	-0.463013
6	8	0	-1.945581	-1.528890	-0.716726
7	1	0	-1.256214	1.178569	0.859452
8	1	0	-0.841507	0.204800	-1.851193
9	1	0	-0.489041	1.758835	-1.454897
10	1	0	-3.675413	1.569225	0.324720
11	1	0	-2.638570	2.653604	-0.617406
12	1	0	-3.354915	1.221014	-1.389308
13	1	0	-2.626984	-0.198791	1.908369
14	6	0	1.643332	-0.160989	0.511821
15	8	0	0.918704	-0.353354	1.428375

16	17	0	2.058262	-1.380989	-0.689436
17	17	0	2.549359	1.339561	0.280289

###

TS1a

Standard orientation:

Center Number	Atomic Number	Atomic Type	Coordinates (Angstroms) X	Y	Z
1	6	0	-1.437161	0.758371	-0.124250
2	7	0	-0.244320	0.528041	-0.936573
3	8	0	-2.909981	-0.680638	1.202002
4	6	0	-2.085847	-0.602399	0.139519
5	6	0	-2.463773	1.705156	-0.778768
6	8	0	-1.933903	-1.557930	-0.588439
7	1	0	-1.111081	1.182163	0.834222
8	1	0	-0.425310	-0.217481	-1.609576
9	1	0	0.040273	1.368130	-1.436342
10	1	0	-3.335278	1.850963	-0.132047
11	1	0	-2.006252	2.683776	-0.954810
12	1	0	-2.806634	1.299257	-1.735803
13	1	0	-2.885467	0.138577	1.727511
14	6	0	1.368589	-0.146894	0.471406
15	8	0	0.757112	-0.389918	1.462380
16	17	0	2.021614	-1.435766	-0.608464
17	17	0	2.369170	1.361696	0.314809

###

Int1b

Standard orientation:

Center Number	Atomic Number	Atomic Type	Coordinates (Angstroms) X	Y	Z
1	6	0	-2.343408	0.596381	-0.206658
2	7	0	-3.509458	-0.369261	-0.168089
3	8	0	-1.206082	-1.483346	-0.611463
4	6	0	-1.096796	-0.297971	-0.366166
5	6	0	-2.334976	1.515925	1.009468
6	8	0	0.002299	0.422249	-0.216128
7	6	0	1.326996	-0.219817	-0.466453

8	8	0	1.496612	-0.893177	-1.442193
9	17	0	2.449220	1.380361	-0.290455
10	1	0	-2.452633	1.175648	-1.127639
11	1	0	-3.797018	-0.578098	0.794197
12	1	0	-4.330352	-0.024558	-0.673766
13	1	0	-1.490682	2.202352	0.926985
14	1	0	-3.255695	2.104473	1.054448
15	1	0	-2.223755	0.947218	1.938517
16	1	0	-3.180458	-1.257193	-0.590467
17	17	0	1.647443	-1.140239	1.301718

###

Int0b

Standard orientation:

Center Number	Atomic Number	Atomic Type	Coordinates (Angstroms) X	Y	Z
1	6	0	-2.834425	0.513323	-0.235129
2	7	0	-3.809747	-0.631122	-0.420972
3	8	0	-1.464969	-1.439661	-0.353732
4	6	0	-1.424814	-0.183878	-0.181341
5	6	0	-3.147756	1.344672	1.000945
6	8	0	-0.453197	0.565540	0.013668
7	6	0	2.164448	-0.273272	-0.404324
8	8	0	1.962799	-0.862696	-1.408615
9	17	0	2.637136	1.430179	-0.344443
10	1	0	-2.885150	1.129048	-1.136004
11	1	0	-4.432948	-0.766994	0.378221
12	1	0	-4.378985	-0.561654	-1.266148
13	1	0	-2.359751	2.092017	1.115815
14	1	0	-4.110663	1.859093	0.915416
15	1	0	-3.155839	0.723328	1.904369
16	1	0	-3.101681	-1.440179	-0.489256
17	17	0	2.197655	-1.025905	1.193438

###

TS1b

Standard orientation:

Center Number	Atomic Number	Atomic Type	Coordinates (Angstroms) X	Y	Z

1	6	0	-2.519133	0.547987	-0.232678
2	7	0	-3.567603	-0.544036	-0.297267
3	8	0	-1.213308	-1.446600	-0.467073
4	6	0	-1.157333	-0.217991	-0.272819
5	6	0	-2.700190	1.446538	0.983092
6	8	0	-0.148929	0.535257	-0.112941
7	6	0	1.659307	-0.257443	-0.400979
8	8	0	1.660607	-0.885653	-1.407784
9	17	0	2.480378	1.387173	-0.311936
10	1	0	-2.609166	1.124425	-1.156376
11	1	0	-4.057388	-0.674807	0.592194
12	1	0	-4.267705	-0.403100	-1.028710
13	1	0	-1.892675	2.180977	0.991500
14	1	0	-3.656126	1.978194	0.949348
15	1	0	-2.643178	0.871403	1.913821
16	1	0	-2.984771	-1.400838	-0.494373
17	17	0	1.813820	-1.069913	1.238768

###

Int1a

Standard orientation:

Center Number	Atomic Number	Atomic Type	Coordinates (Angstroms) X	Y	Z
1	6	0	-1.251738	0.641717	-0.224376
2	7	0	0.003604	0.010413	-0.753339
3	8	0	-3.129219	-0.115355	0.998253
4	6	0	-2.167262	-0.514689	0.167178
5	6	0	-1.903067	1.544104	-1.278614
6	8	0	-2.042877	-1.637621	-0.276936
7	1	0	-0.979825	1.218530	0.660159
8	1	0	-0.256882	-0.911319	-1.140643
9	1	0	0.435597	0.571280	-1.492198
10	1	0	-2.817461	1.975569	-0.864162
11	1	0	-1.227556	2.363642	-1.542249
12	1	0	-2.160700	0.983238	-2.182226
13	1	0	-3.713268	-0.877349	1.183190
14	6	0	1.064632	-0.264804	0.385981
15	8	0	0.635906	-0.750200	1.399233
16	17	0	2.418065	-1.216651	-0.590874
17	17	0	1.848346	1.580825	0.554761

##

TS2a

Standard orientation:

Center Number	Atomic Number	Atomic Type	Coordinates (Angstroms) X	Y	Z
1	6	0	1.188670	0.644945	0.139757
2	7	0	-0.025971	-0.018251	0.735840
3	8	0	3.163098	-0.088835	-0.931302
4	6	0	2.189588	-0.477591	-0.111234
5	6	0	1.741733	1.705722	1.095793
6	8	0	2.105998	-1.566864	0.419827
7	1	0	0.870679	1.098688	-0.798530
8	1	0	0.304042	-0.806321	1.319968
9	1	0	-0.568058	0.651645	1.294918
10	1	0	2.623864	2.162635	0.640972
11	1	0	0.993666	2.487438	1.254689
12	1	0	2.030432	1.271609	2.058055
13	1	0	3.799920	-0.824560	-1.028208
14	6	0	-0.973724	-0.585277	-0.322712
15	8	0	-0.562568	-0.893762	-1.395290
16	17	0	-2.345700	-1.365693	0.495145
17	17	0	-1.913275	1.763076	-0.462689

##

Int5a

Standard orientation:

Center Number	Atomic Number	Atomic Type	Coordinates (Angstroms) X	Y	Z
1	6	0	-0.115400	-1.154264	-0.455158
2	7	0	1.180719	-1.077938	0.208931
3	8	0	-1.147529	0.669628	-1.612007
4	6	0	-0.465701	0.351063	-0.482965
5	6	0	-1.100231	-2.111012	0.198672
6	8	0	0.763482	1.026900	-0.378886
7	1	0	0.009882	-1.430075	-1.512132
8	1	0	1.778397	-1.884680	0.328163
9	1	0	-2.078196	-2.027893	-0.284712

10	1	0	-0.747211	-3.139398	0.071562
11	1	0	-1.208456	-1.904139	1.265268
12	1	0	-1.318688	1.628572	-1.629940
13	6	0	1.744161	0.155260	0.097133
14	8	0	2.863115	0.528943	0.349890
15	17	0	-1.465310	0.885478	1.016541

##

Int4a

Standard orientation:

Center Number	Atomic Number	Atomic Type	Coordinates (Angstroms) X	Y	Z
1	6	0	-0.846382	0.851191	0.498885
2	7	0	0.541415	0.738830	0.949299
3	8	0	-2.664694	-0.462022	-0.297279
4	6	0	-1.495113	-0.535496	0.365343
5	6	0	-1.054115	1.788791	-0.700560
6	8	0	-1.058318	-1.557190	0.844497
7	1	0	-1.365783	1.306639	1.350687
8	1	0	0.734091	1.039466	1.897874
9	1	0	-2.122701	1.956215	-0.852120
10	1	0	-0.576392	2.749647	-0.489737
11	1	0	-0.632453	1.380129	-1.619941
12	1	0	-3.052142	-1.358945	-0.298459
13	6	0	1.603363	0.109579	0.405192
14	8	0	2.687642	-0.026612	0.908024
15	17	0	1.309524	-0.538986	-1.275793

##

TS3a

Standard orientation:

Center Number	Atomic Number	Atomic Type	Coordinates (Angstroms) X	Y	Z
1	6	0	-1.173850	0.068624	0.911841
2	7	0	0.141900	-0.339933	1.404484
3	8	0	-2.297228	-0.433490	-1.154890
4	6	0	-1.206245	-0.580981	-0.449188
5	6	0	-1.441091	1.579354	0.912093

6	8	0	-0.218758	-1.231527	-0.809519
7	1	0	-1.935079	-0.433558	1.523085
8	1	0	0.524336	0.120602	2.225205
9	1	0	-2.438398	1.769474	0.505923
10	1	0	-1.417872	1.948922	1.941511
11	1	0	-0.689943	2.100505	0.316761
12	1	0	-2.205223	-0.888725	-2.017042
13	6	0	1.050065	-0.805920	0.539127
14	8	0	2.028108	-1.445563	0.504694
15	17	0	1.629331	1.240029	-0.831315

##

Int5c

Standard orientation:

Center Number	Atomic Number	Atomic Type	Coordinates (Angstroms) X	Y	Z
1	6	0	-1.935304	0.811061	0.207416
2	7	0	-2.424764	-0.489007	-0.238139
3	8	0	0.352428	1.198388	0.918656
4	6	0	-0.417860	0.556191	0.044315
5	6	0	-2.334411	1.150604	1.645741
6	8	0	-0.254122	-0.840385	0.075264
7	1	0	-2.262699	1.599286	-0.472255
8	1	0	-3.403918	-0.739117	-0.238939
9	1	0	-1.913086	2.116431	1.936420
10	1	0	-3.425337	1.213488	1.713832
11	1	0	-1.982205	0.387732	2.346187
12	1	0	1.294475	0.856038	0.882389
13	6	0	-1.479614	-1.461425	-0.176824
14	8	0	-1.575043	-2.657556	-0.310273
15	17	0	0.080090	1.071824	-1.754129
16	6	0	3.517971	-0.110091	-0.318111
17	6	0	3.089312	-1.124210	0.650722
18	8	0	2.872982	0.284782	0.918749
19	1	0	4.566050	0.173313	-0.376458
20	1	0	2.924601	0.060586	-1.212990
21	1	0	3.821822	-1.588431	1.306497
22	1	0	2.181602	-1.691891	0.463755

##

Int4c

Standard orientation:

Center Number	Atomic Number	Atomic Type	Coordinates (Angstroms) X	Y	Z
1	6	0	-0.973599	1.260211	-0.296141
2	7	0	-1.977859	0.673918	0.582295
3	8	0	1.289272	1.050186	-0.961425
4	6	0	0.402099	0.613374	-0.071256
5	6	0	-0.917854	2.777992	-0.065515
6	8	0	0.642232	-0.187215	0.814408
7	1	0	-1.260145	1.071813	-1.333125
8	1	0	-2.081231	1.099451	1.498101
9	1	0	-0.186262	3.226745	-0.740047
10	1	0	-1.898212	3.221494	-0.260387
11	1	0	-0.624619	3.010152	0.964582
12	1	0	2.177292	0.619744	-0.775925
13	6	0	-2.538225	-0.550639	0.525627
14	8	0	-3.224354	-1.075904	1.361160
15	17	0	-2.226514	-1.413307	-1.060511
16	6	0	3.616008	-1.458189	0.013065
17	6	0	3.956864	-0.362520	0.924490
18	8	0	3.679765	-0.093446	-0.473132
19	1	0	4.398531	-2.102343	-0.380199
20	1	0	2.613599	-1.876325	0.049986
21	1	0	4.992541	-0.192795	1.208397
22	1	0	3.197187	0.000510	1.611546

##

TS3c

Standard orientation:

Center Number	Atomic Number	Atomic Type	Coordinates (Angstroms) X	Y	Z
1	6	0	-1.422088	1.286568	-0.332714
2	7	0	-2.154067	0.435985	0.605212
3	8	0	0.953634	1.563192	-0.630377
4	6	0	0.009696	0.873829	-0.078693
5	6	0	-1.695769	2.773654	-0.103569
6	8	0	0.188320	-0.126986	0.641728
7	1	0	-1.652755	1.002593	-1.368991

8	1	0	-3.163824	0.391230	0.491640
9	1	0	-1.098611	3.366621	-0.800678
10	1	0	-2.751821	2.989808	-0.287508
11	1	0	-1.449244	3.066219	0.920678
12	1	0	1.873049	1.145124	-0.443132
13	6	0	-1.592953	-0.760291	0.896413
14	8	0	-1.689097	-1.602979	1.706671
15	17	0	-1.491498	-1.851010	-1.242683
16	6	0	3.361118	-0.950789	-0.416803
17	6	0	3.499085	-0.357890	0.915958
18	8	0	3.286633	0.492623	-0.244896
19	1	0	4.243092	-1.276050	-0.961375
20	1	0	2.421738	-1.422940	-0.692515
21	1	0	4.483874	-0.243384	1.360260
22	1	0	2.657981	-0.401230	1.602195

##

TS4c

Standard orientation:

Center Number	Atomic Number	Atomic Type	Coordinates (Angstroms) X	Y	Z
1	6	0	1.845073	-0.836238	-0.370655
2	7	0	2.434449	0.417532	0.081284
3	8	0	-0.445995	-1.519242	0.147909
4	6	0	0.448265	-0.644295	0.166482
5	6	0	2.525715	-2.090835	0.194232
6	8	0	0.351077	0.448977	0.892751
7	1	0	1.788766	-0.873079	-1.460918
8	1	0	3.329123	0.764742	-0.237208
9	1	0	1.984129	-2.981843	-0.134641
10	1	0	3.549198	-2.146980	-0.186130
11	1	0	2.555514	-2.067692	1.287071
12	1	0	-1.462177	-1.188274	0.499978
13	6	0	1.591447	1.213936	0.758703
14	8	0	1.682528	2.302687	1.242763
15	17	0	-0.397704	0.745350	-2.172173
16	6	0	-3.379643	-0.030462	-0.185521
17	6	0	-3.023592	0.497104	1.132835
18	8	0	-2.712108	-0.899505	0.802799
19	1	0	-4.398886	-0.346750	-0.382315
20	1	0	-2.748695	0.218605	-1.037410

21	1	0	-3.775193	0.575835	1.911593
22	1	0	-2.149561	1.133168	1.231695

###

TS4a

Standard orientation:

Center Number	Atomic Number	Atomic Type	Coordinates (Angstroms) X	Y	Z
1	6	0	0.004605	-0.747191	-0.653260
2	7	0	0.997490	0.226220	-1.104016
3	8	0	-0.335511	-1.430172	1.651491
4	6	0	0.259449	-0.675853	0.804578
5	6	0	0.114253	-2.136135	-1.286160
6	8	0	1.108126	0.219132	1.150754
7	1	0	-1.022789	-0.311457	-0.764341
8	1	0	1.041588	0.561209	-2.059072
9	1	0	-0.631079	-2.799106	-0.839569
10	1	0	-0.097779	-2.056311	-2.354971
11	1	0	1.111905	-2.561303	-1.149076
12	1	0	-0.098385	-1.214971	2.579682
13	6	0	1.620171	0.896340	-0.132957
14	8	0	2.404481	1.779793	-0.038423
15	17	0	-2.629031	1.072083	-0.128725

###

TS2b

Standard orientation:

Center Number	Atomic Number	Atomic Type	Coordinates (Angstroms) X	Y	Z
1	6	0	2.327269	0.617069	0.181177
2	7	0	3.488201	-0.354118	0.171274
3	8	0	1.189183	-1.421570	0.756073
4	6	0	1.083948	-0.256303	0.437442
5	6	0	2.287963	1.450605	-1.094656
6	8	0	-0.017750	0.467079	0.272872
7	6	0	-1.326943	-0.140570	0.533924
8	8	0	-1.518421	-0.851335	1.475479
9	17	0	-2.465552	1.337633	0.249272

10	1	0	2.468182	1.257085	1.056594
11	1	0	4.330713	0.031412	0.607409
12	1	0	3.184432	-1.204885	0.678211
13	1	0	1.465196	2.164192	-1.027579
14	1	0	3.220980	2.007784	-1.216371
15	1	0	2.124462	0.821718	-1.975278
16	1	0	3.731998	-0.641726	-0.783085
17	17	0	-1.558029	-1.192631	-1.362303

##

Supplementary References

[1] Sun, Y.; Hou, Y.; Zhou, X.; Yuan, J.; Wang, J.; Lu, H. *ACS Macro Lett.* **2015**, *4*, 1000.

[2] Gaussian 09, Revision E.01, M. J. Frisch, G. W. Trucks, H. B. Schlegel, G. E. Scuseria, M. A. Robb, J. R. Cheeseman, G. Scalmani, V. Barone, B. Mennucci, G. A. Petersson, H. Nakatsuji, M. Caricato, X. Li, H. P. Hratchian, A. F. Izmaylov, J. Bloino, G. Zheng, J. L. Sonnenberg, M. Hada, M. Ehara, K. Toyota, R. Fukuda, J. Hasegawa, M. Ishida, T. Nakajima, Y. Honda, O. Kitao, H. Nakai, T. Vreven, J. A. Montgomery, Jr., J. E. Peralta, F. Ogliaro, M. Bearpark, J. J. Heyd, E. Brothers, K. N. Kudin, V. N. Staroverov, T. Keith, R. Kobayashi, J. Normand, K. Raghavachari, A. Rendell, J. C. Burant, S. S. Iyengar, J. Tomasi, M. Cossi, N. Rega, J. M. Millam, M. Klene, J. E. Knox, J. B. Cross, V. Bakken, C. Adamo, J. Jaramillo, R. Gomperts, R. E. Stratmann, O. Yazyev, A. J. Austin, R. Cammi, C. Pomelli, J. W. Ochterski, R. L. Martin, K. Morokuma, V. G. Zakrzewski, G. A. Voth, P. Salvador, J. J. Dannenberg, S. Dapprich, A. D. Daniels, O. Farkas, J. B. Foresman, J. V. Ortiz, J. Cioslowski, and D. J. Fox, Gaussian, Inc., Wallingford CT, 2013.

[3] (a) Becke, A. D. *J. Chem. Phys.* **1993**, *98*, 5648. (b) Lee, C.; Yang, W.; Parr, R. G. *Phys. Rev. B* **1988**, *37*, 785.

[4] Marenich, A. V.; Cramer, C. J.; Truhlar, D. G. *J. Phys. Chem. B* **2009,** *113*, 6378.

[5] (a) Rassolov, V. A.; Ratner, M. A.; Pople, J. A.; Redfern, P. C.; Curtiss, L. A. *J. Comp. Chem.* **2001**, *22***,** 976. (b) Rassolov, V. A.; Pople, J. A.; Ratner, M. A.; Windus, T. L. *J. Chem. Phys.* **1998**, *109*, 1223.

[6] Zhao, Y.; Truhlar, D. G. *Theor. Chem. Acc.* **2007**, *120*, 215.

[7] (a) Weigend, F.; Ahlrichs, R. *Phys. Chem. Chem. Phys.* **2005,** *7,* 3297; (b) Weigend, F. *Phys. Chem. Chem. Phys.* **2006,** *8*, 1057.
